# Total Synthesis and Analysis of Phenolic Phytoprostanes, Oxidized Derivatives of Lipophenols

**DOI:** 10.1002/chem.202502206

**Published:** 2025-10-15

**Authors:** M. Jordan Lehoux, Ángel Sánchez‐Illana, Pablo Miralles, Valérie Bultel‐Poncé, Thierry Durand, Céline Crauste, Camille Oger

**Affiliations:** ^1^ Institut des Biomolécules Max Mousseron IBMM Université de Montpellier CNRS ENSCM 1919 route de Mende Montpellier 34293 France; ^2^ Department of Analytical Chemistry University of Valencia 50 Dr. Moliner Burjassot 46100 Spain; ^3^ Foundation for the Promotion of Health and Biomedical Research of the Valencian Community FISABIO‐Public Health 21 Av. Catalunya Valencia 46020 Spain

**Keywords:** hydroxytyrosol, lipidomics, lipophenols, phytoprostanes, total synthesis

## Abstract

Lipophenols, or phenolipids, are compounds that combine polyphenols with fatty acids, offering the antioxidant properties of polyphenols alongside the neuroprotective and cardioprotective benefits of omega‐3 polyunsaturated fatty acids (PUFAs). Hydroxytyrosol (HT)‐based lipophenols have recently been identified in olive oil. Depending on their structure, these compounds were differently affected by storage conditions. While the concentrations of HT‐oleate and HT‐linoleate increase with higher storage temperatures, HT‐α‐linolenate (HT‐ALA) did not follow the same trend. Given the high oxidative susceptibility of α‐linolenic acid (ALA), which leads to oxidation products known as phytoprostanes (PhytoPs), we hypothesize that HT‐ALA may be oxidized into covalently bound PhytoP conjugates of HT (PhytoPs‐HT) under various oxidative conditions, including, for example, suboptimal storage. This study aims to develop the first total synthesis of two series of PhytoPs‐HT as analytical standards to explore the oxidative transformation of HT‐ALA lipophenols into potential oxylipin metabolites. The synthesis of four ALA‐derived PhytoPs‐HT and their identification during in vitro oxidation of HT‐ALA by UHPLC‐HRMS/MS are presented here. These synthetic standards are essential for the reliable identification and quantification of HT‐PhytoPs within complex mixtures of regio‐ and stereoisomers generated during oxylipin formation.

## Graphical abstract

The total synthesis of four hydroxytyrosol‐phytoprostanes (PhytoPs‐HT) was achieved in 20 steps, enabling their first identification as HT‐α‐linolenate oxidation products. This work revealed new oxidized derivatives of lipophenols via UHPLC‐MS/MS analysis, offering a strategy to trace HT‐derived compounds in complex matrices, aiding food and biomedical research.

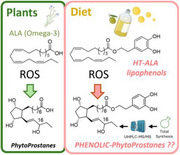

## 1 Introduction

Lipophenols, also known as phenolipids, are hybrid molecules that combine (poly)phenols with fatty acids. These compounds have been developed for therapeutic, food, and cosmetic applications.^[^ ^1^, ^2^ ^]^ One key advantage of lipophenols is their ability to enhance the typically low bioavailability of polyphenols.^[^ ^3^ ^]^ Additionally, they hold promise for therapeutic synergy by merging the diverse activities of polyphenols, particularly their antioxidant properties,^[^ ^4^, ^5^ ^]^ with the beneficial effects of polyunsaturated fatty acids (PUFAs), such as their cardio‐ and neuroprotective effects.^[^ ^6^ ^]^ For instance, earlier research has demonstrated the potent bioactivity of synthetic alkyl‐lipophenols in neurodegenerative diseases,^[^ ^7^, ^8^ ^]^ while the bioactivities of natural lipophenols include antioxidant protection for the skin^[^ ^9^ ^]^ and anti‐inflammatory effects.^[^ ^10^ ^]^ Further studies have also confirmed the bioaccessibility of lipophenols during gastrointestinal digestion,^[^ ^11^, ^12^ ^]^ as well as their potential formation by intestinal enzymes.^[^ ^13^ ^]^

Several studies have documented the natural occurrence of lipophenols (as esters) in plant‐based sources, including apple skin,^[^ ^14^ ^]^ green tea,^[^ ^15^ ^]^ and oils,^[^ ^9^, ^16^, ^17^ ^]^ such as supplemented flaxseed oil^[^ ^16^ ^]^ and fish oil.^[^ ^18^ ^]^ These findings underscore the dietary presence of these hybrid molecules and encourage further exploration of their biological activity and metabolites.

**Figure 1:**
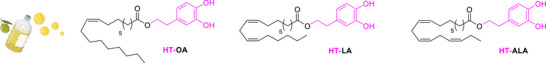
Chemical structures of hydroxytyrosol lipophenols identified in olive oil.

In 2016 and 2020, hydroxytyrosol (HT) lipophenols were identified in olive oils through HPLC‐MS/MS analysis.^[^ ^16^, ^19^ ^]^ Three specific HT lipophenols were detected, linked to oleic acid (OA, C18:1 n‐9; HT‐OA), linoleic acid (LA, C18:2 n‐6; HT‐LA), and α‐linolenic acid (ALA, C18:3 n‐3; HT‐ALA) (Figure 1).

**Scheme 1:**
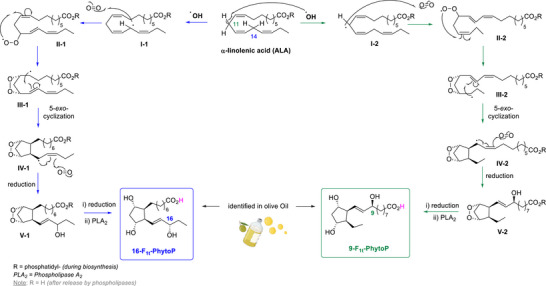
The biosynthesis of F‐type phytoprostanes (F‐PhytoPs) from ALA identified in olive oil. F‐PhytoPs are defined by the presence of a cyclopentan‐1,3‐diol ring.

During a study assessing the metabolic fate of olive oil lipophenols under various storage conditions,^[^ ^20^ ^]^ HT‐ALA displayed a degradation profile different from that observed for HT‐OA and HT‐LA. This may be attributed to structural differences within the fatty acid moieties (Figure 1). Indeed, due to its two bis‐allylic positions, at C11 and C14 (Scheme 1), ALA is highly prone to oxidation, leading to the formation of a diverse set of oxygenated derivatives,^[^ ^21^ ^]^ including phytoprostanes (PhytoPs) (Scheme 1).^[^ ^22^, ^23^ ^]^ PhytoPs are formed exclusively through nonenzymatic free radical‐mediated oxidation, cyclization, and rearrangement as depicted in Scheme 1. First, free radicals such as the hydroxyl radical (•OH) abstract a hydrogen atom from a bis‐allylic position (C11 or C14), generating carbon‐centered radicals **I‐1** or **I‐2**. These radicals then trap molecular oxygen to form peroxyl radicals (**II‐1** or **II‐2**). A 5‐*exo*‐trig cyclization gives rise to endoperoxide intermediates (**III‐1** or **III‐2**), which carry a radical capable of trapping a second molecule of oxygen. This process produces hydroperoxide intermediates that, after reduction, yield endoperoxides **V‐1** and **V‐2**. A subsequent reduction, followed by membrane cleavage via phospholipase A2, leads to the formation of phytoprostanes (PhytoPs) of the 9‐ and 16‐series. It is worth noting that PhytoPs are classified into various types and series depending on their oxidation pathways,^[^ ^24^, ^25^, ^26^ ^]^ therefore a comprehensive nomenclature was established for the isoprostanoid structures.^[^ ^26^, ^27^ ^]^ It should be mentioned that the F‐type of PhytoPs is the most commonly found in plant matrices.^[^ ^28^ ^]^ It includes two series, the 16‐series (in blue, Scheme 1) and the 9‐series (in green, Scheme 1), which arise from the initial H‐abstraction at the *bis*‐allylic positions of ALA, on carbons C14 and C11, respectively.

**Scheme 2:**
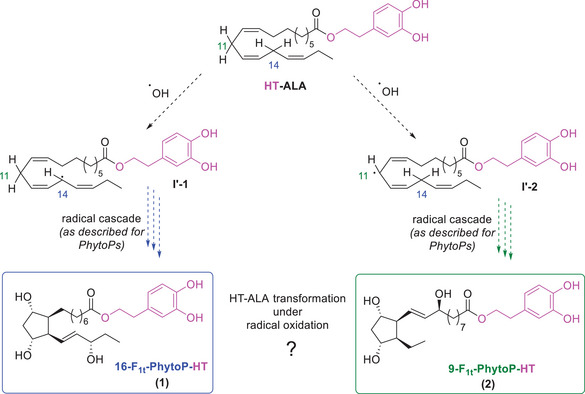
Proposed oxidative pathways and chemical structures of F‐type PhytoPs‐HT arising from the radical oxidation of HT‐ALA that follows the biosynthesis of PhytoPs as described in Scheme 1.

PhytoPs exhibit a broad spectrum of biological activities, including anti‐inflammatory^[^ ^29^ ^]^ and neuroprotective effects.^[^ ^30^ ^]^ Furthermore, they were also identified in olive oil by Dominguez‐Perles *et al.* ^[^ ^31^ ^]^ and Collado‐Gonzales *et al.*,^[^ ^32^ ^]^ demonstrating the possible oxidation of ALA within an oil matrix. These findings suggest that ALA linked to hydroxytyrosol (HT‐ALA) may undergo radical oxidation to form PhytoPs‐HT conjugates (Scheme 2). In this context, the present study aimed to develop the first total synthesis of F‐type PhytoPs‐HT conjugates from both the 9 and 16‐series to produce analytical standards enabling their future identification in vegetal matrices. Furthermore, as a proof of concept, the newly synthesized compounds were subsequently employed as standards for their detection by UHPLC‐MS/MS in in vitro oxidation samples of HT‐ALA.

## 2 Results and Discussion

### 2.1 Retrosynthetic Analysis

**Scheme 3:**
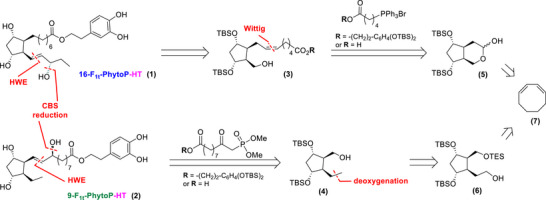
Retrosynthetic plan to access 16‐F_1t_‐PhytoP‐HT **(1)** and 9‐F_1t_‐PhytoP‐HT **(2)**.

The syntheses of 16‐F_1t_‐PhytoP‐HT and 9‐F_1t_‐PhytoP‐HT were designed with a late‐stage introduction of the second lateral chains using Horner‐Wadsworth‐Emmons (HWE) olefination and Corey‐Bakshi‐Shibata (CBS) reduction (Scheme 3). Intermediates **(3)** and **(4)**, which incorporate the first lateral chains, could be obtained either through a Wittig coupling between lactol **(5)** and the appropriate phosphonium salts or via the deoxygenation of alcohol **(6),** respectively. Notably, the cyclopentadienyl structures **(5)** and **(6)** would be synthesized starting from commercially available cyclooctadiene, using key steps established by our group in 2008.^[^ ^33^ ^]^ This approach provides a streamlined and efficient route to the desired intermediates.

### 2.2 Common Intermediates

**Scheme 4:**
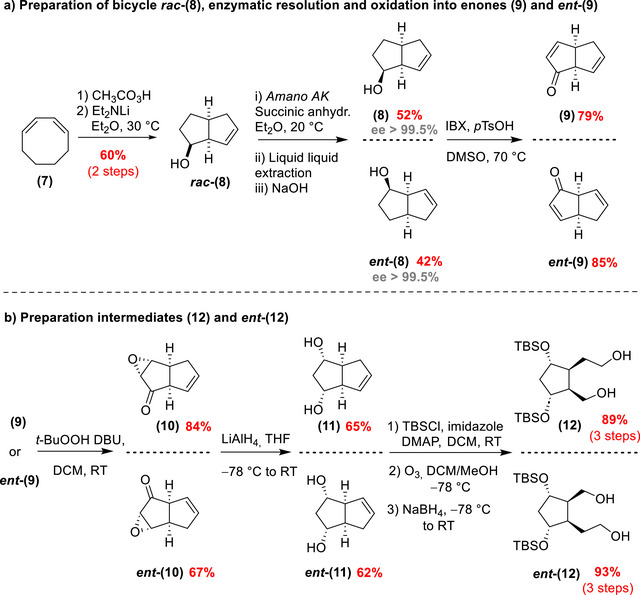
Preparation of 1,5‐diols **(12)** and ***ent*‐(12)** through the enzymatic resolution of ***rac*‐(8)**.

The synthesis of the PhytoPs‐HT began with the preparation of enantioenriched compounds **(5)** and **(6)** as key intermediates. Their synthesis starts with the formation of the tetraol intermediates **(12)** and ***ent*‐(12)** (Scheme 4).

This process started by accessing racemic bicyclo[3.3.0]oct‐7‐en‐2‐ol ***rac*‐(8)** (Scheme 4a) from commercially available cyclooctadiene. The transformation involved a mono‐epoxidation using peracetic acid, followed by a transannular CH‐insertion reaction induced by Et_2_NLi. To resolve the racemic mixture, an enzymatic resolution was performed using Amano AK lipase,^[^ ^33^ ^]^ yielding both enantiomers **(8)** and ***ent*‐(8)** separately with excellent enantiomeric excess (ee > 99.5%). Subsequently, alcohols **(8)** and ***ent*‐(8)** were oxidized to their corresponding enones **(9)** and ***ent*‐(9)** using IBX (2‐iodoxybenzoic acid) in dimethylsulfoxide (DMSO). Optimization from our earlier 2008 report introduced PTSA (*p*‐toluenesulfonic acid), which enhanced the reaction kinetics by either stabilizing the enol form of the intermediate ketone or acting as a complexing agent for IBX, as described by Nicolaou *et al.* ^[^ ^34^ ^]^ The use of PTSA allowed a reduction of the reaction temperature (from 90 °C to 70 °C) and led to improved yields.

Then, the enones were selectively epoxidized using the Yadav and Kapoor method (Scheme 4b),^[^ ^35^ ^]^ employing anhydrous *tert*‐BuOOH and 1,8‐diazabicyclo[5.4.0]undec‐7‐ene (DBU). It should be noted that by increasing the quantity of DBU (from a catalytic amount to an equivalent amount), we succeeded in scaling up the reaction (more than 5 grams). The resulting epoxyketones **(10)** and ***ent*‐(10)** were later reduced using LiAlH_4_ producing *cis*‐1,3‐bicyclic diols **(11)** and ***ent*‐(11)**. Following this, the diol functionalities were protected as a silyl ether using *tert*‐butyldimethylsilyl chloride (TBSCl), imidazole, and DMAP (4‐dimethylaminopyridine). An ozonolysis was then performed in the presence of NaBH_4_, yielding tetraol intermediates **(12)** and ***ent*‐(12)** with overall yields of 23% and 20%, respectively, over nine steps.

**Scheme 5:**
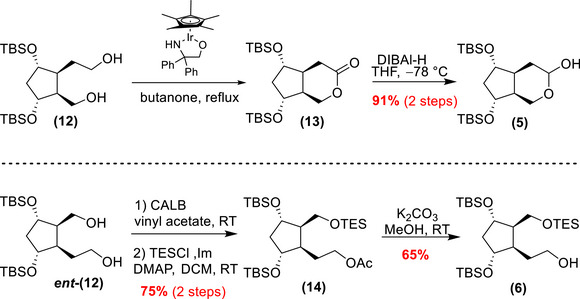
Preparation of intermediates **(5)** and **(6)**.

The preparation of key intermediates continued on both tetraol enantiomers (Scheme 5).

Tetraol **(12)** was selectively oxidized using an iridium catalyst developed by Hiroi and coworkers^[^ ^36^ ^]^ in butanone, yielding lactone **(13)** with high regioselectivity (>98/2). The lactone was then reduced with diisobutylaluminium hydride (DIBAl‐H) at low temperature, producing lactol intermediate **(5)** in 91% yield over two steps.

For the second intermediate, the process started with tetraol ***ent*‐(12)** (Scheme 5). The differentiation of its two primary alcohols was achieved using the high regioselectivity *of Candida antarctica* Lipase B (CALB), which preferentially acetylates the less sterically hindered alcohol.^[^ ^37^ ^]^ The enzymatic regioselective acetylation with vinyl acetate as the acyl donor showed complete regioselectivity, leaving the alcohol on the shorter chain free. Subsequently, the remaining alcohol was protected as triethylsilyl (TES) ether, resulting in the fully protected scaffold **(14)** in a 75% yield over two steps. The acetate group was selectively deprotected using K_2_CO_3_ in methanol to access the desired intermediate in good yield. It should be mentioned that this step required careful monitoring to avoid TES deprotection. Also, despite optimization efforts, 33% of the starting material ***ent*‐(12)** was recovered. With these two key structures in hand, the synthesis of the desired PhytoPs‐HT continued by the insertion of the lateral chains.

### 2.3 Synthesis of the 16‐F_1t_‐PhytoP‐HT and Its C16‐epimer

**Scheme 6:**
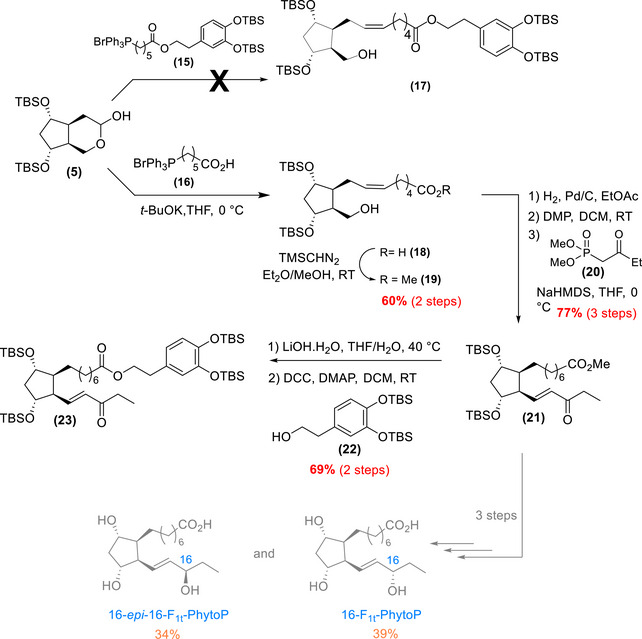
Insertion of the lateral chains and access to intermediate **(23)** and access to the 16‐F_1t_‐PhytoP and its C16‐epimer (in grey) that will serve as analytical standards (see Supporting Information for more details on the synthesis of the 16‐F_1t_‐PhytoP and the 16‐*epi*‐16‐F_1t_‐PhytoP).

The initial approach to introduce the upper lateral chain of the 16‐F_1t_‐Phyto‐HT skeleton involved a Wittig olefination using a functionalized phosphonium salt containing the HT moiety (phosphonium salt **(15)** in Scheme 6). However, despite extensive efforts, the desired olefin **(17)** could not be obtained, likely due to the instability of the ester bond linking the HT group to the alkyl structure. Consequently, the strategy shifted to the use of commercially available (5‐carboxypentyl)‐triphenylphosphonium bromide **(16)**, prior to trimethylsilyldiazomethane (TMSCHN_2_)‐mediated esterification, to afford alkene **(19)** in 60% yield over two steps. To complete this sequence, the carbon‐carbon double bond was reduced via palladium‐on‐charcoal‐catalyzed hydrogenation, ensuring the successful formation of the desired intermediate.

To install the lower lateral chain, a three‐step cascade was performed. First, the primary alcohol was oxidized using Dess‐Martin periodinane, before the HWE reaction. This reaction was performed with commercially available (2‐oxobutyl)diethyl phosphonate **(20)** in the presence of NaHMDS and yielded enone **(21)** in 77% over the three steps.^[^ ^38^ ^]^ At this stage, the HT scaffold was introduced in a two‐step sequence. The methyl ester of **(21)** was saponified using LiOH to generate the free acid, prior to a Steglich coupling with protected HT **(22)**.^[^ ^39^ ^]^ These steps yielded the full carbon framework of the desired 16‐PhytoP‐HT (compound **(23)**) with a 69% yield over two steps.

**Scheme 7:**
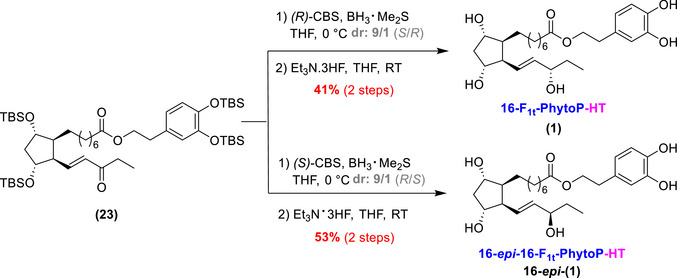
Final steps toward the 16‐F_1t_‐PhytoP‐HT **(1)** and its epimer **16‐*epi*‐(1)**.

At this junction, only two steps remained to procure the desired 16‐F_1t_‐PhytoP‐HT (Scheme 7): a diastereoselective reduction of the enone into allylic alcohol and the deprotection of the silyl groups. Therefore, two stereoselective reductions were performed on the enone **(23)** scaffold using both (*R*)‐ and (*S*)‐configured oxazaborolidines developed by Corey‐Bakshi‐Shibata (CBS) for the reductions.^[^ ^40^ ^]^ The (*R*)‐configured CBS reduction resulted in the (*S*)‐configured alcohol with a 1/9 (*R*)/(*S*) ratio, while the (*S*)‐configured CBS reduction produced the opposite configuration, as confirmed by detailed NMR analysis (see Supplementary data).^[^ ^41^ ^]^ To complete the synthesis, the TBS silyl ethers were deprotected using Et_3_N·3HF in THF, yielding the desired final compounds: 16‐F_1t_‐PhytoP‐HT **(1)** and 16‐*epi*‐16‐F_1t_‐PhytoP‐HT **16‐*epi*‐(1)** in 41% and 53% yield over two steps, respectively. It is worth noting that the separation of the remaining 10% of the minor epimer proved challenging, which accounts for the moderate yields.

### 2.4 Synthesis of the 9‐F_1t_‐PhytoP‐HT and its C9‐Epimer

**Scheme 8:**
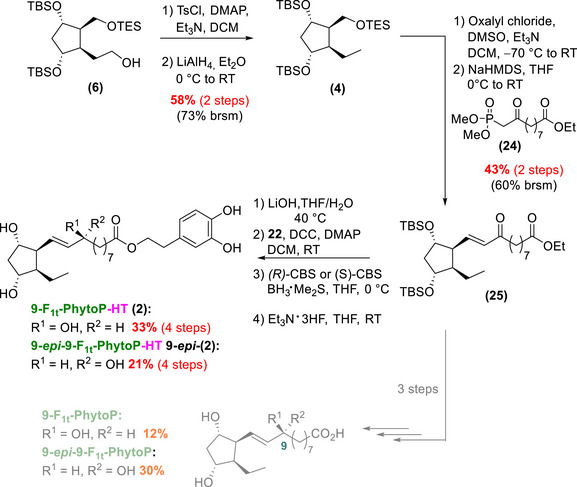
Final steps toward 9‐F_1t_‐PhytoP‐HT **(2)** and its C9‐epimer **9‐*epi*‐(2)** and access to the 9‐F_1t_‐PhytoP and its C9‐epimer (in grey) that will serve as analytical standards (see Supporting Information for more details on the synthesis of the 9‐F_1t_‐PhytoP and the 9‐*epi*‐9‐F_1t_‐PhytoP).

The synthesis of 9‐F_1t_‐PhytoP‐HT **(2)** and its C9‐epimer **9‐*epi*‐(2)** began with the second intermediate, alcohol **(6)** (Scheme 8).

Alcohol **(6)** was tosylated prior to LiAlH_4_ reduction,^[^ ^42^ ^]^ to access compound **(4)** with a moderate yield (58% over two steps). Due to the disappointing results with the phosphonium‐HT‐salt **(15)** in the 16‐series, we chose to use protected‐HT **(22)** to install the phenol‐scaffold later in the synthesis and thus performed the HWE olefination with ethyl ester phosphonate **(24)**.^[^ ^38^ ^]^ Thanks to the choice of the TES‐protecting group, a direct oxidation on **(4)** following the procedure by Rodriguez *et al.* ^[^ ^43^ ^]^ generated the aldehyde required for the HWE reaction, avoiding the need of an additional deprotection step. This strategy successfully produced enone **(25)** in a good yield (over two steps).

The synthesis was achieved with a four‐step sequence: saponification of the ethyl ester, coupling with protected‐HT **(22)**, CBS‐stereoselective reductions, and final deprotection of the silyl groups. These four steps yielded the corresponding 9‐F_1t_‐PhytoP‐HT **(2)** and its C9‐epimer, **9‐*epi*‐(2)**, in 33% and 21% yield, respectively.

Thus, our synthetic journey successfully provided access to four new HT‐ALA metabolites: the 16‐F_1t_‐PhytoP‐HT **(1)**, 16‐*epi*‐16‐F_1t_‐PhytoP‐HT **16‐*epi*‐(1)**, 9‐F_1t_‐PhytoP‐HT **(2)**, and 9‐*epi*‐9‐F_1t_‐PhytoP‐HT, **9‐*epi*‐(2)**. The syntheses were achieved in 20 steps, with very good yields per step, ranging from 73% to 82%.

It should be mentioned that for analytical purposes natural PhytoPs (nonlinked to HT) were also synthesized, such as the 9‐F_1t_‐PhytoP and its C9‐epimer from **(25)** (Scheme 8), and the 16‐F_1t_‐PhytoP and its C16‐epimer from **(21)** (Scheme 6) (see Supplementary information).

### 2.5 Investigation of PhytoP‐HT Occurrence in Oxidized HT‐ALA Lipophenols by UHPLC‐HRMS/MS

To investigate the potential formation of PhytoPs‐HT conjugates, in vitro oxidation experiments of HT‐ALA were performed. HT‐ALA was previously synthesized from compound **(22)**, as described in the Supplementary information. Two oxidative conditions were evaluated using the radical initiator V70 (2,2′‐azobis(4‐methoxy‐2,4‐dimethylvaleronitrile)), at 40 °C for 24 hours (Table 1, Entries 1 and 2). V70 is well known for its ability to generate stable radicals, which are essential for initiating the oxidation of PUFAs.^[^ ^44^ ^]^ Although these conditions represent an accelerated oxidative environment, V70 enables controlled oxidation conditions that mimic oxidative stress commonly observed in biological systems,^[^ ^45^ ^]^ thereby facilitating the formation of oxylipins.^[^ ^46^ ^]^ Its solubility in organic solvents and effectiveness at relatively low temperature also make it more practical than other azo‐based radical initiators.^[^ ^47^ ^]^

**Table 1:** Conditions employed for the in vitro oxidations of HT‐ALA and control samples.

Entries	Sample	Radical initiator	Solvent	Additives	Temperature, Time
1	HT‐ALA	V70	CH_3_CN	H_2_O	40 °C, 24 hours
2	HT‐ALA	V70	CH_3_CN	‐	40 °C, 24 hours
3^[^ ^a^ ^]^	HT‐ALA	‐	‐	‐	RT, 1 month
4	ALA, HT	V70	CH_3_CN	‐	40 °C, 24 hours

^[a]^ In these conditions, HT‐ALA was oxidized under natural conditions, UV light (window), and room temperature.

As a control, the oxidation of free ALA in the presence of HT was also carried out to determine whether the analytical profile differed when HT was covalently linked to ALA versus when ALA was not (Table 1, Entry 4). Additionally, an assay under natural conditions was conducted by exposing HT‐ALA to ambient light at room temperatures over the course of one month (Table 1, Entry 3).

Because the oxidation of a pure compound (i.e., HT‐ALA) generates many structurally diverse products, as well as regio‐ and diastereoisomers, the relative abundance of each individual species might be low. This makes selective enrichment critical to improve detectability, as this is well known in phytoprostane analysis.^[^ ^46^, ^48^ ^]^

Also, the analysis of enriched fractions (see Supplementary Information) that contain compounds sharing identical molecular formulas requires high‐throughput analytical approaches for adequate compound detection and annotation, among which ultra‐high performance liquid chromatography coupled with high‐resolution tandem mass spectrometry (UHPLC‐HRMS/MS) is currently one of the most effective techniques.^[^ ^46^ ^]^

**Figure 2:**
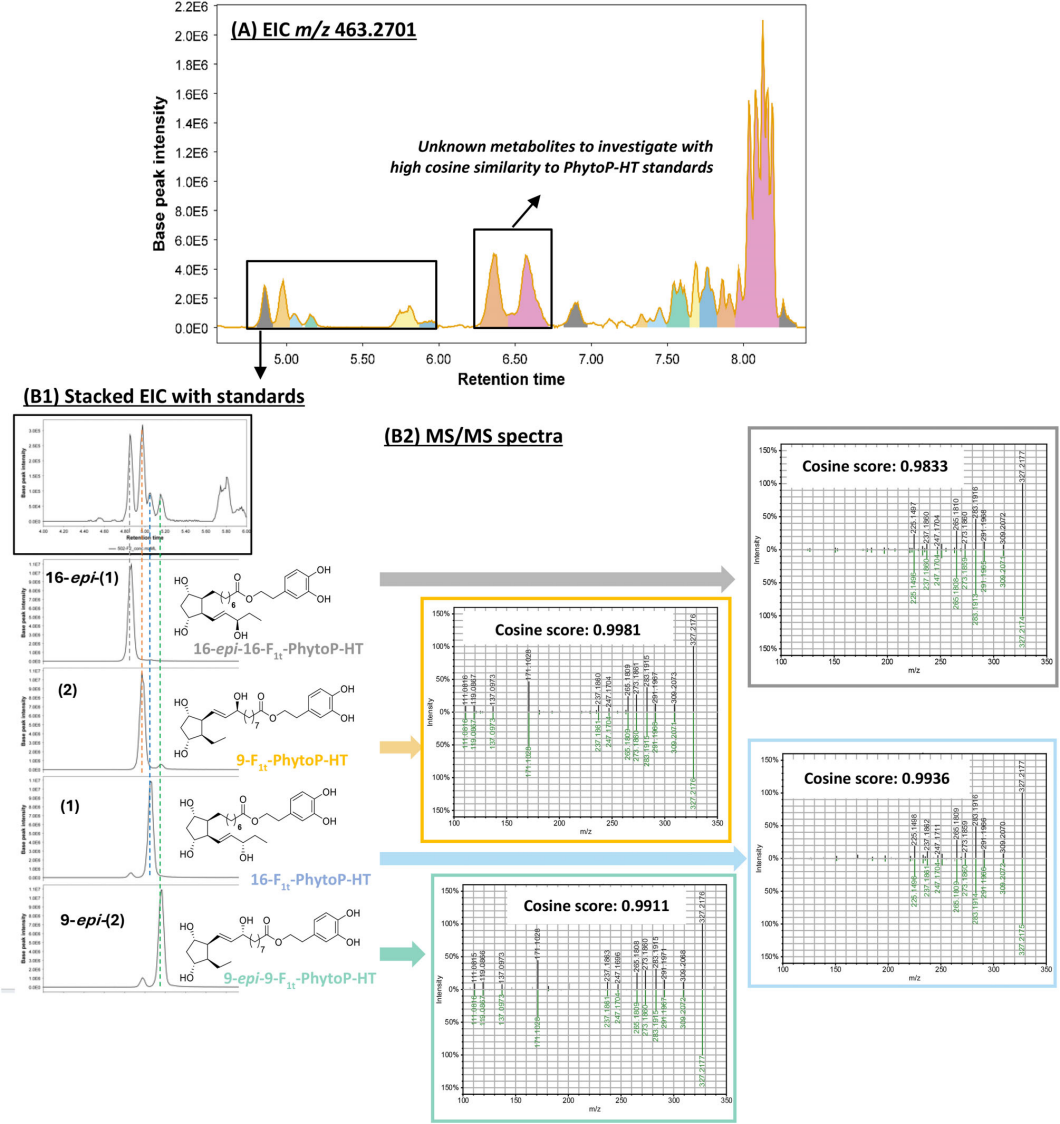
**UHPLC‐HRMS analysis of oxidized HT‐ALA using V70 initiator and H_2_O and comparison with individual PhytoPs‐HT standards: (A)** extracted ion chromatogram (EIC) of the oxidized sample, at *m/z* 463.2701 [M‐H]^−^ with an exact mass tolerance of ±5 ppm; **(B1)** comparison of retention times from EICs of the oxidized sample and of PhytoPs‐HT standard; **(B2)** comparison of MS/MS spectra extracted at 4.86, 4.98, 5.05, and 5.16 minutes from EIC (in black), with MS/MS spectra of PhytoPs‐HT standards (in green): Cosine scores for each unknown signal were calculated by comparing their MS/MS spectra with those of synthetic PhytoPs‐HT standards. All analyses were performed using UHPLC‐HRMS/MS (Orbitrap ID‐X Tribrid, Thermo Fisher Scientific) in data‐dependent acquisition (DDA) mode; the chromatographic profiles and MS/MS data were processed using MZmine version 4.7^[^ ^50^ ^]^ and the MS/MS data were visualized with the USI Resolver web service.^[^ ^51^ ^]^

In all oxidized samples, the extracted ion chromatograms (EICs) at the exact mass of the 9‐ and 16‐series PhytoPs‐HT molecular ions (i.e., [C_26_H_40_O_7_‐H]‐, *m/z* 463.2701) revealed multiple features (peaks) that could correspond to these PhytoPs‐HT conjugates, their regio‐ and diastereoisomers, or other interfering species. Figure 2 shows the EIC (Figure 2 **‐A and** 2 **‐B1**) and MS/MS spectra (Figure 2 **‐B2**) of HT‐ALA oxidation in the presence of V70 and water.

Thanks to the PhytoPs‐HT standards synthesized in this work, it was possible to confidently match both retention times and MS/MS spectra by calculating cosine similarity scores, enabling identification. High cosine values (close to 1) indicate strong spectral matches. Accordingly, features eluting at 4.86, 4.98, 5.05, 5.16, 6.37, and 6.58 minutes exhibited MS/MS spectra matching those of the standards, with cosine scores exceeding 0.977, and could be considered as potential PhytoPs‐HT. The efficient chromatographic separation achieved for each synthetic reference compound (Figure 2 **‐B1**) further allowed precise annotation of the features eluting at 4.86, 4.98, 5.05, and 5.16 minutes to compounds **(1)**, **16‐*epi*‐(1)**, **(2)**, and **9‐*epi*‐(2),** respectively (Figure 2 **‐B1)**. Based on the agreement in retention time and MS/MS fragmentation patterns (Figure 2 **‐B2**) with authentic standards, these identifications meet the criteria for Level 1 confidence according to current metabolomics reporting standards.^[^ ^49^ ^]^

Interestingly, the two features eluting at 6.37 and 6.58 minutes, labeled as “unknown” in Figure 2 **‐A**, showed MS/MS spectra with high similarity to those of PhytoPs‐HT standards but did not match their retention times. Moreover, they did not correspond to any entries in the GNPS public MS/MS libraries,^[^ ^52^, ^53^ ^]^ which include extensive oxylipin collections such as the NEO‐MS/MS library.^[^ ^46^ ^]^ These observations indicate that the features may represent distinct but structurally related entities, potentially corresponding to novel compounds.

**Figure 3:**
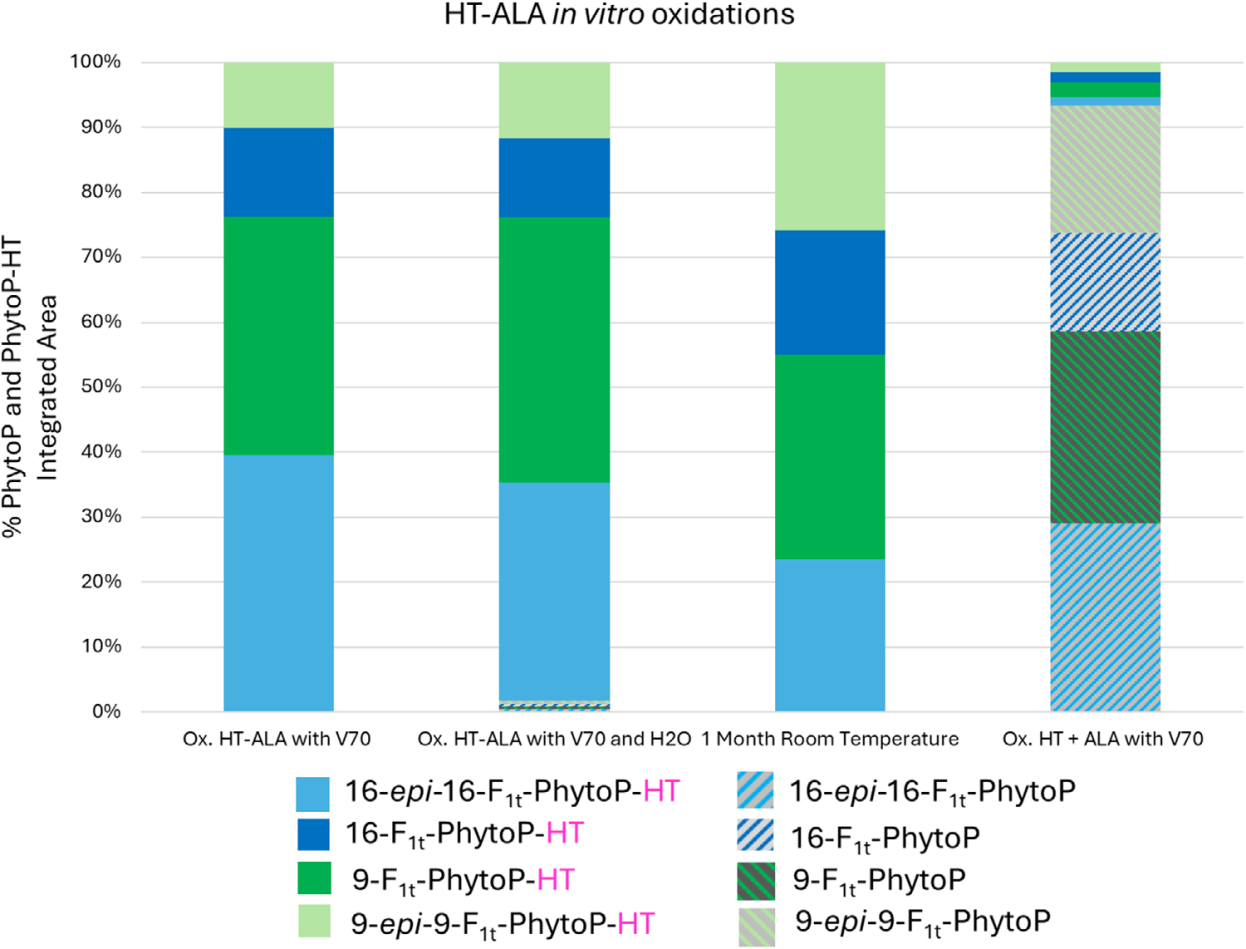
**Semi‐quantitative distribution of PhytoPs‐HT and PhytoPs obtained under the different oxidative conditions tested**. This semiquantitative assessment was based on the integration of the chromatographic peak areas corresponding to the molecular ions of each compound at their respective retention times, including both PhytoPs‐HT (*m/z* 463.2701, [C26H40O7–H]–) and unesterified PhytoPs (*m/z* 327.2177, [C18H32O5–H]–). Details of the data processing workflow and normalization procedures applied are provided in the Supplementary Data. Bars represent the relative integrated area (%) of the EICs corresponding to each identified compound, obtained by feature integration in MZmine version 4.7. For each sample, the area of each species was normalized to the total summed area of all eight detected compounds (four PhytoPs and four PhytoPs‐HT). Oxidation experiments were performed using HT‐ALA or ALA + HT with V70 or under ambient conditions (see Table 1, Entries 1–4).

On the other hand, the distribution of PhytoPs‐HT and PhytoPs formed under the tested oxidative conditions (Table 1), presented in Figure 3, highlights the relative proportions of both oxylipin types. These results demonstrate that PhytoPs‐HT are indeed formed during oxidation with V70, as well as during natural oxidation. A comparison between HT‐ALA and ALA + HT oxidations (Table 1 Entries 1 & 2 vs. Entry 4 and Figure 3) provides clear evidence for the conversion of HT‐ALA into PhytoPs‐HT, as well as the independent formation of PhytoPs from ALA, even in the presence of free HT. This suggests that the covalent linkage of HT to ALA is required for the formation of PhytoPs‐HT. Notably, oxidation with V70 appears to favor the formation of the 16‐F_1t_‐PhytoP‐HT epimer (**16‐*epi***‐**(1)**), whereas in the 9‐series, compound **(2)** is preferentially produced. This selective epimer formation is consistent with observations reported by previous studies on oxylipins.^[^ ^54^ ^]^ Finally, the oxidative profile observed after one month under natural light and ambient conditions differs markedly from those obtained under V70‐induced oxidation, suggesting that different oxidative pathways or radical species are involved or that the reaction kinetics are substantially altered under these conditions.

## 3 Conclusion

In this work we achieved the first total synthesis of four hydroxytyrosol‐phytoprostanes (PhytoPs‐HT) in 20 steps and in good overall yields. Thanks to the syntheses of PhytoPs‐HT, we were able to identify for the first time these compounds after in vitro oxidations of HT‐ALA, proving the formation of these metabolites of HT‐ALA lipophenols under oxidative conditions. We also highlighted different chromatographic profiles depending on the oxidative conditions regarding the series and stereoisomers of PhytoPs‐HT. This preliminary work, focusing on the discovery of oxidized lipophenol structures, highlights the potential of molecular networking to reveal the presence of unknown lipophenols distinct from PhytoPs‐HT and paves the way for future studies aimed at characterizing hydroxytyrosol‐derived compounds. Moreover, detecting such metabolites may serve as an indirect strategy for identifying HT‐related structures (or other lipophenols) across a wide range of natural and complex lipid matrices.

Beyond total synthesis and analysis, this work represents a first step toward the understanding of HT‐ALA metabolization and might be of great interest to the food industry for monitoring the oxidative status of HT‐ALA‐based food (e.g., olive oils). Continued development of this approach could significantly enhance the annotation of oxidized lipid products in complex biological and food samples. Ongoing studies aim to expand the molecular network for HT‐oxylipins and to further explore the pharmacological profiles of these metabolites, already present in the human diet, through in vitro and in vivo investigations.

## Supplementary Information

The authors have cited additional references within the Supporting Information.^[^ ^19^, ^33^, ^38^, ^39^, ^55^, ^56^ ^]^

## Conflict of Interest

The authors declare no conflict of interest.

## Supporting information



Supplementary Information

## Data Availability

The data that support the findings of this study are available in the supplementary material of this article.

## References

[1] C. Crauste , M. Rosell , T. Durand , J. Vercauteren , Biochimie 2016, 120, 62. 26209925 10.1016/j.biochi.2015.07.018

[2] D. Kahveci , M. Laguerre , P. Villeneuve , in Polar Lipids 2015, Elsevier, pp. 185.

[3] G. Williamson , C. Manach , Am. J. Clinicl Nutr. 2005, 81, 243S. 10.1093/ajcn/81.1.243S 15640487

[4] S. Quideau , D. Deffieux , C. Douat‐Casassus , L. Pouységu , Angew. Chem., Int. Ed. 2011, 50, 586. 10.1002/anie.201000044 21226137

[5] K. B. Pandey , S. I. Rizvi , Oxid. Med. Cell. Longev. 2009, 2, 270. 20716914 10.4161/oxim.2.5.9498 PMC2835915

[6] N. Siriwardhana , N. S. Kalupahana , N. Moustaid‐Moussa , in Adv. Food Nutr. Res. 2012, Elsevier, 65, pp. 211. 22361189 10.1016/B978-0-12-416003-3.00013-5

[7] C. Crauste , C. Vigor , P. Brabet , M. Picq , M. Lagarde , C. Hamel , T. Durand , J. Vercauteren , Eur. J. Org. Chem. 2014, 2014, 4548.

[8] M. Vincent , J. Lehoux , C. Desmarty , E. Moine , P. Legrand , C. Dorandeu , L. Simon , T. Durand , P. Brabet , C. Crauste , S. Begu , Int. J. Pharm. 2024, 651, 12374. 10.1016/j.ijpharm.2023.123740 38145781

[9] C. Benincasa , C. La Torre , A. Fazio , E. Perri , M. C. Caroleo , P. Plastina , E. Cione , Antioxidants 2021, 10, 1051. 34209968 10.3390/antiox10071051 PMC8300722

[10] P. Plastina , C. Benincasa , E. Perri , A. Fazio , G. Augimeri , M. Poland , R. Witkamp , J. Meijerink , Food Chem. 2019, 279, 105. 30611468 10.1016/j.foodchem.2018.12.007

[11] F.‐W. Yin , X.‐P. Hu , D.‐Y. Zhou , X.‐C. Ma , X.‐G. Tian , X.‐K. Huo , K. Rakariyatham , F. Shahidi , B.‐W. Zhu , Food Funct. 2018, 9, 3610. 29968877 10.1039/c8fo00788h

[12] F. Yin , X. Wang , Y. Hu , H. Xie , X. Liu , L. Qin , J. Zhang , D. Zhou , F. Shahidi , J. Agric. Food Chem. 2020, 68, 1248. 31927921 10.1021/acs.jafc.9b05112

[13] C. Alemán‐Jiménez , R. Domínguez‐Perles , J. I. Gallego‐Gómez , A. Simonelli‐Muñoz , E. Moine , T. Durand , C. Crauste , F. Ferreres , Á. Gil‐Izquierdo , S. Medina , J. Agric. Food Chem. 2021, 69, 14165. 34797062 10.1021/acs.jafc.1c05373

[14] G. Cefarelli , B. D'Abrosca , A. Fiorentino , A. Izzo , P. Monaco , J. Agric. Food Chem. 2005, 53, 3525. 15853397 10.1021/jf047838g

[15] R. A. Myers , E. Fuller , W. Yang , J. Agric. Food Chem. 2013, 61, 11484. 24251649 10.1021/jf403620f

[16] S. Medina , R. Domínguez‐Perles , D. Auñón , E. Moine , T. Durand , C. Crauste , F. Ferreres , Á. Gil‐Izquierdo , J. Agric. Food Chem. 2020, 68, 7789. 32603105 10.1021/acs.jafc.0c01938

[17] C. Benincasa , A. Cersosimo , E. Perri , R. Nicoletti , C. La Torre , A. Fazio , E. Cione , D. M. Abrego‐Guandique , M. C. Caroleo , P. Plastina , ACS Food Sci. Technol. 2024, 4, 1570.

[18] S. Liu , Y. Zhu , N. Liu , D. Fan , M. Wang , Y. Zhao , J. Agric. Food Chem. 2021, 69, 1057. 33440930 10.1021/acs.jafc.0c07273

[19] Y. Y. Lee , C. Crauste , H. Wang , H. H. Leung , J. Vercauteren , J.‐M. Galano , C. Oger , T. Durand , J. M.‐F. Wan , J. C.‐Y. Lee , Chem. Res. Toxicol. 2016, 29, 1689. 27588434 10.1021/acs.chemrestox.6b00214

[20] S. Medina , D. Auñón , J. Lehoux , T. Durand , C. Crauste , Á. Gil‐Izquierdo , Microchem. J. 2022, 181, 107656.

[21] T. A. Davis , L. Gao , H. Yin , J. D. Morrow , N. A. Porter , J. Am. Chem. Soc. 2006, 128, 14897. 17105300 10.1021/ja064399o

[22] R. Imbusch , M. J. Mueller , Biol. Med. 2000, 28, 720. 10.1016/s0891-5849(00)00154-4 10754267

[23] S. Parchmann , M. J. Mueller , J. Biol. Chem. 1998, 273, 32650. 9830005 10.1074/jbc.273.49.32650

[24] I. Thoma , M. Krischke , C. Loeffler , M. J. Mueller , Chem. Phys. Lipids 2004, 128, 135. 15037159 10.1016/j.chemphyslip.2003.10.002

[25] T. Durand , V. Bultel‐Poncé , A. Guy , S. Berger , M. J. Mueller , J.‐M. Galano , Lipids 2009, 44, 875. 19789901 10.1007/s11745-009-3351-1

[26] C. Oger , T. Pavlíčková , V. Bultel‐Poncé , A. Guy , J.‐M. Galano , U. Jahn , T. Durand , Prog. Lipid Res. 2024, 96, 101301. 39284419 10.1016/j.plipres.2024.101301

[27] D. F. Taber , J. D. Morrow , L. J. Roberts , Prostaglandins 1997, 53, 63. 9112285 10.1016/s0090-6980(97)00005-1

[28] K. S. Leung , C. Oger , A. Guy , V. Bultel‐Poncé , C. Vigor , T. Durand , A. Gil‐Izquierdo , S. Medina , J.‐M. Galano , J. C.‐Y. Lee , Advances in Botanical Research, Chapter Eleven ‐ Alpha‐linolenic acid, phytoprostanes and phytofurans in plant, algae and food, Elsevier, 2022, Vol. 101, pp. 437–468.

[29] S. M. Sánchez , R. Domínguez‐Perles , S. Montoro‐García , J. A. Gabaldón , A. Guy , T. Durand , C. Oger , F. Ferreres , A. Gil‐Izquierdo , Food Funct. 2020, 11, 5166. 32432610 10.1039/d0fo00976h

[30] L. Minghetti , R. Salvi , M. Lavinia Salvatori , M. Antonietta Ajmone‐Cat , C. De Nuccio , S. Visentin , V. Bultel‐Poncé , C. Oger , A. Guy , J.‐M. Galano , A. Greco , A. Bernardo , T. Durand , Free Radical Biol. Med. 2014, 73, 41. 24794409 10.1016/j.freeradbiomed.2014.04.025

[31] R. Domínguez‐Perles , Á. Abellán , D. León , F. Ferreres , A. Guy , C. Oger , J. M. Galano , T. Durand , Á. Gil‐Izquierdo , Food Res. Int. 2018, 107, 619. 29580528 10.1016/j.foodres.2018.03.013

[32] J. Collado‐González , C. Grosso , P. Valentão , P. B. Andrade , F. Ferreres , T. Durand , A. Guy , J.‐M. Galano , A. Torrecillas , Á. Gil‐Izquierdo , Food Chem. 2017, 235, 298. 28554640 10.1016/j.foodchem.2017.04.171

[33] C. Oger , Y. Brinkmann , S. Bouazzaoui , T. Durand , J.‐M. Galano , Org. Lett. 2008, 10, 5087. 18844366 10.1021/ol802104z

[34] K. C. Nicolaou , P. S. Baran , Y.‐L. Zhong , K. Sugita , J. Am. Chem. Soc. 2002, 124, 2212. 11878975 10.1021/ja012124x

[35] V. K. Yadav , K. K. Kapoor , Tetrahedron 1995, 51, 8573.

[36] T. Suzuki , K. Morita , M. Tsuchida , K. Hiroi , Org. Lett. 2002, 4, 2361. 12098247 10.1021/ol026091h

[37] C. Oger , Z. Marton , Y. Brinkmann , V. Bultel‐Poncé , T. Durand , M. Graber , J.‐M. Galano , J. Org. Chem. 2010, 75, 1892. 20187621 10.1021/jo902541c

[38] S. El Fangour , A. Guy , V. Despres , J.‐P. Vidal , J.‐C. Rossi , T. Durand , J. Org. Chem. 2004, 69, 2498. 15049650 10.1021/jo035638i

[39] H. I. Duynstee , M. C. de Koning , H. Ovaa , G. A. van der Marel , J. H. van Boom , Eur. J. Org. Chem. 1999, 1999, 2623.

[40] E. J. Corey , R. K. Bakshi , S. Shibata , J. Am. Chem. Soc. 1987, 109, 5551.

[41] I. Chataigner , J. Lebreton , D. Durand , A. Guingant , J. Villiéras , Tetrahedron Lett. 1998, 39, 1759.

[42] S. Krishnamurthy , H. C. Brown , J. Org. Chem. 1976, 41, 3064.

[43] A. Rodríguez , M. Nomen , B. W. Spur , J. J. Godfroid , Tetrahedron Lett. 1999, 40, 5161.

[44] H. Yin , L. Xu , N. A. Porter , Chem. Rev. 2011, 111, 5944. 21861450 10.1021/cr200084z

[45] C. L. Rector , “New Insights into the secondary oxidation products of polyunsaturated fatty esters” Faculty of the Graduate School of Vanderbilt University, 2017.

[46] A. Elloumi , L. Mas‐Normand , J. Bride , G. Reversat , V. Bultel‐Poncé , A. Guy , C. Oger , M. Demion , J.‐Y. L. Guennec , T. Durand , C. Vigor , Á. Sánchez‐Illana , J.‐M. Galano , Sci. Data 2024, 11, 193. 38351090 10.1038/s41597-024-03034-4 PMC10864323

[47] Y. Kita , K. Gotanda , K. Murata , M. Suemura , A. Sano , T. Yamaguchi , M. Oka , M. Matsugi , Org. Process Res. Dev. 1998, 2, 250.

[48] N. H. Schebb , N. Kampschulte , G. Hagn , K. Plitzko , S. W. Meckelmann , S. Ghosh , R. Joshi , J. Kuligowski , D. Vuckovic , M. T. Botana , Á. Sánchez‐Illana , F. Zandkarimi , A. Das , J. Yang , L. Schmidt , A. Checa , H. M. Roche , A. M. Armando , M. L. Edin , F. B. Lih , J. J. Aristizabal‐Henao , S. Miyamoto , F. Giuffrida , A. Moussaieff , R. Domingues , M. Rothe , C. Hinz , U. S. Das , K. M. Rund , A. Y. Taha , R. K. Hofstetter , M. Werner , O. Werz , A. S. Kahnt , J. Bertrand‐Michel , P. Le Faouder , R. Gurke , D. Thomas , F. Torta , I. Milic , I. H. K. Dias , C. M. Spickett , D. Biagini , T. Lomonaco , H. Idborg , J.‐Y. Liu , M. Fedorova , D. A. Ford , A. Barden , T. A. Mori , P. D. Kennedy , K. Maxey , J. Ivanisevic , H. Gallart‐Ayala , C. Gladine , M. Wenk , J.‐M. Galano , T. Durand , K. D. Stark , C. Barbas , U. Garscha , S. L. Gelhaus , U. Ceglarek , N. Flamand , J. L. Griffin , R. Ahrends , M. Arita , D. C. Zeldin , F. J. Schopfer , O. Quehenberger , R. Julian , A. Nicolaou , I. A. Blair , M. P. Murphy , B. D. Hammock , B. Freeman , G. Liebisch , C. N. Serhan , H. C. Köfeler , P.‐J. Jakobsson , D. Steinhilber , M. H. Gelb , M. Holčapek , R. Andrew , M. Giera , G. A. FitzGerald , R. C. Murphy , J. W. Newman , E. A. Dennis , K. Ekroos , G. L. Milne , M. A. Gijón , H. W. Vesper , C. E. Wheelock , V. B. O'Donnell , Sci. Signal. 2025, 18, eadw1245. 40392938 10.1126/scisignal.adw1245 PMC12330888

[49] W. Bittremieux , C. Chen , P. C. Dorrestein , E. L. Schymanski , T. Schulze , S. Neumann , R. Meier , S. Rogers , M. Wang , bioRxiv 2020, 2020 .05.09.086066, 10.1101/2020.05.09.086066.

[50] R. Schmid , S. Heuckeroth , A. Korf , A. Smirnov , O. Myers , T. S. Dyrlund , R. Bushuiev , K. J. Murray , N. Hoffmann , M. Lu , A. Sarvepalli , Z. Zhang , M. Fleischauer , K. Dührkop , M. Wesner , S. J. Hoogstra , E. Rudt , O. Mokshyna , C. Brungs , K. Ponomarov , L. Mutabdžija , T. Damiani , C. J. Pudney , M. Earll , P. O. Helmer , T. R. Fallon , T. Schulze , A. Rivas‐Ubach , A. Bilbao , H. Richter , L.‐F. Nothias , M. Wang , M. Orešič , J.‐K. Weng , S. Böcker , A. Jeibmann , H. Hayen , U. Karst , P. C. Dorrestein , D. Petras , X. Du , T. Pluskal , Nat. Biotechnol. 2023, 41, 447. 36859716 10.1038/s41587-023-01690-2 PMC10496610

[51] L. W. Sumner , A. Amberg , D. Barrett , M. H. Beale , R. Beger , C. A. Daykin , T. W.‐M. Fan , O. Fiehn , R. Goodacre , J. L. Griffin , T. Hankemeier , N. Hardy , J. Harnly , R. Higashi , J. Kopka , A. N. Lane , J. C. Lindon , P. Marriott , A. W. Nicholls , M. D. Reily , J. J. Thaden , M. R. Viant , Metabolomics 2007, 3, 211. 24039616 10.1007/s11306-007-0082-2 PMC3772505

[52] M. Wang , J. J. Carver , V. V. Phelan , L. M. Sanchez , N. Garg , Y. Peng , D. D. Nguyen , J. Watrous , C. A. Kapono , T. Luzzatto‐Knaan , C. Porto , A. Bouslimani , A. V. Melnik , M. J. Meehan , W.‐T. Liu , M. Crüsemann , P. D. Boudreau , E. Esquenazi , M. Sandoval‐Calderón , R. D. Kersten , L. A. Pace , R. A. Quinn , K. R. Duncan , C.‐C. Hsu , D. J. Floros , R. G. Gavilan , K. Kleigrewe , T. Northen , R. J. Dutton , D. Parrot , E. E. Carlson , B. Aigle , C. F. Michelsen , L. Jelsbak , C. Sohlenkamp , P. Pevzner , A. Edlund , J. McLean , J. Piel , B. T. Murphy , L. Gerwick , C.‐C. Liaw , Y.‐L. Yang , H.‐U. Humpf , M. Maansson , R. A. Keyzers , A. C. Sims , A. R. Johnson , A. M. Sidebottom , B. E. Sedio , A. Klitgaard , C. B. Larson , C. A. Boya P , D. Torres‐Mendoza , D. J. Gonzalez , D. B. Silva , L. M. Marques , D. P. Demarque , E. Pociute , E. C. O'Neill , E. Briand , E. J. N. Helfrich , E. A. Granatosky , E. Glukhov , F. Ryffel , H. Houson , H. Mohimani , J. J. Kharbush , Y. Zeng , J. A. Vorholt , K. L. Kurita , P. Charusanti , K. L. McPhail , K. F. Nielsen , L. Vuong , M. Elfeki , M. F. Traxler , N. Engene , N. Koyama , O. B. Vining , R. Baric , R. R. Silva , S. J. Mascuch , S. Tomasi , S. Jenkins , V. Macherla , T. Hoffman , V. Agarwal , P. G. Williams , J. Dai , R. Neupane , J. Gurr , A. M. C. Rodríguez , A. Lamsa , C. Zhang , K. Dorrestein , B. M. Duggan , J. Almaliti , P.‐M. Allard , P. Phapale , L.‐F. Nothias , T. Alexandrov , M. Litaudon , J.‐L. Wolfender , J. E. Kyle , T. O. Metz , T. Peryea , D.‐T. Nguyen , D. VanLeer , P. Shinn , A. Jadhav , R. Müller , K. M. Waters , W. Shi , X. Liu , L. Zhang , R. Knight , P. R. Jensen , B. Ø. Palsson , K. Pogliano , R. G. Linington , M. Gutiérrez , N. P. Lopes , W. H. Gerwick , B. S. Moore , P. C. Dorrestein , N. Bandeira , Nat. Biotechnol. 2016, 34, 828. 27504778 10.1038/nbt.3597 PMC5321674

[53] Link to GNPS: https://library.gnps2.org.

[54] H. Yin , C. M. Havrilla , J. D. Morrow , N. A. Porter , J. Am. Chem. Soc. 2002, 124, 7745. 12083928 10.1021/ja0201092

[55] M. Frigerio , M. Santagostino , S. Sputore , J. Org. Chem. 1999, 64, 4537.

[56] N. Cabedo , I. Andreu , M. C. Ramírez de Arellano , A. Chagraoui , A. Serrano , A. Bermejo , P. Protais , D. Cortes , J. Med. Chem. 2001, 44, 1794. 11356113 10.1021/jm001128u

