# Supplementary material for: Total Synthesis and Analysis of Phenolic Phytoprostanes, Oxidized Derivatives of Lipophenols
Source: Chemistry. 2025 Oct 15;31(62):e02206. doi: 10.1002/chem.202502206 (PMC12598386; doi:10.1002/chem.202502206)
Supplement: Supplementary file 1 — Supplementary Information [file CHEM-31-e02206-s001.docx]

Total Synthesis and Analysis of Phenolic Phytoprostanes, Oxidized Derivatives of Lipophenols

Jordan Lehoux,^[a]^ Ángel Sánchez-Illana,^[b]^ Pablo Miralles,^[c]^ Thierry Durand^[a]^, Céline Crauste*^[a]^ and Camille Oger*^[a]^

Summary

[General Methods. 2](#_Toc209788819)

[Experimental procedures 4](#_Toc209788820)

[1. Preparation of reagents, common intermediates, phosphonates and phosphonium: 4](#_Toc209788821)

[i. Preparation of 1-hydroxy-1-oxo-1l5-benzo[d][1,2]iodaoxol-3(1H)-one (IBX) (**S1**) 4](#_Toc209788822)

[ii. Preparation of compound iridium catalyst (**S2**) 4](#_Toc209788823)

[iii. Preparation of 2-(3,4-bis((tert-butyldimethylsilyl)oxy)phenyl)ethan-1-ol (**22**) 4](#_Toc209788824)

[iv. Preparation of (6-(3,4-bis((tert-butyldimethylsilyl)oxy)phenethoxy)-6-oxohexyl)triphenyl phosphonium bromide **(15)** 5](#_Toc209788825)

[v. Preparation of ethyl 10-(dimethoxyphosphoryl)-9-oxodecanoate (**24**) 6](#_Toc209788826)

[vi. Preparation of (4a*S*,5*S*,7*R*,7a*S*)-5,7-bis((tert-butyldimethylsilyl)oxy)octahydrocyclopenta [c]pyran-3-ol (**5**) 6](#_Toc209788827)

[vii. Preparation of 2-((1*R*,2*R*,3*S*,5*R*)-3,5-bis((tert-butyldimethylsilyl)oxy)-2-(((triethylsilyl)oxy)methyl) cyclopentyl)ethan-1-ol (**6**) 13](#_Toc209788828)

[2. Towards the synthesis of the 16-F_1t_-PhytoP-HT and its C16-epimer 14](#_Toc209788829)

[i. Preparation of methyl (*Z*)-8-((1*S*,2*S*,3*R*,5*S*)-3,5-bis((tert-butyldimethylsilyl)oxy)-2-(hydroxymethyl)cyclopentyl)oct-6-enoate (**19**) 14](#_Toc209788830)

[ii. Preparation of methyl 8-((1*S*,2*R*,3*R*,5*S*)-3,5-bis((tert-butyldimethylsilyl)oxy)-2-((*E*)-3-oxopent-1-en-1-yl)cyclopentyl)octanoate (**21**) 15](#_Toc209788831)

[iii. Preparation of 3,4-bis((tert-butyldimethylsilyl)oxy)phenethyl 8-((1*S*,2*R*,3*R*,5*S*)-3,5-bis((tert-butyldimethylsilyl)oxy)-2-((*E*)-3-oxopent-1-en-1-yl)cyclopentyl)octanoate (**23**) 16](#_Toc209788832)

[iv. Preparation of 3,4-dihydroxyphenethyl 8-((1*S*,2*R*,3*R*,5*S*)-3,5-dihydroxy-2-((*S*,*E*)-3-hydroxypent-1-en-1-yl)cyclopentyl)octanoate – 16-F_1t_-PhytoP-HT (**1**) 17](#_Toc209788833)

[v. Preparation of 3,4-dihydroxyphenethyl 8-((1*S*,2*R*,3*R*,5*S*)-3,5-dihydroxy-2-((*R*,*E*)-3-hydroxypent-1-en-1-yl)cyclopentyl)octanoate – 16-*epi*-16-F_1t_-PhytoP-HT (***16-epi-*1**) 18](#_Toc209788834)

[3. Towards the synthesis of the 16-F_1t_-PhytoP and its C16-epimer 19](#_Toc209788835)

[i. Preparation of 8-((1*S*,2*R*,3*R*,5*S*)-3,5-dihydroxy-2-((*S*,*E*)-3-hydroxypent-1-en-1-yl)cyclopentyl) octanoic acid – 16-F_1t_-PhytoP (**S13**) 19](#_Toc209788836)

[ii. Preparation of 8-((1*S*,2*R*,3*R*,5*S*)-3,5-dihydroxy-2-((*R*,*E*)-3-hydroxypent-1-en-1-yl)cyclopentyl)octanoic acid – 16-epi-16-F1t-PhytoP (**16-*epi*-S13**) 19](#_Toc209788837)

[4. Towards the synthesis of the 9-F_1t_-PhytoP-HT and its C9-epimer 20](#_Toc209788838)

[i. Preparation of (((1*S*,3*R*,4*R*,5*R*)-4-ethyl-5-(((triethylsilyl)oxy)methyl)cyclopentane-1,3-diyl)bis(oxy))bis(tert-butyldimethylsilane) (**4**) 20](#_Toc209788839)

[ii. Preparation of ethyl (*E*)-11-((1*S*,2*R*,3*R*,5*S*)-3,5-bis((tert-butyldimethylsilyl)oxy)-2-ethylcyclopentyl)-9-oxoundec-10-enoate (**25**) 21](#_Toc209788840)

[iii. Preparation of 3,4-dihydroxyphenethyl (*S*,*E*)-11-((1*S*,2*R*,3*R*,5*S*)-2-ethyl-3,5-dihydroxy cyclopentyl)-9-hydroxyundec-10-enoate – 9-F_1t_-PhytoP-HT (**2**) 22](#_Toc209788841)

[iv. Preparation of 3,4-dihydroxyphenethyl (*R*,*E*)-11-((1*S*,2*R*,3*R*,5*S*)-2-ethyl-3,5-dihydroxycyclopentyl)-9-hydroxyundec-10-enoate (**9*-epi-*2**) 24](#_Toc209788842)

[5. Towards the synthesis of the 9-F_1t_-PhytoP and its C9-epimer 24](#_Toc209788843)

[i. Preparation of (*S*,*E*)-11-((1*S*,2*R*,3*R*,5*S*)-2-ethyl-3,5-dihydroxycyclopentyl)-9-hydroxyundec-10-enoic acid – 9-F_1t_-PhytoP (**S19**) 25](#_Toc209788844)

[ii. Preparation of (*R*,*E*)-11-((1*S*,2*R*,3*R*,5*S*)-2-ethyl-3,5-dihydroxycyclopentyl)-9-hydroxyundec-10-enoic acid – 9-*epi*-9-PhytoP (**9-*epi*-S19**) 25](#_Toc209788845)

[6. Determination of the absolute configuration of the stereocenters 26](#_Toc209788846)

[i. Derivatization as mandelates and NMR study 26](#_Toc209788847)

[ii. HPLC analysis and comparison 28](#_Toc209788848)

[Analytical study 31](#_Toc209788849)

[1. Preparation of 3,4-dihydroxyphenethyl (9*Z*,12*Z*,15*Z*)-octadeca-9,12,15-trienoate (HT-ALA) (**S23**) 31](#_Toc209788850)

[2. General procedure for oxidations with V70 31](#_Toc209788851)

[3. UHPLC-HRMS/MS analysis 31](#_Toc209788852)

[4. Distribution of PhytoPs-HT and PhytoPs in oxidized sample 33](#_Toc209788853)

[5. MS/MS spectra at m/z 463.2701 and cosine score 34](#_Toc209788854)

[NMR Spectra and HPLC chromatograms 37](#_Toc209788855)

# General Methods.

All solvents were anhydrous reagents from commercial sources. Unless otherwise noted, all chemicals and reagents were obtained commercially and used without purification. Unless otherwise noted, the reactions were performed in dry glassware under N_2_ atmosphere. The reactions were monitored using TLC on plates that were pre-coated with silica gel 60 with fluorescent indicator 254 nm (Macherey-Nagel). The reaction components were visualized using a 254 nm UV lamp, stained with acidic *p*-anisaldehyde solution followed by gentle heating. Purifications of the synthesized compounds were performed by column chromatography on silica gel 40–63 µm Macherey-Nagel.

Optical rotations were recorded on JASCO P2000-series apparatus are given in 10^-1^ deg.cm².g^-1^. Infrared spectra were taken on a Spectrum one or three (Perkin Elmer) as neat samples using an ATR device and absorptions are given in wave numbers (cm^–1^). High resolution mass spectrometry (HRMS) spectra were measured using electrospray ionization (ESI) on a Q-Tof micro spectrometer (resolution 100000, Waters) or on mass spectrometers Synapt G2-S (Waters) or Orbitrap ID-X (Waters). Data were obtained by positive or negative electrospray ionization methods between 100 and 1500 Da by direct introduction. NMR spectra were recorded on Bruker AMX400 or Bruker Avance III 500 MHz or 600MHz (cryoprobs) spectrometers in CDCl_3_ or CD_3_OD. ^1^H NMR chemical shifts were provided in ppm using internal reference at δ = 7.26 ppm for CDCl_3_ and δ = 3.31 ppm for CD_3_OD and are reported as follow: chemical shift in ppm [multiplicity, coupling constant(s) *J* in Hz, relative integral, attribution]. The multiplicities are defined as follow: br = broad, m = multiplet, s = singlet, d = doublet, t = triplet, q = quadruplet, quint = quintuplet or combinations thereof. ^13^C NMR chemical shifts were referenced against the residual solvent central peak (δ = 77.16 ppm for CDCl_3_ and δ = 49.0 ppm for CD_3_OD). The connectivity was determined by ^1^H-^1^H COSY experiments; carbons were assigned according to HSQC and HMBC NMR experiments. For the peak assignments, the following abbreviations were used: Ar = aromatic, CH=CH = aliphatic alkene, *t*-Bu = *tert*-butyl. The carbon and hydrogen numbering refers to the molecule drawn on the NMR spectra and not on the IUPAC numbering. Diastereoisomeric excesses were determined using HPLC Shimadzu Prominence UFLC with a Shim-Pack GIS Shimadzu column (5 µm C18 3.0 x 100 mm) using a 0.5 mL/min flow of solvent A (H2O +0.1% formic acid) and solvent B (MeCN + 0.1% formic acid) and the following gradient : 0.0 min, 22% B; 1 min, 22% B; 10 min, 28% B; 11 min, 100% B; 13 min, 100% B; 13.1 min, 22% B; 18 min, 22% B. UV detection from 190 to 800 nM.

# Experimental procedures

## Preparation of reagents, common intermediates, phosphonates and phosphonium:

### Preparation of 1-hydroxy-1-oxo-1l5-benzo[d][1,2]iodaoxol-3(1H)-one (IBX) (**S1**)

Oxone (192 g, 0.62 mol, 3.1 equiv.) was suspended in distilled water (650 mL) and heated at 50°C under mechanical stirring until complete dissolution. 2-iodobenzoic acid (50 g, 0.20 mmol, 1 equiv.) was then added portion wise over 40 minutes under slow agitation (130 rpm) and the mixture was stirred at 68°C in the dark for 1 hour followed by 3 hours at 210 rpm. The mixture was then cooled to 5°C maximum with an ice bath and maintained at this temperature for 1 hour. The precipitate was then filtered and washed with 6 x 100 mL cold H_2_O followed by 2 x 100 mL acetone to yield the desired product **S1** (48.8 g, 0.17 mmol, 87%) as a white solid. Note that the product contains approximately 6% of starting material (estimated by ^1^H NMR integration).

^1^H NMR (400 MHz, DMSO-*d*_6_)**:** δ 8.15 (d, ^3^*J*_H,H_ = 7.9 Hz, 1H, *H_Ar_*), 8.06 – 7.97 (m, 2H, *H_Ar_*), 7.84 (t, ^3^*J*_H,H_ = 7.2 Hz, 1H, *H_Ar_*). In analogy to Frigerio *et al*. *J.* *Org. Chem*. **1999**, 64, 4537–4538.

### Preparation of compound iridium catalyst (**S2**)

To a solution of [Cp*IrCl_2_]_2_ (100 mg, 0.13 mmol, 1 equiv.) and 2,2-diphenyl glycinol (53 mg, 0.25 mmol, 2 equiv.) in CH_2_Cl_2_ (2.5 mL) was added a solution of KOH (200 mg, 3.50 mmol, 28 equiv.) in distilled water (2.5 mL). The mixture was stirred for 30 minutes at room temperature, then the layers were separated and the aqueous one extracted with CH_2_Cl_2_ (5 mL). The combined organic layers were dried over CaH_2_ and filtered. The evaporation of the solvent afforded **S2** (100 mg, 75%) as a red solid which is used without further purification.

### Preparation of 2-(3,4-bis((tert-butyldimethylsilyl)oxy)phenyl)ethan-1-ol (**22**)

H_2_SO_4_ (24 µL) was added to a solution of 3,4-dihydroxyphenylacetic acid (DOPAC) (1.5 g, 8.93 mmol, 1 equiv.) in MeOH (150 mL) at room temperature and the mixture was stirred at reflux for 2 hours. The solvent was then mostly evaporated, and the crude diluted with 100 mL EtOAc. The organic phase was washed with a saturated NaHCO_3_ solution (3 x 100 mL), brine (100 mL), dried over MgSO_4_ and the solvent was removed under reduced pressure. The ester **S3** was obtained (1.5 g, 8.23 mmol, 92%) as a colourless oil and engaged without further purification.

^1^H NMR (400 MHz, CDCl_3_): δ 6.80 – 6.75 (m, 2H, *H_Ar_*), 6.68 (dd, ^3^*J*_H,H_ = 8.1 Hz, ^4^*J*_H,H_ = 1.9 Hz, 1H, *H_Ar_*), 5.52 (br, 2H, O*H*), 3.71 (s, 3H, OC*H_3_*), 3.52 (s, 2H, C*H_2_*). In analogy to Cabedo *et al*. *J. Med. Chem.* **2001**, *44*, 1794–1801.

Imidazole (2.24 g, 32.9 mmol, 4 equiv.) and TBSCl (3.7 g, 24.7 mmol, 3 equiv.) were successively added to a solution of the ester **S3** (1.5 g, 8.2 mmol, 1 equiv.) in 40 mL anhydrous CH_2_Cl_2_ and the mixture was stirred overnight at room temperature. Then, saturated NH_4_Cl was added (100 mL) and the crude was extracted by CH_2_Cl_2_ (2 x 100 mL). The combined organic layers were washed with water (100 mL), brine (2x 100 mL), dried over MgSO_4_, filtered and the solvents removed under reduced pressure. The crude ester **S4** was directly engaged in next step without further purification (Note the presence of silanols).

LiAlH_4_ (1M in THF, 9 mL, 9 mmol, 1.1 equiv.) was added dropwise at 0°C to a solution of **S4** (8.2 mmol, 1 equiv.) in 250 mL THF and the mixture was stirred at 0°C for 3 hours. The reaction was then quenched by the dropwise addition of MeOH (10 mL, exothermic) and the crude was stirred vigorously for 2 hours with a Rochelle salt solution (1M, 200 mL). The precipitate was filtered on Celite® and rinsed with Et_2_O (100 mL). The resulting phases were separated, the aqueous one extracted with Et_2_O (2 x 100 mL), and the combined organic layers were washed with brine, dried over MgSO_4_, and concentrated under reduced pressure. The crude was then purified by silica gel column chromatography with pentane/EtOAc: 90/10 as eluent to afford the alcohol **22** (2.34 g, 6.11 mmol, 75% over 2 steps) as a colourless oil.

^1^H NMR (400 MHz, CDCl_3_): δ 6.76 (d, ^3^*J*_H,H_ = 8.0 Hz, 1H, *H_Ar_*), 6.69 (d, ^4^*J*_H,H_ = 2.1 Hz, 1H, *H_Ar_*), 6.65 (dd, ^3^*J*_H,H_ = 8.1 Hz, ^4^*J*_H,H_ = 2.2 Hz, 1H, *H_Ar_*), 3.79 (q, ^3^*J*_H,H_ = 6.4 Hz, 2H, C*H_2_*-OH), 2.74 (t, ^3^*J*_H,H_ = 6.5 Hz, 2H, C*H_2_*-CH_2_-OH), 1.35 (t, ^3^*J*_H,H_ = 6.1 Hz, 1H, O*H*), 0.98 (s, 9H, C*H_3_* *t*-Bu TBS), 0.98 (s, 9H, C*H_3_* *t*-Bu TBS), 0.19 (s, 6H, C*H_3_* Me-TBS), 0.19 (s, 6H, C*H_3_* Me-TBS).

In analogy to Duynstee *et al*. *Eur. J. Org. Chem*. **1999**, 2623-2632.

### Preparation of (6-(3,4-bis((tert-butyldimethylsilyl)oxy)phenethoxy)-6-oxohexyl)triphenyl phosphonium bromide **(15)**

Bromohexanoic acid (1.4 g, 7.2 mmol, 1.2 equiv.), DCC (1.49 g, 7.2 mmol, 1.2 equiv.) and DMAP (441 mg, 3.61 mmol, 0.6 equiv.) were added to a solution of **15** (2.3 g, 6 mmol, 1 equiv.) in 60 mL anhydrous CH_2_Cl_2_ and the mixture was stirred overnight at room temperature. The mixture was then filtered on Celite® and the filtrate concentrated under reduced pressure. The crude was then purified by silica gel column chromatography using pentane/EtOAc 90/10 as eluent to afford bromide (2.87 g, 5.12 mmol, 85%) as a colourless oil.

PPh_3_ (703 mg, 2.68 mmol, 1.5 equiv.) and K_2_CO_3_ (25 mg, 0.18 mmol, 0.1 equiv.) were added to a solution of the previously prepared bromide (1 g, 1.79 mmol, 1 equiv.) in 18 mL anhydrous MeCN and the mixture was stirred at reflux for 72 hours. The mixture was then concentrated under reduced pressure and the crude was purified by silica gel column chromatography using CH_2_Cl_2_ 100% then CH_2_Cl_2_/MeOH 90/10 as eluents to afford the phosphonium salt **15** (1 g, 1.22 mmol, 68%) as a white solid.

^1^H NMR (400 MHz, CDCl_3_): δ 7.91 – 7.63 (m, 15H, *H_Ar_* PPh_3_), 6.72 (d, ^3^*J*_H,H_ = 8.1 Hz, 2H, *H_Ar_*), 6.65 (d, ^4^*J*_H,H_ = 2.1 Hz, 2H, *H_Ar_*), 6.61 (dd, ^3^*J*_H,H_ = 8.1 Hz, ^4^*J*_H,H_ = 2.2 Hz, 2H, *H_Ar_*), 4.14 (t, ^3^*J*_H,H_ = 7.3 Hz, 2H, C*H_2_*-O), 3.99 – 3.85 (m, 2H, C*H_2_*-P), 2.76 (t, ^3^*J*_H,H_ = 7.3 Hz, 2H, C*H_2_*-CH_2_-O), 2.26 (t, ^3^*J*_H,H_ = 7.3 Hz, 2H, C*H_2_*-CO), 1.77 – 1.69 (m, 2H, C*H_2_*), 1.68 – 1.54 (m, 6H, C*H_2_*), 0.97 (s, 18H, C*H_3_* *t*-Bu TBS), 0.17 (s, 12H, C*H_3_* Me-TBS).

^31^P NMR (162 MHz, CDCl_3_): δ 24.7.

HRMS (ESI+) m/z: calcd for C_44_H_62_O_4_Si_2_ : 741.3919 [M-Br]^+^, found: 741.3913 [M-Br]^+^.

### Preparation of ethyl 10-(dimethoxyphosphoryl)-9-oxodecanoate (**24**)

A solution of *n*-BuLi in hexane (2.5 M in hexane, 16.8 mL, 42 mmol, 1.01 equiv.) was added dropwise to a solution of dimethyl methanephosphonate (4.5 mL, 41 mmol, 1 equiv.) in 55 mL anhydrous THF at –78°C. After stirring at –78°C for 10 minutes, diethyl azelate (16.9 mL, 67 mmol, 1.64 equiv.) was added dropwise, and the mixture was stirred at this temperature for 3.5 hours. Acetic acid (5 mL) was added, and the mixture was extracted with diethyl ether (2 x 150 mL). The gel-like mixture was treated by water (50 mL) and was extracted with CH_2_Cl_2_ (2 x 50 mL). The combined organic extracts were washed with water (50 mL), dried over MgSO_4_ and concentrated under reduced pressure. The excess of diethyl azelate was eliminated by distillation (100°C, 0.3 mbar). The residue was purified by silica gel column chromatography using cyclohexane/EtOAc gradient from 50/50 to 0/100, to afford phosphonate **24** (5.8 g, 18 mmol, 44%) as a colourless oil.

Rf = 0.35 (CH_2_Cl_2_/MeOH: 9/1).

^1^H NMR (400 MHz, CDCl_3_): δ 4.11 (q, ^3^*J*_H,H_ = 7.1 Hz, 2H), 3.79 (s, 3H), 3.77 (s, 3H), 3.07 (d, ^2^*J*_P,H_ = 22.7 Hz, 2H), 2.60 (t, ^3^*J*_H,H_ = 7.3 Hz, 2H), 2.27 (t, ^3^*J*_H,H_ = 7.5 Hz, 2H), 1.67 – 1.53 (m, 4H), 1.35 – 1.27 (m, 6H), 1.25 (t, ^3^*J*_H,H_ = 7.1 Hz, 1H).

In analogy to El Fangour *et al*. *J. Org. Chem*. **2004**, *69*, 2498–2503.

### Preparation of (4a*S*,5*S*,7*R*,7a*S*)-5,7-bis((tert-butyldimethylsilyl)oxy)octahydrocyclopenta [c]pyran-3-ol (**5**)

#### Preparation of (Z)-9-oxabicyclo[6.1.0]non-2-ene (**S5**)

Cycloocta-1,3-diene (57 mL, 0.46 mol, 1 equiv.) was dissolved in 750 mL CH_2_Cl_2_ under mechanical stirring. Na_2_CO_3_ (200 g, 1.89 mol, 4.1 equiv.) was added and the mixture cooled to 0°C before a dropwise addition of peracetic acid (40% in acetic acid, 100 mL, 0.61 mol, 1.3 equiv.) over 40 minutes (temperature never exceeded 7°C). The mixture was then stirred at room temperature for 2 hours (if the temperature exceeded 30°C, a water bath was added). The progress of the reaction was followed by ^1^H NMR. The mixture was then cooled to 0°C and quenched with a slow addition of NaHCO_3_ saturated solution (temperature never exceeded 10°C). The mixture was then filtered, 100 mL of CH_2_Cl_2_ were added to the filtrate and the phases were separated. The aqueous phase was extracted with CH_2_Cl_2_ (2 x 100 mL), the combined organic phases were washed with brine and dried over MgSO_4_. Solvents were removed under vacuum to afford epoxide **S5** as a colourless liquid (49.9 g, 0.40 mol, 87%).

^1^H NMR (400 MHz, CDCl_3_) : δ 5.77 (dddd, ^3^*J*_H,H_ = 11.2 Hz, ^3^*J*_H,H_ = 7.1 Hz, ^3^*J*_H,H_ = 5.6 Hz, ^4^*J*_H,H_ = 1.2 Hz, 1H, C*H*=CH), 5.59 (dquint, ^3^*J*_H,H_ = 11.2 Hz, ^4^*J*_H,H_ = 1.3 Hz, 1H, CH=C*H*), 3.45 (dt, ^3^*J*_H,H_ = 4.1 Hz, ^4^*J*_H,H_ = 1.3 Hz, 1H, O-C*H*), 3.11 (dtd, *^3^J*_H,H_ = 9.3 Hz, ^3^*J*_H,H_ = 3.9 Hz, ^4^*J*_H,H_ = 1.0 Hz, 1H, O-C*H*), 2.31 (dddq, ^2^*J*_H,H_ = 16.1 Hz, ^3^*J*_H,H_ = 10.0 Hz, ^3^*J*_H,H_ = 7.4 Hz, ^4^*J*_H,H_ = 1.5 Hz, 1H, C*H_2_*), 2.13 – 2.06 (m, 1H, C*H_2_*), 2.05 – 1.96 (m, 1H, C*H_2_*), 1.84 – 1.70 (m, 1H, C*H_2_*), 1.71 – 1.60 (m, 2H, C*H_2_*), 1.53 – 1.35 (m, 2H, C*H_2_*). In analogy to Oger *et al*. *Org. Lett*. **2008**, *10*, 5087–5090.

#### Preparation of compound (1S,3aS,6aS)-1,2,3,3a,4,6a-hexahydropentalen-1-ol (**rac-8**)

In a 2-litres 3-neck round bottom flask with a condenser equipped with a CaCl_2_ moisture trap, a solution of *n*-BuLi (11 M in hexanes, 100 mL, 1.10 mol, 1.1 equiv.) was slowly added, with a canula, to a solution of *N,N*–diethylamine (113 mL, 1.10 mol, 1.1 equiv.) in 750 mL anhydrous Et_2_O at 0 °C. After addition the reaction was stirred at 0 °C for 1 h, and the mixture was heated at 30 °C with a water bath. Then, 3,4-epoxycyclooctene **S5** (49.9 g, 0.40 mol, 1 equiv., solution in 100 mL anhydrous Et_2_O) was added dropwise (temperature should never exceed 35°C). The CH-insertion reaction taking place almost instantly at this temperature, after a few mL of dropping addition, the water bath was removed and the temperature was kept at around 30 °C by the reaction’s exothermicity (note that adding the epoxide at room temperature, then heat up to 30 °C caused extremely fast reaction and build-up of high risk of over-pressure). After addition, the reaction was stirred at around 30 °C for 2 hours. The mixture was then cooled to 0 °C and quenched by the addition of aqueous HCl (5M, around 500 mL) with vigorous stirring until pH = 1 (note that HCl addition was controlled to never exceed a temperature of 10°C). The aqueous and organic layers were separated. The aqueous phase was extracted with Et_2_O (2 x 250 mL), the combined organic phases were washed with saturated NaHCO_3_ (200 mL), brine (100 mL) and dried over MgSO_4_. After concentration under vacuum, the crude was purified by distillation (3 mbar, 80 °C to 105 °C) to afford the pure alcohol **rac-8** (34.6 g, 0.28 mol, 69%) as a colourless liquid.

^1^H NMR (400 MHz, CDCl_3_)**:** δ 5.94 – 5.83 (m, 1H, C*H*=CH), 5.64 – 5.58 (m, 1H, CH=C*H*), 4.21 (apparent quint, ^3^*J*_H,H_ = 5.7 Hz, 1H, C*H*-OH), 3.31 – 3.20 (m, 1H), 2.75 – 2.60 (m, 2H), 2.14 – 2.02 (m, 1H), 1.90 – 1.76 (m, 1H), 1.74 – 1.63 (m, 1H), 1.66 – 1.53 (m, 1H), 1.45 – 1.33 (m, 2H). In analogy to Oger *et al*. *Org. Lett*. **2008**, *10*, 5087–5090.

#### Preparation of (1S,3aS,6aS)-1,2,3,3a,4,6a-hexahydropentalen-1-ol (**8**) and (1R,3aR,6aR)-1,2,3,3a,4,6a-hexahydropentalen-1-ol (***ent*-8**)

Succinic anhydride (16.17 g, 16.16 mmol, 1 equiv.) and the *Amano AK* lipase (from *Pseudomonas fluorescent*, approx. 20000 U/g, 1.01 g) were successively added to a solution of the racemic alcohol **rac-8** (34.6 g, 0.28 mol, 1 equiv.) in 560 mL of Et_2_O. The suspension was stirred at 150 rpm using the rotavapor for 16 hours. Upon reaching 50% conversion, the suspension was filtered and washed with Et_2_O (100 mL). The filtrate was washed with a 2M solution of NaHCO_3_ (3 x 150 mL) and the layers separated. The aqueous layer was extracted with Et_2_O (3 x 150 mL) and the combined organic layers were washed with a 10% solution of NaCl/Na_2_CO_3_ (1/1, 2 x 150 mL), dried over MgSO_4_, filtered and the solvent was removed under reduced pressure. The (1*S*,3a*S*,6a*S*)-alcohol **8** was obtained as a yellow oil (17.89 g, 52%, ee > 99.5%)

NaOH pellets were added to the aqueous layer at 10 °C until the obtention of a basic pH and stirred for 1 hour. At the end of the saponification, Et_2_O (300 mL) was added, the layers were separated, and the aqueous phase was extracted with Et_2_O (2 x 150 mL). The combined organic layers were dried over MgSO_4_, filtered and the solvent removed under reduced pressure. The (1*R*,3a*R*,6a*R*)-alcohol ***ent*-8** was obtained as a colourless oil (14.57 g, 42%, ee >99.5%).

^1^H NMR (400 MHz, CDCl_3_)**:** δ 5.94 – 5.83 (m, 1H, C*H*=CH), 5.64 – 5.58 (m, 1H, CH=C*H*), 4.21 (apparent quint, ^3^*J*_H,H_ = 5.7 Hz, 1H, C*H*-OH), 3.31 – 3.20 (m, 1H), 2.75 – 2.60 (m, 2H), 2.14 – 2.02 (m, 1H), 1.90 – 1.76 (m, 1H), 1.74 – 1.63 (m, 1H), 1.66 – 1.53 (m, 1H), 1.45 – 1.33 (m, 2H). In analogy to Oger *et al*. *Org. Lett*. **2008**, *10*, 5087–5090.

Compound **8**: [α]_D_^20^ = -153 (10 mg/mL, EtOH)

Compound ***ent*-8**: [α]_D_^20^ = +196 (10 mg/mL, EtOH)

##### Determination of the enantiomeric excess of **8** and ***ent*-8**

1. Derivatisation - preparation of compound **S6**

The 3,5-dinitrobenzoyl chloride (1.0 g, 4.34 mmol, 2.7 equiv.) was added to a solution of the alcohol **8** (50 mg, 0.40 mmol, 1 equiv.) in 1.2 mL of anhydrous pyridine at room temperature and under N_2_. After 1 hour at room temperature, the reaction was quenched by the addition of HCl 1M (10 mL) and filtered. The filtrate was extracted with Et_2_O (2 x 40 mL). The combined organic layers were washed with a HCl solution (1M, 2 x 30 mL), a saturated solution (2 x 30 mL), and brine, then dried over MgSO_4_ and concentrated under reduced pressure. The dinitro-derivative **S6** was obtained as a white powder (85 mg, 67%). The same procedure was applied to ***ent*-8** and **rac-8** compounds.

1. HPLC conditions:

HPLC apparatus: HPLC Perkin Helmer serie 200

Column: Chiral column Chiralcel OD 0.46 cm x 25 cm,

Detection: Diode Array Detector (λ=254 nm)).

Flow: 0.7 mL/min.

Injection volume: 10 µL

Solvent: Hexane/*i*-PrOH: 96/4 (v/v)

1. Enantiomeric excess

*See chromatograms below.*


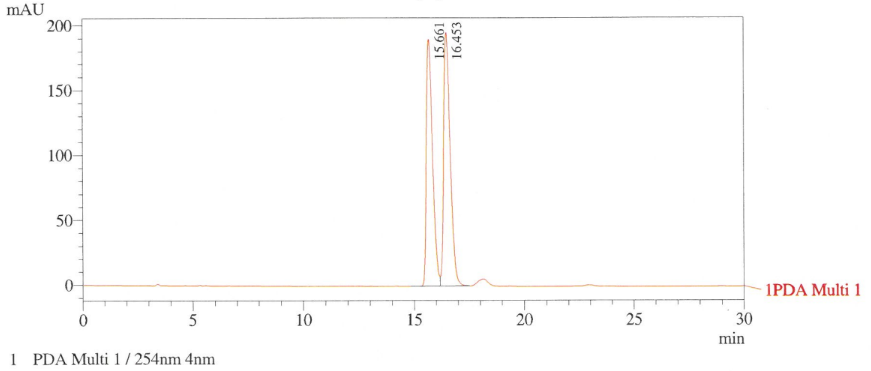

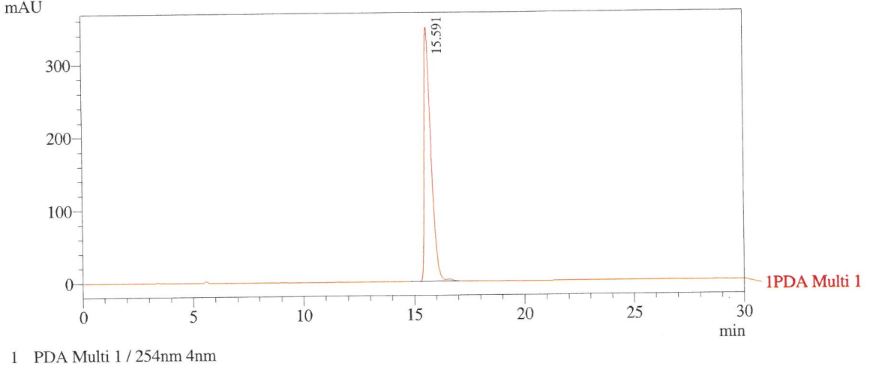

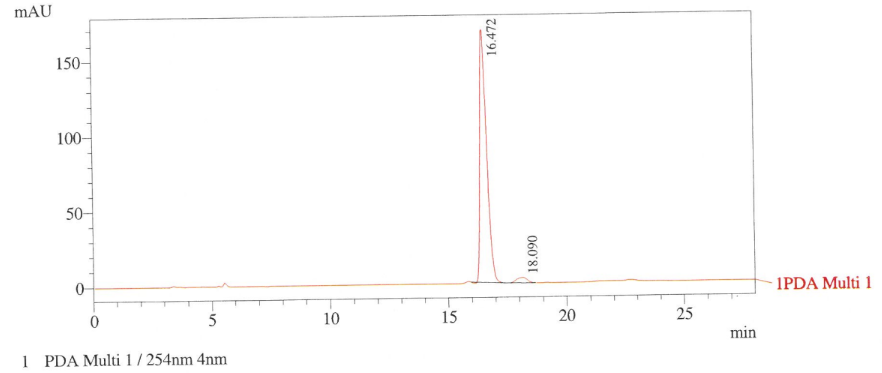


> 99.5%

98%

2%

**8**

***ent*-8**

**rac-8**

2%

50%

48%

#### Preparation of (3a*R*,6a*R*)-4,6a-dihydropentalen-1(3a*H*)-one (**9**) and (3aS,6aS)-4,6a-dihydropentalen-1(3aH)-one (*ent*-**9**)

In a 3-neck round bottom flask equipped with a mechanical stirrer, alcohol **8** (7.1 g, 57 mmol, 1 equiv.) was added to a solution of TsOH.H_2_O (5.42 g, 28.50 mmol, 0.5 equiv.) in 120 mL DMSO (analytical grade). IBX (**S1)** (48.7 g, 174 mmol, 3 equiv.) was then added in four portions (approximately 20 minutes between each addition). After the last IBX addition, the mixture was stirred at 50 °C for 30 minutes followed by 3 hours at 70 °C. After cooling to room temperature, the resulting mixture was filtered on Celite® and the white cake rinsed with DMSO (3 x 20 mL). A cold solution of saturated NaHCO_3_ (300 mL) was then added to the filtrate and stirred vigorously for 15 minutes. The mixture was once again filtered on Celite® and extracted by Et_2_O (3 x 150 mL). The combined organic phases were washed with brine (3 x 100 mL), dried over MgSO_4_ and the solvents were removed under reduced pressure (carefully due to the volatility of the product) to afford the bicyclic enone **9** as a pale-yellow liquid (5.5 g, 45.80 mmol, 80%).

The same procedure was applied onto ***ent*-8** alcohol to produce ***ent*-9** enone (85 % yield).

^1^H NMR (400 MHz, CDCl_3_): δ 7.52 (dd, ^3^*J*_H,H_ = 5.6 Hz, ^4^*J*_H,H_ = 2.7 Hz, 1H, C*H*=CH-CO), 6.04 (dd, ^3^*J*_H,H_ = 5.7 Hz, ^4^*J*_H,H_ = 1.9 Hz, 1H, CH=C*H*-CO), 5.71 – 5.62 (m, 2H, C*H*=C*H*), 3.60 – 3.53 (m, 1H), 3.46 – 3.42 (m, 1H), 2.79 – 2.68 (m, 1H), 2.34 – 2.24 (m, 1H). In analogy to Oger *et al*. *Org. Lett*. **2008**, *10*, 5087–5090.

#### Preparation of (1a*R*,1b*S*,4a*R*,5a*R*)-1a,2,4a,5a-tetrahydropentaleno[1,2-b]oxiren-5(1b*H*)-one (**10**) and (1aS,1bR,4aS,5aS)-1a,2,4a,5a-tetrahydropentaleno[1,2-b]oxiren-5(1bH)-one (*ent*-**10**)

Distilled water (100 mL) was added to a solution of *tert*-butyl hydroperoxide (70 wt. % in water, 12.5 mL, 91.6 mmol, 2 equiv.) in 200 mL CH_2_Cl_2_. The layers were separated, the organic phase was dried over MgSO_4_ and filtered. This solution was cooled to 0 °C under N_2_, prior to the addition of DBU (8.2 mL, 55 mmol, 1.2 equiv.), followed by a dropwise addition (over 30 minutes) of enone **9** (5.5 g, 45.8 mmol, 1 equiv.) in solution with 30 mL CH_2_Cl_2_. The mixture was stirred for 30 minutes at 0 °C and at room temperature overnight. The mixture was then treated with a freshly prepared solution of Na_2_SO_3_ (12 g in 140 mL of H_2_O), the layers were separated and the organic phase was washed with brine (3 x 100 mL), dried over MgSO4 and concentrated under pressure (carefully due to the volatility of the compound). The crude was then purified on a short silica gel column chromatography with a gradient pentane/Et_2_O, from 80/20 to 60/40, to afford the pure keto-epoxide **10** as a pale-yellow oil (5.2 g, 38.20 mmol, 83%).

The same procedure was applied onto ***ent*-9** alcohol to produce ***ent*-10** epoxide (67% yield).

^1^H NMR (400 MHz, CDCl_3_): δ 5.80 (dq, ^3^*J*_H,H_ = 5.5 Hz, ^4^*J*_H,H_ = 2.3 Hz, 1H, C*H*=CH), 5.61 (dq, ^3^*J*_H,H_ = 5.1 Hz, ^2^*J*_H,H_ = 2.5 Hz, 1H, CH=C*H*), 3.73 – 3.69 (m, 1H), 3.41 – 3.34 (m, 2H, including dd ^4^*J*_H,H_ = 2.2 Hz, ^4^*J*_H,H_ = 0.8 Hz), 3.28 (ddd, ^3^*J*_H,H_ = 10.4 Hz, ^3^*J*_H,H_ = 7.6 Hz, ^3^*J*_H,H_ = 4.9 Hz, 1H), 2.74 – 2.65 (m, 1H), 2.40 – 2.30 (m, 1H). In analogy to Oger *et al*. *Org. Lett*. **2008**, 10, 5087–5090.

#### Preparation of (1*R*,3*S*,3a*S*,6a*R*)-1,2,3,3a,4,6a-hexahydropentalene-1,3-diol (**11**) and (1S,3R,3aR,6aS)-1,2,3,3a,4,6a-hexahydropentalene-1,3-diol (***ent*-11**)

A solution of LiAlH_4_ (2.4 M in THF, 25.5 mL, 61 mmol, 1.8 equiv.) was added dropwise at –78 °C to a solution of epoxide **10** (4.5 g, 33 mmol, 1 equiv.) in 250 mL anhydrous THF. The mixture was stirred at –78 °C for 30 minutes followed by 40 minutes at 0 °C. The mixture was then quenched by the dropwise addition of MeOH (15 mL, exothermic) and the crude was stirred vigorously for 2 hours with a Na/K tartrate solution (Rochelle salt, 1M, 200 mL). The precipitate was filtered on Celite® and rinsed with EtOAc (150 mL). The resulting phases were separated, the aqueous one extracted with EtOAc (2 x 100 mL), and the combined organic layers were washed with brine, dried over MgSO_4_, and concentrated under reduced pressure. The crude was then purified by silica gel column chromatography with a gradient pentane/EtOAc from 90/10 to 60/40 as eluents to afford the *cis*-diol **11** (2.86 g, 20.40 mmol, 65%) as a colourless oil. ^1^H NMR estimation based on the alkene protons shows approximately 6% of the *trans* diol (dr = 94/6 *cis*/*trans*, will be separated at the next stages).

The same procedure was applied onto ***ent*-10** epoxide to produce ***ent*-11** diol (62% yield).

^1^H NMR (400 MHz, CDCl_3_): δ 5.53 – 5.49 (m, 1H, C*H*=CH), 5.49 – 5.45 (m, 1H, CH=C*H*), 4.01 (q, ^3^*J*_H,H_ = 5.4 Hz, ^3^*J*_H,H_ = 4.9 Hz, 2H), 3.36 – 3.29 (m, 1H), 2.91 – 2.81 (m, 1H), 2.71 – 2.58 (m, 3H), 1.97 – 1.89 (m, 1H), 1.84 – 1.77 (m, 1H), 1.62 (dt, ^2^*J*_H,H_ = 14.3 Hz, ^3^*J*_H,H_ *=* 4.3 Hz, 1H). In analogy to Oger *et al*. *Org. Lett*. **2008**, *10*, 5087–5090.

Note that the *trans* isomer was fully characterised in Oger *et al*. *Org. Lett*. **2008**, *10*, 5087–5090.

#### Preparation of 2-((1*S*,2*S*,3*R*,5*S*)-3,5-bis((tert-butyldimethylsilyl)oxy)-2-(hydroxymethyl)cyclopentyl) ethan-1-ol (**12**) and 2-((1R,2R,3S,5R)-3,5-bis((tert-butyldimethylsilyl)oxy)-2-(hydroxymethyl) cyclopentyl)ethan-1-ol (***ent*-12**)

Imidazole (5.35 g, 78.60 mmol, 5.3 equiv.), TBSCl (5.92 g, 39.30 mmol, 2.65 equiv.) and DMAP (18 mg, 0.15 mmol, 0.01 equiv.) were successively added to a solution of the diol **11** (2.06 g, 14.80 mmol, 1 equiv.) in 22 mL anhydrous DMF and the mixture was stirred overnight at room temperature. Then, water was added (100 mL) and the crude extracted by Et_2_O (3 x 100 mL). The combined organic layers were washed with water (2 x 100 mL), brine (3 x 100 mL), dried over MgSO_4_, filtered and the solvents removed under reduced pressure. The crude material was left 48 hours under high vacuum to recover the protected diol **S7** as a colourless liquid (5.42 g, 14.70 mmol, 99%; presence of small traces of residual silanol by-product).

^1^H NMR (400 MHz, CDCl_3_): δ 5.72 – 5.66 (m, 1H, C*H*=CH), 5.60 – 5.55 (m, 1H, CH=C*H*), 3.87 – 3.78 (m, 1H), 3.65 (dt, ^3^*J*_H,H_ = 9.7 Hz, ^3^*J*_H,H_ = 6.2 Hz, 1H), 3.07 – 2.99 (m, 1H), 2.69 – 2.58 (m, 1H), 2.55 – 2.44 (m, 1H), 2.21 – 2.13 (m, 1H), 2.02 (dt, ^2^*J*_H,H_ = 11.8 Hz, ^3^*J*_H,H_ = 5.9 Hz, 1H), 1.74 – 1.62 (m, 1H), 0.89 (s, 9H, C*H_3_* *t*-Bu TBS), 0.88 (s, 9H, C*H_3_* *t*-Bu TBS), 0.06 (s, 3H, C*H_3_* Me-TBS), 0.05 (s, 3H, C*H_3_* Me-TBS), 0.04 (s, 3H, C*H_3_* Me-TBS), 0.03 (s, 3H, C*H_3_* Me-TBS). In analogy to Oger *et al*. *Org. Lett*. **2008**, 10, 5087–5090.

Ozone was bubbled to a solution of protected diol **S7** (5.42 g, 14.70 mmol, 1 equiv.) in anhydrous CH_2_Cl_2_/MeOH (60 mL/6.5 mL) at –80 °C for 30 minutes. When a blue coloration indicating an excess of ozone, the reaction was flushed with nitrogen, and NaBH_4_ (5.56 g, 147 mmol, 10 equiv.) was added. The mixture was gradually allowed to warm up to room temperature. Then, water was added and the mixture was stirred vigorously at room temperature overnight. The layers were separated and the aqueous phase extracted with CH_2_Cl_2_ (2 x 80 mL). The combined organic layers were washed with brine (80 mL), dried over MgSO_4_, filtered and the solvents removed under reduced pressure to afford the protected tetraol **12** as a white solid (5.35 g, 13.20 mmol, 90%).

^1^H NMR (400 MHz, CDCl_3_): δ 4.04 – 3.96 (m, 1H), 3.85 (q, ^3^*J*_H,H_ = 6.7 Hz, 1H), 3.79 – 3.64 (m, 3H), 3.59 (dd, ^2^*J*_H,H_ = 10.7 Hz, ^3^*J*_H,H_ = 6.1 Hz, 1H), 2.30 (dt, ^2^*J*_H,H_ = 13.6 Hz, ^3^*J*_H,H_ = 7.0 Hz, 1H), 2.27 – 2.14 (m, 2H), 1.78 – 1.66 (m, 1H), 1.57 – 1.46 (m, 3H), 0.89 (s, 9H, C*H_3_* *t*-Bu TBS), 0.88 (s, 9H, C*H_3_* *t*-Bu TBS), 0.06 (s, 6H, C*H_3_* Me-TBS), 0.05 (s, 6H, C*H_3_* Me-TBS). In analogy to Oger *et al*. *Org. Lett*. **2008**, *10*, 5087–5090.

The same procedure was applied onto ***ent*-11** diol to produce ***ent*-12** tetraol (93% yield).

^1^H NMR (400 MHz, CDCl_3_): δ 4.00 (td, ^3^*J*_H,H_ = 6.6 Hz, ^3^*J*_H,H_ = 4.3 Hz, 1H), 3.85 (q, ^3^*J*_H,H_ = 6.7 Hz, 1H), 3.78 – 3.65 (m, 3H), 3.59 (dd, ^2^*J*_H,H_ = 10.7 Hz, ^3^*J*_H,H_ = 6.0 Hz, 1H), 2.30 (dt, ^2^*J*_H,H_ = 13.6 Hz, ^3^*J*_H,H_ = 7.0 Hz, 1H), 2.26 – 2.15 (m, 2H), 1.94 (br s, 1H), 1.79 – 1.67 (m, 1H), 1.56 – 1.46 (m, 3H), 0.89 (s, 9H), 0.88 (s, 9H), 0.06 (s, 6H), 0.05 (s, 6H). In analogy to Oger *et al*. *Org. Lett*. **2008**, *10*, 5087–5090.

#### Preparation of (4a*S*,5*S*,7*R*,7a*S*)-5,7-bis((tert-butyldimethylsilyl)oxy)hexahydrocyclopenta[c]pyran-3(1*H*)-one (**13**)

Iridium catalyst **S2** (80 mg, 0.15 mmol, 0.8 mol%) was added to a solution of tetraol **12** (7.5 g, 18.60 mmol, 1 equiv.) in 68 mL freshly distilled butanone. The mixture was stirred at reflux for 24 hours. The resulting solution was then removed under reduced pressure, and the residue was purified through a small silica gel column chromatography (eluent pentane/Et_2_O, 9/1) to give the lactone **13** (6.94 g, 17.30 mmol, 93%) as a white solid.

^1^H NMR (400 MHz, CDCl_3_): δ 4.25 – 4.16 (m, 2H), 3.97 (ddd, ^3^*J*_H,H_ = 10.8 Hz, ^3^*J*_H,H_ = 7.6 Hz, ^3^*J*_H,H_ = 5.9 Hz, 1H), 3.67 – 3.56 (m, 1H), 2.58 – 2.46 (m, 3H), 2.39 – 2.29 (m, 1H), 2.05 (dt, ^2^*J*_H,H_ = 11.6 Hz, ^3^*J*_H,H_ = 5.8 Hz, 1H), 1.64 (q, ^3^*J*_H,H_ = 10.8 Hz, 1H), 0.88 (s, 9H), 0.88 (s, 9H), 0.07 (s, 3H, C*H_3_* Me-TBS), 0.06 (s, 3H, C*H_3_* Me-TBS), 0.06 (s, 3H, C*H_3_* Me-TBS), 0.05 (s, 3H, C*H_3_* Me-TBS). In analogy to Oger *et al*. *Org. Lett*. **2008**, *10*, 5087–5090.

#### Preparation of (4a*S*,5*S*,7*R*,7a*S*)-5,7-bis((tert-butyldimethylsilyl)oxy)octahydrocyclopenta[c]pyran-3-ol (**5**)

DIBAlH (1M in CH_2_Cl_2_, 7.5 mL, 7.50 mmol, 1.5 equiv.) was slowly added to a solution of lactone **13** (920 mg, 5 mmol, 1 equiv.) in 80 mL CH_2_Cl_2_ at –78 °C. After 30 min at the same temperature, the reaction was quenched by the addition of a 10% NH_4_Cl solution (30 mL). The reaction mixture was warmed up to room temperature and a solution of Na/K tartrate (Rochelle salt, 1M, 100 mL) was added. After stirring for 1 hour, the layers were separated and the aqueous phase was extracted with CH_2_Cl_2_ (3 x 60 mL). The combined organic layers were washed with brine, dried over MgSO_4_ and the solvent was removed under reduced pressure. The crude was then purified by silica gel column chromatography with pentane/EtOAc : 70/30 as eluent to afford lactol **5** (1.96 g, 4.87 mmol, 98%) as a colourless oil.

^1^H NMR (400 MHz, CDCl_3_): δ 5.17 (q, ^4^*J*_H,H_ = 3.6 Hz, 0.5H), 4.68 (ddd, ^3^*J*_H,H_ = 8.9 Hz, ^3^*J*_H,H_ = 5.3 Hz, ^3^*J*_H,H_ = 2.3 Hz, 0.5H), 4.25 – 4.11 (m, 1H), 4.07 (dd, ^2^*J*_H,H_ = 11.8 Hz, ^4^*J*_H,H_ = 3.9 Hz, 0.5H), 3.99 (dd, ^2^*J*_H,H_ = 12.0 Hz, ^4^*J*_H,H_ = 1.3 Hz, 0.5H), 3.88 – 3.83 (m, 0.5H), 3.82 – 3.76 (m, 0.5H), 3.69 (dd, ^2^*J*_H,H_ = 12.1 Hz, ^3^*J*_H,H_ = 3.1 Hz, 0.5H), 3.55 (dd, ^2^*J*_H,H_ = 11.8 Hz, ^3^*J*_H,H_ = 2.7 Hz, 0.5H), 2.70 (d, ^3^*J*_H,H_ = 5.3 Hz, 0.5H), 2.47 – 2.33 (m, 1.5H), 2.32 – 2.23 (m, 0.5H), 2.13 – 2.00 (m, 2H), 1.86 – 1.76 (m, 1H), 1.66 – 1.57 (m, 1H), 1.45 – 1.33 (m, 1H), 1.08 – 0.92 (m, 1H), 0.88 (d, ^3^*J*_H,H_ = 1.4 Hz, 9H, C*H_3_* *t*-Bu TBS), 0.88 (s, 9H, C*H_3_* *t*-Bu TBS), 0.05 (s, 3H, C*H_3_* Me-TBS), 0.06 (s, 3H, C*H_3_* Me-TBS), 0.03 (s, 6H, C*H_3_* Me-TBS). In analogy to Oger *et al*. *Org. Lett*. **2008**, *10*, 5087–5090.

### Preparation of 2-((1*R*,2*R*,3*S*,5*R*)-3,5-bis((tert-butyldimethylsilyl)oxy)-2-(((triethylsilyl)oxy)methyl) cyclopentyl)ethan-1-ol (**6**)

#### Preparation of 2-((1*R*,2*R*,3*S*,5*R*)-3,5-bis((tert-butyldimethylsilyl)oxy)-2-(((triethylsilyl)oxy)methyl) cyclopentyl)ethyl acetate (**14**)

Supported CALB (approx. 11000 U/g, 600 mg) was added to a solution of tetraol ***ent*-12** (3 g, 7.41 mmol, 1 equiv.) in 150 mL THF (HPLC grade) and 150 mL vinyl acetate. The mixture was stirred at room temperature overnight with the rotavapor (150 rpm), then filtered on Celite® and the enzyme washed with Et_2_O (100 mL). The filtrate was concentrated, and the crude was purified by silica gel column chromatography using a gradient pentane/Et_2_O, from 90/10 to 70/30, affording pure **S8** (3.14 g, 7.03 mmol, 95%).

Rf = 0.45 (cyclohexane/EtOAc: 7/3).

^1^H NMR (400 MHz, CDCl_3_): δ 4.16 – 4.08 (m, 2H), 4.07 – 4.01 (m, 1H), 3.83 (q, ^3^*J*_H,H_ = 6.5 Hz, 1H), 3.71 (dd, ^2^*J*_H,H_ = 10.7 Hz, ^3^*J*_H,H_ = 5.6 Hz, 1H), 3.60 (dd, ^2^*J*_H,H_ = 10.7 Hz, ^3^*J*_H,H_ = 7.0 Hz, 1H), 2.31 (dt, ^3^*J*_H,H_ = 13.7 Hz, ^3^*J*_H,H_ = 7.1 Hz, 1H), 2.25 – 2.17 (m, 1H), 2.15 – 2.07 (m, 1H), 2.04 (s, 3H), 1.73 – 1.47 (m, 4H), 0.88 (s, 18H), 0.05 (s, 6H), 0.04 (s, 3H), 0.03 (s, 3H). In analogy with Oger *et al*. *J. Org. Chem.* **2010**, *75*, 1892–1897.

TESCl (811 µL, 4.83 mmol, 1.2 equiv.), DMAP (49 mg, 0.40 mmol, 0.1 equiv.) and imidazole (658 mg, 9.67 mmol, 2.4 equiv.) were successively added to a solution of **S8** (1.8 g, 4.03 mmol, 1 equiv.) in 45 mL anhydrous CH_2_Cl_2_. The mixture was stirred at room temperature for 4 hours and was then filtered on Celite®. The filtrate was concentrated under reduced pressure and the crude was then purified by silica gel column chromatography using a gradient pentane/Et_2_O, from 98/2 to 90/10, to afford the pure **14** (2.04 g, 3.64 mmol, 90%) as a colourless oil.

Rf = 0.62 (cyclohexane/EtOAc: 9/1).

IR (neat): ν = 2955, 2857 (CH), 1743 (C=O) cm^-1^.

^1^H NMR (400 MHz, CDCl_3_): δ 4.12 (t, ^3^*J*_H,H_ = 7.3 Hz, 2H, *H_8_*), 4.09 – 4.04 (m, 1H, *H_3_*), 3.86 (q, ^3^*J*_H,H_ = 7.4 Hz, 1H, *H_5_*), 3.60 (dd, ^2^*J*_H,H_ = 10.4 Hz, ^3^*J*_H,H_ = 4.7 Hz, 1H, *H_1a_*), 3.55 (dd, ^3^*J*_H,H_ = 10.4 Hz, ^3^*J*_H,H_ = 4.9 Hz, 1H, *H_1a_*), 2.29 (dt, ^3^*J*_H,H_ = 13.4 Hz, ^3^*J*_H,H_ = 7.2 Hz, 1H, *H_4a_*), 2.20 – 2.10 (m, 1H, *H_6_*), 2.04 (s, 3H, C*H_3_* Ac), 2.02 – 1.95 (m, 1H, *H_2_*), 1.80 – 1.63 (m, 2H, *H_7_*), 1.53 – 1.43 (m, 1H, *H_4b_*), 0.95 (t, ^3^*J*_H,H_ = 7.9 Hz, 9H, C*H_3_* TES), 0.88 (s, 9H, C*H_3_* *t*-Bu TBS), 0.87 (s, 9H, C*H_3_* *t*-Bu TBS), 0.58 (q, ^3^*J*_H,H_ = 7.9 Hz, 6H, C*H_2_* TES), 0.03 (s, 6H, C*H_3_* Me-TBS), 0.02 (s, 6H, C*H_3_* Me-TBS).

^13^C NMR (126 MHz, CDCl_3_): δ 171.3 (*C*O Ac), 76.9 (*C_5_*), 73.7 (*C_3_*), 64.3 (*C_8_*), 61.3 (*C_1_*), 50.9 (*C_2_*), 45.0 (*C_4_*), 44.7 (*C_6_*), 27.2 (*C_7_*), 26.0 (6C, *C*H_3_ t-Bu TBS), 21.2 (*C*H_3_ Ac), 18.2 (*C_q_* TBS), 18.1 (*C_q_* TBS), 7.0 (3C, *C*H_2_ TES), 4.4 (3C, *C*H_3_ TES), -4.2 (*C*H_3_ Me-TBS), -4.4 (*C*H_3_ Me-TBS), -4.6 (*C*H_3_ Me-TBS), -4.6 (*C*H_3_ Me-TBS).

HRMS (TOF ESI+) m/z: calcd for C_28_H_61_O_5_Si_3_ : 561.3821 [M+H]^+^, found: 561.3821 [M+H]^+^.

[α]_D_^20^ = − 16.7 (8mg/mL EtOH).

#### Preparation of 2-((1*R*,2*R*,3*S*,5*R*)-3,5-bis((tert-butyldimethylsilyl)oxy)-2-(((triethylsilyl)oxy)methyl) cyclopentyl)ethan-1-ol (**6**)

K_2_CO_3_ (739 mg, 5.35 mmol, 1.5 equiv.) was added to a solution of **14** (2 g, 3.57 mmol, 1 equiv.) in 36 mL anhydrous MeOH. The mixture was stirred vigorously at room temperature for 30 minutes and was then filtered on Celite®. The filtrate was diluted with Et_2_O (80 mL) and washed with HCl 1M (80 mL) and brine (80 mL). The organic phase was dried over MgSO_4_ and concentrated under reduced pressure. The crude was then purified by silica gel column chromatography using a gradient pentane/Et_2_O, from 90/10 to 80/20, to afford the pure alcohol **6** (1.12 g, 2.16 mmol, 61%) as a colourless oil. Note that tetraol ***ent*-12** (478 mg, 1.18 mmol, 33%) was also recovered as a by-product from the reaction.

Rf = 0.30 (cyclohexane/EtOAc: 9/1).

IR (neat): ν = 2954, 2928, 2852 (CH) cm^-1^.

^1^H NMR (400 MHz, CDCl_3_): δ 4.03 (ddd, ^3^*J*_H,H_ = 7.3 Hz, ^3^*J*_H,H_ = 5.0 Hz, ^4^*J*_H,H_ = 3.0 Hz, 1H, *H_3_*), 3.90 (q, ^3^*J*_H,H_ = 7.4 Hz, 1H, *H_5_*), 3.70 (q, ^3^*J*_H,H_ = 6.0 Hz, 2H, *H_8_*), 3.59 (dd, ^2^*J*_H,H_ = 10.3 Hz, ^3^*J*_H,H_ = 5.3 Hz, 1H, *H_1a_*), 3.52 (dd, ^3^*J*_H,H_ = 10.3 Hz, ^3^*J*_H,H_ = 5.0 Hz, 1H, *H_1b_*), 2.61 (t, ^3^*J*_H,H_ = 6.0 Hz, 1H, O*H*), 2.36 – 2.19 (m, 2H, *H_4a_*, *H_6_*), 2.07 – 1.96 (m, 1H, *H_2_*), 1.82 – 1.69 (m, 1H, *H_7a_*), 1.66 – 1.57 (m, 1H, *H_7b_*), 1.55 – 1.47 (m, 1H, *H_4b_*), 0.96 (t, ^3^*J*_H,H_ = 7.9 Hz, 9H, C*H_3_* TES), 0.89 (s, 9H, C*H_3_* *t*-Bu TBS), 0.87 (s, 9H, C*H_3_* *t*-Bu TBS), 0.59 (q, ^3^*J*_H,H_ = 7.9 Hz, 6H, C*H_2_* TES), 0.06 (s, 3H, C*H_3_* Me-TBS), 0.06 (s, 3H, C*H_3_* Me-TBS), 0.03 (s, 3H, C*H_3_* Me-TBS), 0.03 (s, 3H, C*H_3_* Me-TBS).

^13^C NMR (126 MHz, CDCl_3_): δ 77.6 (*C_5_*), 73.7 (*C_3_*), 62.6 (*C_8_*), 61.6 (*C_1_*), 52.0 (*C_2_*), 45.5 (*C_6_*), 45.0 (*C_4_*), 31.5 (*C_7_*), 26.0 (6C, *C*H_3_ t-Bu TBS), 18.1 (*C_q_* TBS), 6.9 (3C, *C*H_2_ TES), 4.4 (3C, *C*H_3_ TES), -3.9 (*C*H_3_ Me-TBS), -4.4 (*C*H_3_ Me-TBS), -4.5 (*C*H_3_ Me-TBS), -4.6 (*C*H_3_ Me-TBS).

HRMS (TOF ESI+) m/z: calcd for C_26_H_59_O_4_Si_3_ : 519.3716 [M+H]^+^, found: 519.3722 [M+H]^+^.

[α]_D_^20^ = − 15.3 (8 mg/mL EtOH).

## Towards the synthesis of the 16-F_1t_-PhytoP-HT and its C16-epimer

### Preparation of methyl (*Z*)-8-((1*S*,2*S*,3*R*,5*S*)-3,5-bis((tert-butyldimethylsilyl)oxy)-2-(hydroxymethyl)cyclopentyl)oct-6-enoate (**19**)

A solution of *t*-BuOK (1 M in THF, 19.9 mL, 19.90 mmol, 4.2 equiv.) was added at 0 °C to a solution of commercial (5-carboxypentyl)triphenylphosphonium bromide (4.76 g, 10.40 mmol, 2.2 equiv.) in 100 mL anhydrous degassed THF. The mixture was stirred for 20 minutes at 0 °C and was then canulated to a solution of the lactol **5** (1.9 g, 4.7 mmol, 1 equiv.) in 50 mL THF. The resulting mixture was stirred at 0 °C for 30 minutes followed by 2 hours at room temperature and was then quenched by the addition of saturated aqueous NH_4_Cl solution (50 mL). The mixture was extracted with Et_2_O (2 x 100 mL) and the combined organic layers were washed with brine, dried over MgSO_4_, filtered, and concentrated under reduced pressure. TMSCHN_2_ (2 M in hexanes, 4.7 mL, 9.40 mmol, 2 equiv.) was then slowly added to a solution of the crude material in anhydrous Et_2_O/MeOH (9/1 : v/v, 100 mL). The mixture was stirred at room temperature for 4 hours and then quenched by a dropwise addition of AcOH until no gassing was observed. Solvents were removed under reduced pressure and the crude was purified by silica gel column chromatography using a gradient pentane/Et_2_O from 90/10 to 80/20, to afford ester **19** (1.45 g, 2.82 mmol, 60% over 2 steps) as a colourless oil.

Rf = 0.5 (cyclohexane/EtOAc: 7/3).

^1^H NMR (400 MHz, CDCl_3_): δ 5.45 – 5.32 (m, 2H, *H_6_*, *H_7_*), 4.06 (q, *^3^J_H,H_* = 6.5 Hz, 1H, *H_12_*), 3.81 (q, *^3^J_H,H_* = 5.3 Hz, 1H, *H_10_*), 3.71 (dd, *^3^J_H,H_* = 10.6, *^3^J_H,H_* = 5.8 Hz, 1H, *H_14a_*), 3.67 (s, 3H, COOC*H_3_*), 3.64 (dd, *^3^J_H,H_* = 10.6, *^3^J_H,H_* = 7.5 Hz, 1H, *H_14b_*), 2.38 – 2.23 (m, 4H, *H_2_*, *H_11a_*, *H_13_*), 2.10 – 2.00 (m, 3H, *H_9_*, *H_5_*), 2.00 – 1.93 (m, 2H, *H_8_*), δ 1.69 – 1.60 (m, 2H, *H_3_*), 1.56 – 1.49 (m, 1H, *H_11b_*), 1.44 – 1.33 (m, 2H, *H_4_*), 0.88 (s, 9H, C*H_3_* *t*-Bu TBS), 0.87 (s, 9H, C*H_3_* *t*-Bu TBS), 0.05 (s, 3H, C*H_3_* Me-TBS), 0.06 (s, 3H, C*H_3_* Me-TBS), 0.02 (s, 6H, C*H_3_* Me-TBS). Note that OH proton was not detected.

^13^C NMR (101 MHz, CDCl_3_): δ 174.3 (*C_1_*), 130.4 (*C_6_* or *C_7_*), 129.1 (*C_6_* or *C_7_*), 75.9 (*C_10_*), 75.0 (*C_12_*), 62.7 *C_14_*), 51.7 (COO*C*H_3_*)*, 50.3 (*C_13_*), 48.4 (*C_9_*), 44.6 (*C_11_*), 34.1 (*C_2_*), 29.1 (*C_4_*), 27.2 (*C_5_*), 26.0 (7C, *C*H_3_ *t*-Bu-TBS, *C_8_*), 24.8 (*C_3_*), 18.1 (*C_q_* TBS), 18.1 (*C_q_* TBS), -4.1 (*C*H_3_ Me-TBS), -4.3 (*C*H_3_ Me-TBS), -4.5 (*C*H_3_ Me-TBS), -4.7 (*C*H_3_ Me-TBS).

HRMS (TOF ESI+) m/z: calcd for C_27_H_54_O_5_Si_2_Na : 537.3407 [M+Na]^+^, found: 537.3414 [M+Na]^+^.

### Preparation of methyl 8-((1*S*,2*R*,3*R*,5*S*)-3,5-bis((tert-butyldimethylsilyl)oxy)-2-((*E*)-3-oxopent-1-en-1-yl)cyclopentyl)octanoate (**21**)

Pd/C (140 mg, 10% wt) was added to a solution of alkene **19** (1.4 g, 2.72 mmol, 1 equiv.) in EtOAc (28 mL). The mixture was stirred vigorously overnight under H_2_ atmosphere and at room temperature. The mixture was then filtered on Celite®. The solvent was removed under reduced pressure and the crude was purified by silica gel column chromatography using a gradient pentane/Et_2_O from 90/10 to 80/20, to afford alcohol **S9** (1.41 g, 2.72 mmol, quantitative) as a colourless oil.

Rf = 0.5 (cyclohexane/EtOAc: 7/3).

IR (neat): ν = 2928, 2856 (CH), 1742 (C=O) cm^-1^.

^1^H NMR (400 MHz, CDCl_3_): δ 4.04 (q, *^3^J_H,H_* = 6.4 Hz, 1H, *H_12_*), 3.80 (q, *^3^J_H,H_* = 5.5 Hz, 1H, *H_10_*), 3.74 – 3.68 (m, 1H, *H_14a_*), 3.66 (s, 3H, COOC*H_3_*), 3.63 – 3.55 (m, 1H, *H_14b_*), 2.37 – 2.28 (m, 3H, *H_2_*, *H_13_*), 2.27 – 2.20 (m, 1H, *C_11a_*), 2.02 – 1.94 (m, 1H, *H_9_*), 1.71 (br, 1H, OH), 1.66 – 1.59 (m, 2H, *H_3_*), 1.51 (dt, *^2^J_H,H_* = 13.2 Hz, *^3^J_H,H_* = 5.9 Hz, 1H, *H_11b_*), 1.39 – 1.06 (m, 10H, *H_4_*, *H_5_*, *H_6_*, *H_7_*, *H_8_*), 0.87 (s, 9H, C*H_3_* *t*-Bu TBS), 0.88 (s, 9H, C*H_3_* *t*-Bu TBS), 0.05 (s, 3H, C*H_3_* TBS), 0.05 (s, 3H, C*H_3_* TBS), 0.03 (s, 6H, C*H_3_* TBS). Note that OH proton was not detected.

^13^C NMR (101 MHz, CDCl_3_): δ 174.5 (*C_1_*), 76.3 (*C_10_*), 75.2 (*C_12_*), 62.8 (*C_14_*), 51.6 (COO*C*H_3_), 50.5 (*C_13_*), 47.8 (*C_9_*), 44.7 (*C_11_*), 34.2 (*C_2_*), 29.8 (*C_4_* or *C_5_* or *C_6_* or *C_7_*), 29.3 (*C_4_* or *C_5_* or *C_6_* or *C_7_*), 29.2 (*C_4_* or *C_5_* or *C_6_* or *C_7_*), 28.4 (*C_4_* or *C_5_* or *C_6_* or *C_7_*), 28.1 (*C_8_*), 26.0 (6C, *C*H_3_ *t*-Bu-TBS), 25.0 (*C_3_*), 18.1 (*C_q_* TBS), 18.0 (*C_q_* TBS), -4.1 (*C*H_3_ Me-TBS), -4.2 (*C*H_3_ Me-TBS), -4.6 (*C*H_3_ Me-TBS), -4.7 (*C*H_3_ Me-TBS).

HRMS (TOF ESI+) m/z: calcd for C_27_H_57_O_5_Si_2_ : 517.3739 [M+H]^+^, found: 517.3732 [M+H]^+^

[α]_D_^20^ = + 12.2 (8 mg/mL EtOH).

Dess-Martin periodinane (1.72 g, 4.10 mmol, 1.5 equiv.) was added to a solution of alcohol **S9** (1.4 g, 2.72 mmol, 1 equiv.) in 30 mL CH_2_Cl_2_ (technical grade). The mixture was stirred at room temperature for 2 hours. A 10% aqueous solution of Na_2_S_2_O_3_/NaHCO_3_ (100 mL) was then added and the mixture was stirred vigorously at room temperature for 2 hours. The crude was extracted with Et_2_O (3 x 100 mL), the combined organic layers were washed with a 10% aqueous solution of Na_2_S_2_O_3_/NaHCO_3_ (100 mL) and brine, dried over MgSO_4_, filtered and concentrated under reduced pressure. The crude aldehyde **S10** was obtained as a pale-yellow oil and engaged without further purification on the next step.

Rf = 0.43 (cyclohexane/EtOAc: 9/0).

^1^H NMR (400 MHz, CDCl_3_): δ 9.84 (d, ^3^*J*_H,H_ = 2.3 Hz, 1H), 4.48 (dt, ^3^*J*_H,H_ = 7.4, 5.2 Hz, 1H), 3.77 (q, ^3^*J*_H,H_ = 6.6 Hz, 1H), 3.66 (s, 2H), 3.02 (ddd, ^3^*J*_H,H_ = 8.6 Hz, ^3^*J*_H,H_ = 4.4 Hz, ^4^*J*_H,H_ = 2.3 Hz, 1H), 2.37 – 2.26 (m, 2H), 1.65 – 1.52 (m, 4H), 1.39 – 1.20 (m, 12H), 0.88 (s, 9H), 0.86 (s, 9H), 0.03 (s, 9H), 0.02 (s, 3H).

A solution of NaHMDS (2 M in THF, 2.7 mL, 5.40 mmol, 2 equiv.) was slowly added at 0 °C to a solution of diethyl (2-oxobutyl)phosphonate (1.15 mL, 5.94 mmol, 2.2 equiv.) in 100 mL anhydrous THF. The mixture was stirred at 0 °C for 30 minutes and a cold solution of aldehyde **S10** (2.70 mmol, 1 equiv.) in 50 mL anhydrous THF was added. The mixture was then stirred at 0 °C for 30 minutes and overnight at room temperature. The reaction was then quenched by the addition of saturated aqueous NH_4_Cl (100 mL). The mixture was extracted with Et_2_O (3 x 100 mL), the combined organic layers were washed with brine, dried over MgSO_4_ and concentrated under reduced pressure. The crude was then purified by silica gel column chromatography using a gradient pentane/Et_2_O, from 95/5 to 90/10, to afford enone **21** (1.18 g, 2.07 mmol, 77% over 2 steps) as a colourless oil.

Rf = 0.35 (cyclohexane/EtOAc: 9/1).

IR (neat): ν = 2929, 2856 (CH), 1741 (C=O), 1677 (C=O), 1628 (C=C) cm^-1^.

^1^H NMR (400 MHz, CDCl_3_): δ 6.64 (dd, ^3^*J*_H,H_ = 15.7 Hz, ^3^*J*_H,H_ = 10.1 Hz, 1H, *H_14_*), 6.15 (d, ^3^*J*_H,H_ = 15.7 Hz, 1H, *H_15_*), 3.94 (dt, ^3^*J*_H,H_ = 6.9 Hz, ^3^*J*_H,H_ = 5.0 Hz, 1H, *H_12_*), 3.83 (q, ^3^*J*_H,H_ = 6.4 Hz, 1H, *H_10_*), 3.66 (s, 3H, COOC*H_3_*), 2.79 – 2.71 (m, 1H, *H_13_*), 2.55 (q, ^3^*J*_H,H_ = 7.3 Hz, 2H, *H_17_*), 2.35 (dt, ^2^*J*_H,H_ = 14.0 Hz, ^3^*J*_H,H_ = 7.1 Hz, 1H, *H_11a_*), 2.29 (t, ^3^*J*_H,H_ = 7.6 Hz, 2H, *H_2_*), 2.16 – 2.04 (m, 1H, *H_9_*), 1.65 – 1.58 (m, 2H, *H_3_*), 1.56 – 1.53 (m, 1H, *H_11b_*), 1.34 – 1.15 (m, 10H, *H_4_*, *H_5_*, *H_6_*, *H_7_*, *H_8_*), 1.11 (t, ^3^*J*_H,H_ = 7.3 Hz, 3H, *H_18_*), 0.88 (s, 9H, C*H_3_* *t*-Bu TBS), 0.85 (s, 9H, C*H_3_* *t*-Bu TBS), 0.03 (s, 6H, C*H_3_* Me-TBS), 0.00 (s, 3H, C*H_3_* Me-TBS), -0.01 (s, 3H, C*H_3_* Me-TBS).

^13^C NMR (101 MHz, CDCl_3_): δ 200.8 (*C_16_*), 174.5 (*C_1_*), 145.7 (*C_14_*), 131.0 (*C_15_*), 76.2 (*C_10_*), 75.5 (*C_12_*), 53.2 (*C_13_*), 51.6 (COO*C*H_3_), 50.1 (*C_9_*), 44.6 (*C_11_*), 34.2 (*C_2_* or *C_17_*), 34.1 (*C_2_* or *C_17_*), 29.7 (*C_4_* or *C_5_* or *C_6_* or *C_7_*), 29.3 (*C_4_* or *C_5_* or *C_6_* or *C_7_*), 29.2 (*C_4_* or *C_5_* or *C_6_* or *C_7_*), 28.9 (*C_4_* or *C_5_* or *C_6_* or *C_7_*), 28.1 (*C_8_*), 26.0 (6C, *C*H_3_ *t*-Bu TBS), 25.1 (*C_3_*), 18.1 (2C, *C_q_* TBS), 8.3 (*C_18_*), -4.2 (*C*H_3_ Me-TBS), -4.5 (*C*H_3_ Me-TBS), -4.5 (*C*H_3_ Me-TBS), -4.6 (*C*H_3_ Me-TBS).

HRMS (TOF ESI+) m/z: calcd for C_31_H_61_O_5_Si_2_ : 569.4052 [M+H]^+^, found: 569.4050.

[α]_D_^20^ = + 12.3 (8 mg/mL EtOH).

### Preparation of 3,4-bis((tert-butyldimethylsilyl)oxy)phenethyl 8-((1*S*,2*R*,3*R*,5*S*)-3,5-bis((tert-butyldimethylsilyl)oxy)-2-((*E*)-3-oxopent-1-en-1-yl)cyclopentyl)octanoate (**23**)

A solution of LiOH.H_2_O (221 mg, 5.27 mmol, 6 equiv.) in distilled water (10 mL) was added to a solution of **21** (500 mg, 0.88 mmol, 1 equiv.) in THF (10 mL). The mixture was stirred overnight at 40 °C and then acidified by the addition of aqueous HCl (1M, 20 mL) until pH = 1. The mixture was extracted by EtOAc (3 x 20 mL), the combined organic layers were washed with HCl 1M (20 mL) and brine (20 mL), dried over MgSO_4_ and the solvent was removed under reduced pressure. The crude acid **S11** was obtained as a yellow oil and directly engaged in the next step (yield calculated on 2 steps).

DCC (219 mg, 1.06 mmol, 1.2 equiv.), DMAP (65 mg, 0.53 mmol, 0.6 equiv.) and **22** (341 mg, 0.88 mmol, 1 equiv., in 5 mL anhydrous CH_2_Cl_2_) were successively added to a solution of acid **S11** (500 mg, 0.88 mmol, 1 equiv.) in 15 mL anhydrous CH_2_Cl_2_. The mixture was stirred for 18 hours at room temperature and was then filtered on Celite®. The filtrate was concentrated under reduced pressure and the crude was purified by silica gel column chromatography using pentane/Et_2_O: 90/10 as eluent to afford the ester **23** (562 mg, 0.61 mmol, 69% over 2 steps) as a colourless oil.

Rf = 0.75 (cyclohexane/EtOAc: 8/2).

IR (neat): ν = 2929, 2857 (CH), 1738 (C=O), 1677 (C=O), 1628 (C=C), 1511 (C=C Ar) cm^-1^.

^1^H NMR (400 MHz, CDCl_3_): δ 6.74 (d, ^3^*J*_H,H_ = 8.1 Hz, 1H, *H_Ar_*), 6.70 – 6.59 (m, 3H, *H_14_*, *H_Ar_*), 6.15 (d, ^3^*J*_H,H_ = 15.7 Hz, 1H, *H_15_*), 4.21 (t, ^3^*J*_H,H_ = 7.2 Hz, 2H, *H_2’_*), 3.94 (dt, *^3^J_H,H_* = 6.9 Hz, ^3^*J*_H,H_ = 5.0 Hz, 1H, *H_12_*), 3.84 (q, ^3^*J*_H,H_ = 6.4 Hz, 1H, *H_10_*), 2.79 (t, ^3^*J*_H,H_ = 7.2 Hz, 2H, *H_3’_*), 2.77 – 2.71 (m, 1H, *H_13_*), 2.54 (q, ^3^*J*_H,H_ = 7.3 Hz, 2H, *H_17_*), 2.35 (dt, ^2^*J*_H,H_ = 14.0 Hz, ^3^*J*_H,H_ = 7.1 Hz, 1H, *H_11a_*), 2.26 (t, ^3^*J*_H,H_ = 7.6 Hz, 2H, *H_2_*), 2.15 – 2.05 (m, 1H, *H_9_*), 1.61 – 1.53 (m, 3H, *H_3_*, *H_11b_*), 1.31 – 1.20 (m, 10H, *H_4_*, *H_5_*, *H_6_*, *H_7_*, *H_8_*), 1.10 (t, ^3^*J*_H,H_ = 7.3 Hz, 3H, *H_18_*), 0.97 (s, 9H, C*H_3_* *t*-Bu TBS), 0.98 (s, 9H, C*H_3_* *t*-Bu TBS), 0.88 (s, 9H, C*H_3_ t*-Bu TBS), 0.85 (s, 9H, C*H_3_ t*-Bu TBS), 0.18 (s, 6H, C*H_3_* Me-TBS), 0.18 (s, 6H, C*H_3_* Me-TBS), 0.03 (s, 6H, C*H_3_* Me-TBS), 0.00 (s, 3H, C*H_3_* Me-TBS) -0.01 (s, 3H, C*H_3_* Me-TBS).

^13^C NMR (101 MHz, CDCl_3_): δ 200.7 (*C_16_*), 173.9 (*C_1_*), 146.8 (*C_Ar_*), 145.6 (*C_14_*), 145.6 (*C_Ar_*), 131.0 (2C, *C_15_*, *C_Ar_*), 121.9 (*C*H*_Ar_*), 121.9 (*C*H*_Ar_*), 121.1 (*C*H*_Ar_*), 76.2 (*C_10_*), 75.5 (*C_12_*), 65.1 (*C_2’_*), 53.2 (*C_13_*), 50.1 (*C_9_*), 44.6 (*C_11_*), 34.6 (*C_3’_*), 34.5 (*C_2_*), 34.1 (*C_17_*), 29.8 (*C_4_* or *C_5_* or *C_6_* or *C_7_*), 29.3 (*C_4_* or *C_5_* or *C_6_* or *C_7_*), 29.3 (*C_4_* or *C_5_* or *C_6_* or *C_7_*), 28.9 (*C_8_*), 28.2 (*C_4_* or *C_5_* or *C_6_* or *C_7_*), 26.1 (6C, *C*H_3_ *t*-Bu TBS), 26.0 (3C, *C*H_3_ *t*-Bu TBS), 25.9 (3C, *C*H_3_ *t*-Bu TBS), 25.1 (*C_3_*), 18.6 (2C, *C_q_* TBS), 18.1 (2C, *C_q_* TBS), 8.3 (*C_18_*), -3.9 (4C, *C*H_3_ Me-TBS), -4.2 (*C*H_3_ Me-TBS), -4.4 (*C*H_3_ Me-TBS), -4.5 (*C*H_3_ Me-TBS), -4.6 (*C*H_3_ Me-TBS).

HRMS (TOF ESI+) m/z: calcd for C_50_H_95_O_7_Si_4_ : 919.6149 [M+H]^+^, found: 919.6153 [M+H]^+^.

[α]_D_^20^ = + 7.6 (8 mg/mL EtOH)

### Preparation of 3,4-dihydroxyphenethyl 8-((1*S*,2*R*,3*R*,5*S*)-3,5-dihydroxy-2-((*S*,*E*)-3-hydroxypent-1-en-1-yl)cyclopentyl)octanoate – 16-F_1t_-PhytoP-HT (**1**)

BH_3_.Me_2_S (2M in THF, 217 µL, 0.43 mmol, 2 equiv.) was added at 0 °C to a solution of (*R*)-2-methyl-CBS-oxazaborolidine (1M in toluene, 522 µL, 0.52 mmol, 2.2 equiv.) in anhydrous THF (5 mL). The mixture was stirred at 0 °C for 15 minutes and a cold solution of **23** (200 mg, 0.22 mmol, 1 equiv., solution in 4 mL anhydrous THF) was added. The mixture was stirred at 0 °C for 30 minutes, then quenched by the addition of MeOH (8 mL) and the solvents were removed under reduced pressure. The crude was then purified by silica gel column chromatography using pentane/Et_2_O: 90/10 as eluent to afford compound **S12** (192 mg, yield calculated on 2 steps) as a colourless oil.

Rf = 0.5 (cyclohexane/EtOAc: 9/1).

IR (neat): ν = 3478 (OH), 2929, 2857 (CH), 1738 (C=O), 1511 (C=C Ar) cm^-1^.

^1^H NMR (400 MHz, CDCl_3_): δ 6.74 (d, ^3^*J*_H,H_ = 8.1 Hz, 1H, *H_Ar_*), 6.68 (d, ^3^*J*_H,H_ = 2.1 Hz, 1H, *H_Ar_*), 6.64 (dd, ^3^*J*_H,H_ = 8.1, 2.3 Hz, 1H, *H_Ar_*), 5.49 (dd, ^3^*J*_H,H_ = 15.3 Hz, ^3^*J*_H,H_ = 6.6 Hz, 1H, *H_15_*), 5.37 (dd, ^3^*J*_H,H_ = 15.4 Hz, ^3^*J*_H,H_ = 9.5 Hz, 1H, *H_14_*), 4.21 (t, ^3^*J*_H,H_ = 7.2 Hz, 2H, *H_2’_*), 4.03 – 3.94 (m, 1H, *H_16_*), 3.87 (dt, ^3^*J*_H,H_ = 6.8 Hz, ^3^*J*_H,H_ = 4.5 Hz, 1H, *H_12_*), 3.76 (q, ^3^*J*_H,H_ = 7.2 Hz, 1H, *H_10_*), 2.80 (t, ^3^*J*_H,H_ = 7.2 Hz, 2H, *H_3’_*), 2.61 – 2.53 (m, 1H, *H_13_*), 2.36 – 2.23 (m, 3H, including t, ^3^*J*_H,H_ = 7.6 Hz, *H_11a_*, *H_2_*), 2.08 – 1.97 (m, 1H, *H_9_*), 1.63 – 1.47 (m, 5H, *H_3_*, *H_11b_*, *H_17_*), 1.39 (d, ^3^*J*_H,H_ = 3.9 Hz, 1H, O*H*), 1.33 – 1.12 (m, 10H, *H_4_*, *H_5_*, *H_6_*, *H_7_*, *H_8_*), 0.98 (s, 9H, C*H_3_* *t*-Bu TBS), 0.98 (s, 9H, C*H_3_* *t*-Bu TBS), 0.90 (t, ^3^*J*_H,H_ = 7.4 Hz, 3H, *H_18_*), 0.88 (s, 9H, C*H_3_* *t*-Bu TBS), 0.87 (s, 9H, C*H_3_* *t*-Bu TBS), 0.18 (s, 6H, C*H_3_* Me-TBS), 0.18 (s, 6H, C*H_3_* Me-TBS), 0.03 (s, 6H, C*H_3_* Me-TBS), 0.02 (s, 6H, C*H_3_* Me-TBS).

^13^C NMR (101 MHz, CDCl_3_): δ 174.0 (*C_1_*), 146.8 (*C_Ar_*), 145.6 (*C_Ar_*), 134.9 (*C_15_*), 131.0 (*C_Ar_*), 130.5 (*C_14_*), 121.93 (*C*H*_Ar_*), 121.87 (*C*H*_Ar_*), 121.1 (*CH_Ar_*), 76.6 (*C_10_*), 76.3 (*C_12_*), 74.6 (*C_16_*), 65.1 (*C_2’_*), 53.0 (*C_13_*), 49.3 (*C_9_*), 44.6 (*C_11_*), 34.6 (*C_2_* or *C_3’_*), 34.5 (*C_2_* or *C_3’_*), 30.4 (*C_17_*), 29.9 (*C_4_* or *C_5_* or *C_6_* or *C_7_*), 29.4 (*C_4_* or *C_5_* or *C_6_* or *C_7_*), 29.3 (*C_4_* or *C_5_* or *C_6_* or *C_7_*), 28.8 (*C_8_*), 28.0 (*C_4_* or *C_5_* or *C_6_* or *C_7_*), 26.1 (6C, *C*H_3_ *t*-Bu TBS), 26.0 (6C, *C*H_3_ *t*-Bu TBS), 25.1 (*C_3_*), 18.6 (2C, *C_q_* TBS), 18.20 (*C_q_* TBS), 18.17 (*C_q_* TBS), -3.9 (4C, *C*H_3_ Me-TBS), -4.2 (*C*H_3_ Me-TBS), -4.4 (*C*H_3_ Me-TBS), -4.4 (*C*H_3_ Me-TBS), -4.6 (*C*H_3_ Me-TBS).

HRMS (TOF ESI+) m/z: calcd for C_50_H_100_NO_7_Si_4_ : 938.6571 [M+NH_4_]^+^, found: 938.6573 [M+NH_4_]^+^.

In a Teflon® flask, Et_3_N.3HF (145 µL, 0.89 mmol, 6 equiv.) was added to a solution of **S12** (137 mg, 0.15 mmol, 1 equiv.) in 3 mL anhydrous THF and the mixture was stirred at room temperature for 12 hours. At this point, 6 more equivalents of Et_3_N.3HF (145 µL) were added and the mixture was stirred at room temperature for another 12 hours. The mixture was then treated with a dropwise addition of a saturated aqueous solution of NaHCO_3_ (until gas formation is over) and 5 mL of brine were added to facilitate the extraction. The mixture was extracted by EtOAc (3 x 10 mL), the combined organic layers were washed with brine, dried over MgSO_4_ and concentrated under reduced pressure. The crude was then purified by silica gel column chromatography using a gradient EtOAc/MeOH from100/0 to 98/2, to afford 16-F_1t_-PhytoP-HT **1** (30 mg, 0.07 mmol, 41% over 2 steps) as a colourless oil.

Rf = 0.18 (EtOAc/MeOH: 98/2).

IR (neat): ν = 3325 (OH), 2926, 2854 (CH), 1716 (C=O), 1448 (C=C Ar) cm^-1^.

^1^H NMR (600 MHz, MeOD): δ 6.68 (d, ^3^*J*_H,H_ = 7.9 Hz, 1H, *H_Ar_*), 6.66 (d, ^3^*J*_H,H_ = 2.1 Hz, 1H, *H_Ar_*), 6.53 (dd, ^3^*J*_H,H_ = 8.1 Hz, ^4^*J*_H,H_ = 2.1 Hz, 1H, *H_Ar_*), 5.48 (dd, ^3^*J*_H,H_ = 15.3 Hz, ^3^*J*_H,H_ = 6.9 Hz, 1H, *H_15_*), 5.41 (dd, ^3^*J*_H,H_ = 15.4 Hz, ^3^*J*_H,H_ = 9.4 Hz, 1H, *H_14_*), 4.20 (t, ^3^*J*_H,H_ = 7.0 Hz, 2H, *H_2’_*), 3.94 – 3.88 (m, 2H, *H_16_*, *H_12_*), 3.82 (q, ^3^*J*_H,H_ = 7.5 Hz, 1H, *H_10_*), 2.76 (t, ^3^*J*_H,H_ = 7.0 Hz, 2H, *H_3’_*), 2.67 – 2.61 (m, 1H, *H_13_*), 2.46 (ddd, ^2^*J*_H,H_ = 14.4 Hz, *^3^J_H,H_* = 7.8 Hz, ^3^*J*_H,H_ = 6.9 Hz, 1H, *H_11a_*), 2.28 (t, ^3^*J*_H,H_ = 7.4 Hz, 2H, *H_2_*), 2.05 – 1.98 (m, 1H, *H_9_*), 1.60 – 1.51 (m, 4H, *H_3_*, *H_17_*), 1.50 – 1.43 (m, 1H, *H_11b_*), 1.42 – 1.21 (m, 10H, *H_4_*, *H_5_*, *H_6_*, *H_7_*, *H_8_*), 0.89 (t, ^3^*J*_H,H_ = 7.5 Hz, 3H, *H_18_*).

^13^C NMR (151 MHz, MeOD): δ 175.6 (*C_1_*), 146.3 (*C_Ar_*), 144.9 (*C_Ar_*), 136.4 (*C_15_*), 130.8 (*C_14_*), 121.2 (*C*H*_Ar_*), 117.0 (*C*H_Ar_), 116.3 (*C*H_Ar_), 76.9 (*C_10_*), 76.6 (*C_12_*), 75.2 (*C_16_*), 66.4 (*C_2’_*), 54.2 (*C_13_*), 50.4 (*C_9_*), 43.7 (*C_11_*), 35.5 (*C_3’_*), 35.1 (*C_2_*), 31.3 (*C_17_*), 30.8 (*C_4_* or *C_5_* or *C_6_* or *C_7_*), 30.3 (*C_4_* or *C_5_* or *C_6_* or *C_7_*), 30.1 (*C_4_* or *C_5_* or *C_6_* or *C_7_*), 29.8 (*C_8_*), 29.0 (*C_4_* or *C_5_* or *C_6_* or *C_7_*), 26.0 (*C_3_*), 10.2 (*C_18_*).

HRMS (TOF ESI+) m/z: calcd for C_26_H_39_O_7_ : 463.2701 [M-H]^-^, found: 463.2694 [M-H]^-^

[α]_D_^20^ = + 0.023 (1 mg/mL EtOH).

The diastereomeric ratio (determinated by HPLC, see method in general procedures) is 97:3 in favor of the (*S*)-epimer at the C16 center.

### Preparation of 3,4-dihydroxyphenethyl 8-((1*S*,2*R*,3*R*,5*S*)-3,5-dihydroxy-2-((*R*,*E*)-3-hydroxypent-1-en-1-yl)cyclopentyl)octanoate – 16-*epi*-16-F_1t_-PhytoP-HT (***16-epi-*1**)

The same procedure previously described to access **S12** from **23** was applied from **23** (200 mg, 0.22 mmol, 1 equiv.) using (*S*)-2-methyl-CBS-oxazaborolidine (1M in toluene, 484 µL, 0.48 mmol, 2.2 equiv.) to obtain ***16-epi*-S12** (182 mg, yield calculated on 2 steps) as a colourless oil.

Rf = 0.5 (cyclohexane/EtOAc: 9/1).

IR (neat): ν = 3478 (OH), 2929, 2857 (CH), 1738 (C=O), 1511 (C=C Ar) cm^-1^.

^1^H NMR (600 MHz, CDCl_3_): δ 6.74 (d, ^3^*J*_H,H_ = 8.1 Hz, 1H, *H_Ar_*), 6.68 (d, ^4^*J*_H,H_ = 2.1 Hz, 1H, *H_Ar_*), 6.64 (dd, ^3^*J*_H,H_ = 8.1 Hz, ^4^*J*_H,H_ = 2.2 Hz, 1H, *H_Ar_*), 5.50 (dd, ^3^*J*_H,H_ = 15.4 Hz, ^3^*J*_H,H_ = 6.5 Hz, 1H, *H_15_*), 5.41 (dd, ^3^*J*_H,H_ = 15.0 Hz, ^3^*J*_H,H_ = 10.0 Hz, 1H, *H_14_*), 4.21 (t, ^3^*J*_H,H_ = 7.2 Hz, 2H, *H_2’_*), 4.03 – 3.97 (m, 1H, *H_16_*), 3.89 – 3.83 (m, 1H, *H_12_*), 3.77 (q, ^3^*J*_H,H_ = 7.1 Hz, 1H, *H_10_*), 2.80 (t, ^3^*J*_H,H_ = 7.2 Hz, 2H, *H_3’_*), 2.61 – 2.55 (m, 1H, *H_13_*), 2.31 (dt, ^2^*J*_H,H_ = 14.1 Hz, ^3^*J*_H,H_ = 7.2 Hz, 1H, *H_11a_*), 2.27 (t, ^3^*J*_H,H_ = 7.6 Hz, 2H, *H_2_*), 2.05 – 1.98 (m, 1H, *H_9_*), 1.61 – 1.49 (m, 5H, *H_3_*, *H_11b_*, *H_17_*), 1.43 (d, ^3^*J*_H,H_ = 4.0 Hz, 1H, O*H*), 1.32 – 1.21 (m, 10H, *H_4_*, *H_5_*, *H_6_*, *H_7_*, *H_8_*), 0.98 (s, 9H, C*H_3_* *t*-Bu TBS), 0.98 (s, 9H, C*H_3_* *t*-Bu TBS), 0.91 (t, ^3^*J*_H,H_ = 7.5 Hz, 3H, *H_18_*), 0.88 (s, 9H, C*H_3_* *t*-Bu TBS), 0.86 (s, 9H, C*H_3_* *t*-Bu TBS), 0.18 (s, 6H, C*H_3_* Me-TBS), 0.18 (s, 6H, C*H_3_* Me-TBS), 0.03 (s, 3H, C*H_3_* Me-TBS), 0.02 (s, 3H, C*H_3_* Me-TBS), 0.01 (s, 3H, C*H_3_* Me-TBS), 0.01 (s, 3H, C*H_3_* Me-TBS).

^13^C NMR (151 MHz, CDCl_3_) δ 174.0 (*C_1_*), 146.8 (*C_Ar_*), 145.6 (*C_Ar_*), 134.9 (*C_15_*), 131.0 (*C_Ar_*), 130.2 (*C_14_*), 121.9 (*C*H*_Ar_*), 121.9 (*C*H*_Ar_*), 121.1 (*CH_Ar_*), 76.5 (*C_10_*), 76.3 (*C_12_*), 74.3 (*C_16_*), 65.1 (*C_2’_*), 52.8 (*C_13_*), 49.3 (*C_9_*), 44.6 (*C_11_*), 34.6 (*C_2_* or *C_3’_*), 34.5 (*C_2_* or *C_3’_*), 30.4 (*C_17_*), 29.9 (*C_4_* or *C_5_* or *C_6_* or *C_7_*), 29.4 (*C_4_* or *C_5_* or *C_6_* or *C_7_*), 29.3 (*C_4_* or *C_5_* or *C_6_* or *C_7_*), 28.7 (*C_8_*), 28.0 (*C_4_* or *C_5_* or *C_6_* or *C_7_*), 26.1 (6C, *C*H_3_ *t*-Bu TBS), 26.0 (6C, *C*H_3_ *t*-Bu TBS), 25.1 (*C_3_*), 18.6 (2C, *C_q_* TBS), 18.2 (*C_q_* TBS), 18.2 (*C_q_* TBS), 9.8 (*C_18_*), -3.9 (2C, *C*H_3_ Me-TBS), -4.0 (2C, *C*H_3_ Me-TBS), -4.2 (*C*H_3_ Me-TBS), -4.4 (2C, *C*H_3_ Me-TBS), -4.6 (*C*H_3_ Me-TBS).

HRMS (TOF ESI+) m/z: calcd for C_50_H_100_NO_7_Si_4_ : 938.6571 [M+NH_4_]^+^, found: 938.6594 [M+NH_4_]^+^.

The same procedure previously described to access **1** from **S12** was applied from ***16-epi*-S12** (130 mg, 0.14 mmol, 1 equiv.) using Et_3_N.3HF (138 µL, 0.85 mmol, 6 equiv.) to afford pure ***16-epi-*1** as a colourless oil (38 mg, 0.08 mmol, 53% over 2 steps).

Rf = 0.18 (EtOAc/MeOH: 98/2).

IR (neat): ν = 3337 (OH), 2926, 2854 (CH), 1716 (C=O), 1528, 1453 (C=C Ar) cm^-1^.

^1^H NMR (400 MHz, MeOD): δ 6.68 (d, ^3^*J*_H,H_ = 7.9 Hz, 1H, *H_Ar_*), 6.66 (d, ^3^*J*_H,H_ = 2.1 Hz, 1H, *H_Ar_*), 6.53 (dd, ^3^*J*_H,H_ = 8.1 Hz, ^4^*J*_H,H_ = 2.1 Hz, 1H, *H_Ar_*), 5.48 (dd, ^3^*J*_H,H_ = 15.3 Hz, ^3^*J*_H,H_ = 6.9 Hz, 1H, *H_15_*), 5.41 (dd, ^3^*J*_H,H_ = 15.4 Hz, ^3^*J*_H,H_ = 9.4 Hz, 1H, *H_14_*), 4.20 (t, ^3^*J*_H,H_ = 7.0 Hz, 2H, *H_2’_*), 3.94 – 3.88 (m, 2H, *H_16_*, *H_12_*), 3.82 (q, ^3^*J*_H,H_ = 7.5 Hz, 1H, *H_10_*), 2.76 (t, ^3^*J*_H,H_ = 7.0 Hz, 2H, *H_3’_*), 2.67 – 2.61 (m, 1H, *H_13_*), 2.46 (ddd, ^2^*J*_H,H_ = 14.4 Hz, ^3^*J*_H,H_ = 7.8 Hz, ^3^*J*_H,H_ = 6.9 Hz, 1H, *H_11a_*), 2.28 (t, ^3^*J*_H,H_ = 7.4 Hz, 2H, *H_2_*), 2.05 – 1.98 (m, 1H, *H_9_*), 1.60 – 1.51 (m, 4H, *H_3_*, *H_17_*), 1.50 – 1.43 (m, 1H, *H_11b_*), 1.42 – 1.21 (m, 10H, *H_4_*, *H_5_*, *H_6_*, *H_7_*, *H_8_*), 0.89 (t, ^3^*J*_H,H_ = 7.5 Hz, 3H, *H_18_*).

^13^C NMR (151 MHz, MeOD): δ 175.6 (*C_1_*), 146.3 (*C_Ar_*), 144.9 (*C_Ar_*), 136.4 (*C_15_*), 130.8 (*C_14_*), 121.2 (*C*H*_Ar_*), 117.0 (*C*H_Ar_), 116.3 (*C*H_Ar_), 76.9 (*C_10_*), 76.6 (*C_12_*), 75.2 (*C_16_*), 66.4 (*C_2’_*), 54.2 (*C_13_*), 50.4 (*C_9_*), 43.7 (*C_11_*), 35.5 (*C_3’_*), 35.1 (*C_2_*), 31.3 (*C_17_*), 30.8 (*C_4_* or *C_5_* or *C_6_* or *C_7_*), 30.3 (*C_4_* or *C_5_* or *C_6_* or *C_7_*), 30.1 (*C_4_* or *C_5_* or *C_6_* or *C_7_*), 29.8 (*C_8_*), 29.0 (*C_4_* or *C_5_* or *C_6_* or *C_7_*), 26.0 (*C_3_*), 10.2 (*C_18_*).

HRMS (TOF ESI+) m/z: calcd for C_26_H_39_O_7_ : 463.2701 [M-H]^-^, found: 463.2694 [M-H]^-^

[α]_D_^20^ = + 0.013 (1 mg/mL EtOH).

The diastereomeric ratio (determinated by HPLC) is superior at 99:1 of the (*R*)-epimer at the C16 center.

## Towards the synthesis of the 16-F_1t_-PhytoP and its C16-epimer

### Preparation of 8-((1*S*,2*R*,3*R*,5*S*)-3,5-dihydroxy-2-((*S*,*E*)-3-hydroxypent-1-en-1-yl)cyclopentyl) octanoic acid – 16-F_1t_-PhytoP (**S13**)

BH_3_.Me_2_S (2M in THF, 330 µL, 0.66 mmol, 2.5 equiv.) was added at 0 °C to a solution of (*R*)-2-methyl-CBS-oxazaborolidine (1M in toluene, 790 µL, 0.79 mmol, 3 equiv.) in anhydrous THF (8 mL). The mixture was stirred at 0 °C for 15 minutes and a cold solution of **21** (150 mg, 0.26 mmol, 1 equiv., solution in 5 mL anhydrous THF) was added. The mixture was stirred at 0 °C for 30 minutes, then quenched by the addition of MeOH (5 mL) and the solvents were removed under reduced pressure. The crude was then purified by silica gel column chromatography using pentane/Et_2_O 90/10 as eluent.

In a Teflon® flask, Et_3_N.3HF (60 µL, 0.37 mmol, 3 equiv.) was added to a solution of half of the previously prepared compound (70 mg, 0.12 mmol, 1 equiv.) in 2.5 mL anhydrous THF and the mixture was stirred at room temperature for 12 hours. At this point, 3 more equivalents of Et_3_N.3HF (60 µL) were added and the mixture was stirred at room temperature for another 12 hours. The mixture was then treated with a dropwise addition of a saturated aqueous solution of NaHCO_3_ (until gas formation is over) and 5 mL brine were added to facilitate the extraction. The crude was extracted by EtOAc (3 x 10 mL), the combined organic layers were washed with brine, dried over MgSO_4_ and concentrated under reduced pressure. The crude was then purified by silica gel column chromatography using 100% EtOAc as eluent.

To a solution of the previously prepared compound (35 mg, 0.10 mmol, 1 equiv.) in THF (1 mL) and water (1 mL) was added LiOH.H_2_O (26 mg, 0.61 mmol, 6 equiv.) and the reaction was stirred at room temperature overnight. The reaction was quenched by slow addition of an aqueous solution of HCl 1M, until acidic pH, and the aqueous phase was extracted with EtOAc (3 x 10 mL), washed with brine, dried over MgSO_4_ and concentrated under vacuum. The crude was then purified by silica gel column chromatography using EtOAc/MeOH: 95/5 as eluent to afford the 16-F_1t_-PhytoP **S13** (21 mg, 0.06 mmol, 39% over 3 steps) as a colorless oil.

Rf = 0.1 (EtOAc/MeOH: 98/2).

^1^H NMR (400 MHz, MeOD): δ 5.49 (dd, ^3^*J*_H,H_ = 15.3 Hz, ^3^*J*_H,H_ = 6.6 Hz, 1H, *H_14_*), 5.41 (dd, ^3^*J*_H,H_ = 15.5 Hz, ^3^*J*_H,H_ = 9.2 Hz, 1H, *H_15_*), 3.95 – 3.88 (m, 2H, *H_16_*, *H_12_*), 3.82 (q, ^3^*J*_H,H_ = 7.5 Hz, 1H, *H_10_*), 2.62 (td, ^3^*J*_H,H_ = 8.4 Hz, ^3^*J*_H,H_ = 3.7 Hz, 1H, *H_13_*), 2.51 – 2.42 (m, 1H, *H_11a_*), 2.27 (t, ^3^*J*_H,H_ = 7.4 Hz, 2H, *H_2_*), 2.07 – 1.97 (m, 1H, *H_9_*), 1.65 – 1.43 (m, 5H, *H_3_*, *H_17_*, *H_11b_*), 1.42 – 1.25 (m, 10H, *H_4_*, *H_5_*, *H_6_*, *H_7_*, *H_8_*), 0.90 (t, ^3^*J*_H,H_ = 7.4 Hz, 3H, *H_18_*).

^13^C NMR (101 MHz, MeOD): δ 177.8 (*C_1_*), 136.4 (*C_15_*), 130.8 (*C_14_*), 76.9 (*C_10_*), 76.6 (*C_12_*), 75.2 (*C_16_*), 54.2 (*C_13_*), 50.4 (*C_9_*), 43.7 (*C_11_*), 35.0 (*C_2_*), 31.3 (*C_17_*), 30.8 (*C_4_* or *C_5_* or *C_6_* or *C_7_*), 30.4 (*C_4_* or *C_5_* or *C_6_* or *C_7_*), 30.2 (*C_4_* or *C_5_* or *C_6_* or *C_7_*), 29.8 (*C_8_*), 29.1 (*C_4_* or *C_5_* or *C_6_* or *C_7_*), 26.1 (*C_3_*), 10.2 (*C_18_*).

[α]_D_^20^ = + 25 (c = 0,12, MeOH).

### Preparation of 8-((1*S*,2*R*,3*R*,5*S*)-3,5-dihydroxy-2-((*R*,*E*)-3-hydroxypent-1-en-1-yl)cyclopentyl)octanoic acid – 16-epi-16-F1t-PhytoP (**16-*epi*-S13**)

BH_3_.Me_2_S (2M in THF, 260 µL, 0.51 mmol, 1.25 equiv.) was added at 0 °C to a solution of (*S*)-2-methyl-CBS-oxazaborolidine (1M in toluene, 620 µL, 0.62 mmol, 1.5 equiv.) in anhydrous THF (5 mL). The mixture was stirred at 0 °C for 5 minutes and a cold solution of **21** (234 mg, 0.41 mmol, 1 equiv., solution in 4 mL anhydrous THF) was added. The mixture was stirred at 0 °C for 30 minutes, then quenched by the addition of MeOH (1 mL) and the solvents were removed under reduced pressure. The crude was then purified by silica gel column chromatography using pentane/Et_2_O: 90/10 as eluent.

To a solution of the previously prepared compound (190 mg, 0.325 mmol, 1 equiv.) in THF (20 mL) and MeOH (10 mL) at 0 °C was added HCl 0.5N/MeOH (6.5 mL, 3.25 mmol, 10 equiv.) and the reaction was stirred at room temperature for 2.5 hours. The reaction was quenched by the addition of NaHCO_3_ saturated solution. The aqueous phase was extracted with EtOAc (3 x 20 mL), washed with brine, dried over MgSO_4_ and concentrated under vacuum. The crude product was purified by silica gel column chromatography using 100% EtOAc as eluent.

To a solution of the previously prepared compound (110 mg, 0.31 mmol, 1 equiv.) in THF (4 mL) and water (4 mL) was added LiOH.H_2_O (78 mg, 1.85 mmol, 6 equiv.) and the reaction was stirred at room temperature for 3 hours. The reaction was quenched by slow addition of an aqueous solution of NaHSO_4_ (1M), until acidic pH, and the aqueous phase was extracted with EtOAc (3 x 2 mL), washed with brine, dried over MgSO_4_ and concentrated under vacuum. The crude product was purified by silica gel column chromatography using EtOAc/MeOH: 80/20 to afford the 16-*epi*-16-F_1t_-PhytoP **16-*epi*-S13** (46 mg, 0.14 mmol, 34% over 3 steps) as a colorless oil.

Rf = 0.3 (EtOAc/MeOH: 8/2).

^1^H NMR (300 MHz, MeOD): δ 5.53 (dd, ^3^*J*_H,H_ = 15.3 Hz, ^3^*J*_H,H_ = 5.5 Hz, 1H, *H_14_*), 5.45 (dd, ^3^*J*_H,H_ = 15.5 Hz, ^3^*J*_H,H_ = 8.5 Hz, 1H, *H_15_*), 3.99 – 3.88 (m, 2H, *H_16_*, *H_12_*), 3.83 (q, ^3^*J*_H,H_ = 7.4 Hz, 1H, *H_10_*), 2.65 (td, ^3^*J*_H,H_ = 7.9 Hz, ^3^*J*_H,H_ = 3.9 Hz, 1H, *H_13_*), 2.46 (dt, ^2^*J*_H,H_ = 14.5 Hz, ^3^*J*_H,H_ = 7.1 Hz, 1H, *H_11a_*), 2.27 (t, ^3^*J*_H,H_ = 7.4 Hz, 2H, *H_2_*), 2.07 – 1.95 (m, 1H, *H_9_*), 1.65 – 1.45 (m, 5H, *H_3_*, *H_17_*, *H_11b_*), 1.43 – 1.26 (m, 10H, *H_4_*, *H_5_*, *H_6_*, *H_7_*, *H_8_*), 0.91 (t, ^3^*J*_H,H_ = 7.4 Hz, 3H, *H_18_*).

^13^C NMR (75 MHz, MeOD): δ 177.7 (*C_1_*), 136.2 (*C_15_*), 129.8 (*C_14_*), 76.8 (*C_10_*), 76.5 (*C_12_*), 74.6 (*C_16_*), 53.9 (*C_13_*), 50.4 (*C_9_*), 43.7 (*C_11_*), 35.0 (*C_2_*), 31.3 (*C_17_*), 30.8 (*C_4_* or *C_5_* or *C_6_* or *C_7_*), 30.4 (*C_4_* or *C_5_* or *C_6_* or *C_7_*), 30.2 (*C_4_* or *C_5_* or *C_6_* or *C_7_*), 29.7 (*C_8_*), 29.1 (*C_4_* or *C_5_* or *C_6_* or *C_7_*), 26.1 (*C_3_*), 10.2 (*C_18_*).

[α]_D_^20^ = + 18.0 (c = 0,45, MeOH).

## Towards the synthesis of the 9-F_1t_-PhytoP-HT and its C9-epimer

### Preparation of (((1*S*,3*R*,4*R*,5*R*)-4-ethyl-5-(((triethylsilyl)oxy)methyl)cyclopentane-1,3-diyl)bis(oxy))bis(tert-butyldimethylsilane) (**4**)

TsCl (606 mg, 3.18 mmol, 1.5 equiv.), DMAP (24 mg, 0.2 mmol, 0.1 equiv.) and distilled Et_3_N (443 µL, 3.18 mmol, 1.5 equiv.) were successively added to a solution of **6** (1.1 g, 2.12 mmol, 1 equiv.) in 22 mL anhydrous CH_2_Cl_2_. The mixture was stirred for 16 hours at room temperature and was then treated by the addition of water (20 mL). The mixture was extracted by Et_2_O (2x 20 mL), the combined organic layers were washed with brine, dried over MgSO_4,_ and concentrated under reduced pressure. The crude was then purified by rapid silica gel column chromatography using a gradient pentane/Et_2_O from 98/2 to 95/5, to afford the tosylate **S14** (1.0 g) as a colourless oil containing approximately 17% of TsCl (estimated with H_Ar_ integration). Note that 230 mg of starting material **6** were recovered.

Rf = 0.33 (cyclohexane/EtOAc: 96/4).

^1^H NMR (400 MHz, CDCl_3_): δ 7.78 (d, ^3^*J*_H,H_ = 8.4 Hz, 2H), 7.33 (d, ^3^*J*_H,H_ = 8.0 Hz, 2H), 4.17 – 4.10 (m, 1H), 4.08 – 4.00 (m, 2H), 3.81 (q, ^3^*J*_H,H_ = 7.6 Hz, 1H), 3.53 (dd, ^2^*J*_H,H_ = 10.4 Hz, ^3^*J*_H,H_ = 4.5 Hz, 1H), 3.47 (dd, ^2^*J*_H,H_ = 10.4 Hz, ^3^*J*_H,H_ = 4.6 Hz, 1H), 2.44 (s, 3H), 2.30 – 2.20 (m, 1H), 2.02 (quint, ^3^*J*_H,H_ = 8.2 Hz, 1H), 1.91 – 1.84 (m, 1H), 1.79 (q, ^3^*J*_H,H_ = 7.7 Hz, 2H), 1.42 (ddd, ^2^*J*_H,H_ = 12.9 Hz, ^3^*J*_H,H_ = 7.5 Hz, ^3^*J*_H,H_ = 5.2 Hz, 2H), 0.93 (t, ^3^*J*_H,H_ = 7.9 Hz, 9H), 0.86 (s, 9H), 0.83 (s, 9H), 0.55 (q, ^3^*J*_H,H_ = 7.9 Hz, 6H), 0.01 (s, 3H), 0.01 (s, 3H), -0.02 (s, 3H), -0.04 (s, 3H).

A solution of LiAlH_4_ (2.4 M in THF, 1.63 mL, 1.63 mmol, 1.1 equiv.) was added to a solution of tosylate **S14** (2.12 mmol) in 20 mL anhydrous Et_2_O and the mixture was stirred at room temperature for 3 hours. Additional 0.5 equiv. of LiAlH_4_ (2.4 M in THF, 750 µL, 0.75 mmol) were added, the mixture was stirred at room temperature for 90 minutes, then diluted with Et_2_O (40 mL) and filtered on Celite®. The filtrate was washed with water (50 mL), brine (50 mL), dried over MgSO_4_ and concentrated under reduced pressure. The crude was then purified by silica gel column chromatography using pentane/Et_2_O: 98/2, to afford the compound **4** (625 mg, 1.24 mmol, 58% over 2 steps, 74% BRMS) as a colourless oil.

Rf = 0.55 (cyclohexane/EtOAc: 96/4)

IR (neat): ν = 2956, 2857 (CH) cm^-1^.

^1^H NMR (500 MHz, CDCl_3_): δ 4.08 (ddd, ^3^*J*_H,H_ = 7.4 Hz, ^3^*J*_H,H_ = 4.7 Hz, ^4^*J*_H,H_ = 2.8 Hz, 1H, *H_3_*), 3.81 (q, ^3^*J*_H,H_ = 7.4 Hz, 1H, *H_5_*), 3.58 (dd, ^2^*J*_H,H_ = 10.2 Hz, ^3^*J*_H,H_ = 4.7 Hz, 1H, *H_1a_*), 3.48 (dd, ^2^*J*_H,H_ = 10.2 Hz, ^3^*J*_H,H_ = 5.5 Hz, 1H, *H_1b_*), 2.36 – 2.26 (m, 1H, *H_4a_*), 2.07 – 1.94 (m, 2H, *H_2_*, *H_6_*), 1.52 – 1.39 (m, 2H, *H_4b_*, *H_7a_*), 1.35 – 1.23 (m, 1H, *H_7b_*), 0.96 (t, ^3^*J*_H,H_ = 8.0 Hz, 12H, *H_8_*, C*H_3_* TES), 0.88 (s, 9H, C*H_3_* *t*-Bu TBS), 0.87 (s, 9H, C*H_3_* *t*-Bu TBS), 0.58 (q, ^3^*J*_H,H_ = 8.0 Hz, 6H, C*H_2_* TES), 0.03 (s, 3H, C*H_3_* Me-TBS), 0.02 (s, 3H, C*H_3_* Me-TBS), 0.02 (s, 3H, C*H_3_* Me-TBS), 0.02 (s, 3H, C*H_3_* Me-TBS).

^13^C NMR (126 MHz, CDCl_3_): δ 76.9 (*C_5_*), 73.6 (*C_3_*), 61.1 (*C_1_*), 50.6 (*C_2_*), 49.6 (*C_6_*), 45.2 (*C_7_*), 26.0 (6C, *C*H_3_ t-Bu TBS), 20.7 (*C_7_*), 18.2 (*C_q_* TBS), 18.2 (*C_q_* TBS), 13.3 (*C_8_*), 7.0 (3C, *C*H_2_ TES), 4.5 (3C, *C*H_3_ TES), -4.2 (*C*H_3_ Me-TBS), -4.4 (*C*H_3_ Me-TBS),
-4.5 (*C*H_3_ Me-TBS), -4.6 (*C*H_3_ Me-TBS).

[α]_D_^20^ = − 18.3 (8 mg/mL, EtOH).

### Preparation of ethyl (*E*)-11-((1*S*,2*R*,3*R*,5*S*)-3,5-bis((tert-butyldimethylsilyl)oxy)-2-ethylcyclopentyl)-9-oxoundec-10-enoate (**25**)

Oxalyl chloride (416 µL, 4.92 mmol, 4 equiv.) was added at –70 °C to a solution of DMSO (698 µL, 9.84 mmol, 8 equiv.) in 6 mL anhydrous CH_2_Cl_2_ and the mixture was stirred at –70 °C for 15 minutes prior to the addition of **4** (620 mg, 1.23 mmol, 1 equiv., solution in 6 mL anhydrous CH_2_Cl_2_) and distilled Et_3_N (2.6 mL, 18.5 mmol, 15 equiv.). The mixture was stirred at –70 °C for 15 minutes followed by 3 hours at room temperature and was then treated by the addition of water (30 mL) and extracted with CH_2_Cl_2_ (2 x 20 mL). The combined organic layers were washed with brine (2 x 20 mL), dried over MgSO_4_ and concentrated under reduced pressure. The crude was then purified by a rapid silica gel column chromatography using pentane/Et_2_O: 95/5, to afford the aldehyde **S15** (286 mg, containing approximately 13% of starting material, estimated by ^1^H NMR). Note that 184 mg (0.37 mmol, 30%) of starting material **4** were also isolated.

Rf = 0.3 (cyclohexane/EtOAc: 96/4).

^1^H NMR (400 MHz, CDCl_3_): δ 9.86 (d, ^3^*J*_H,H_ = 2.3 Hz, 1H), 4.49 (dt, ^3^*J*_H,H_ = 7.4 Hz, ^3^*J*_H,H_ = 5.3 Hz, 1H), 3.79 (q, ^3^*J*_H,H_ = 6.7 Hz, 1H), 3.05 (ddd, ^3^*J*_H,H_ = 8.7 Hz, ^3^*J*_H,H_ = 4.5 Hz, ^4^*J*_H,H_ = 2.2 Hz, 1H), 2.37 – 2.21 (m, 2H), 1.61 – 1.52 (m, 1H), 1.48 – 1.38 (m, 1H), 1.34 – 1.23 (m, 1H), 0.98 (t, ^3^*J*_H,H_ = 7.4 Hz, 3H), 0.88 (s, 9H), 0.86 (s, 9H), 0.03 (s, 9H), 0.02 (s, 3H).

A solution of NaHMDS (2M in THF, 650 µL, 1.3 mmol, 1.8 equiv.) was slowly added at 0 °C to a solution of phosphonate **24** (444 mg, 1.38 mmol, 1.9 equiv.) in 30 mL anhydrous THF. The mixture was stirred at 0 °C for 30 minutes and a 0 °C cooled solution of aldehyde **S15** (280 mg, 0.72 mmol, 1 equiv.) in 7 mL anhydrous THF was added. The mixture was then stirred at 0°C for 30 minutes, and for 18 hours at room temperature. The reaction was then quenched by the addition of saturated aqueous NH_4_Cl (20 mL). The mixture was extracted with Et_2_O (3 x 20 mL), the combined organic layers were washed with brine, dried over MgSO_4_ and concentrated under reduced pressure. The crude was then purified by silica gel column chromatography using a gradient pentane/Et_2_O from 95/5 to 90/10, to afford enone **25** (296 mg, 0.51 mmol, 43% over 2 steps, 60% BRMS) as a pale-yellow oil.

Rf = 0.2 (cyclohexane/EtOAc: 94/6)

IR (neat): ν = 2929, 2857 (CH), 1737 (C=O), 1673 (C=O), 1629 (C=C) cm^-1^.

^1^H NMR (500 MHz, CDCl_3_): δ 6.63 (dd, ^3^*J*_H,H_ = 15.6 Hz, ^3^*J*_H,H_ = 10.2 Hz, 1H, *H_11_*), 6.15 (dd, ^3^*J*_H,H_ = 15.7 Hz, ^4^*J*_H,H_ = 0.8 Hz, 1H, *H_10_*), 4.12 (q, ^3^*J*_H,H_ = 7.1 Hz, 2H, *H_2’_*), 3.95 (dt, ^3^*J*_H,H_ = 7.0 Hz, ^3^*J*_H,H_ = 5.0 Hz, 1H, *H_13_*), 3.86 (q, ^3^*J*_H,H_ = 6.4 Hz, 1H, *H_15_*), 2.81 – 2.75 (m, 1H, *H_12_*), 2.50 (t, ^3^*J*_H,H_ = 7.5 Hz, 2H, *H_8_*), 2.36 (dt, ^2^*J*_H,H_ = 14.0 Hz, ^3^*J*_H,H_ = 7.1 Hz, 1H, *H_14a_*), 2.28 (t, ^3^*J*_H,H_ = 7.5 Hz, 2H, *H_2_*), 2.03 (quint, ^3^*J*_H,H_ = 7.7 Hz, 1H, *H_16_*), 1.65 – 1.58 (m, 5H, *H_3_*, *H_7_*, *H_14b_*), 1.37 – 1.28 (m, 7H, *H_4_*, *H_5_*, *H_6_*, *H_17a_*), 1.25 (t, ^3^*J*_H,H_ = 7.1 Hz, 3H, *H_3’_*), 1.26 – 1.17 (m, 1H, *H_17b_*), 0.86 – 0.90 (m, 12H, *H_18_*, C*H_3_* *t*-Bu TBS), , 0.86 (s, 9H, C*H_3_* *t*-Bu TBS), 0.04 (s, 6H, C*H_3_* Me-TBS), 0.00 (s, 3H, C*H_3_* Me-TBS), -0.01 (s, 3H, C*H_3_* Me-TBS).

^13^C NMR (126 MHz, CDCl_3_): δ 200.4 (*C_9_*), 174.0 (*C_1_*), 145.8 (*C_11_*), 131.3 (*C_10_*), 76.0 (*C_15_*), 75.5 (*C_13_*), 60.3 (*C_2’_*), 53.0 (*C_12_*), 52.1 (*C_16_*), 44.7 (*C_14_*), 41.0 (*C_8_*), 34.5 (*C_2_*), 29.2 (2C, *C_4_* or *C_5_* or *C_6_*), 29.1 (*C_4_* or *C_5_* or *C_6_*), 26.0 (3C, *C*H_3_ t-Bu TBS), 25.9 (3C, *C*H_3_ t-Bu TBS), 25.0 (*C_7_*), 24.3 (*C_3_*), 21.8 (*C_17_*), 18.1 (2C, *C_q_* TBS), 14.4 (*C_3’_*), 12.8 (*C_18_*), -4.2 (*C*H_3_ Me-TBS), -4.5 (*C*H_3_ Me-TBS), -4.5 (*C*H_3_ Me-TBS), -4.6 (*C*H_3_ Me-TBS).

HRMS (TOF ESI+) m/z: calcd for C_32_H_63_O_5_Si_2_ : 583.4209 [M+H]^+^, found: 583.4213 [M+H]^+^

[α]_D_^20^ = − 2.6 (8 mg/mL EtOH).

### Preparation of 3,4-dihydroxyphenethyl (*S*,*E*)-11-((1*S*,2*R*,3*R*,5*S*)-2-ethyl-3,5-dihydroxy cyclopentyl)-9-hydroxyundec-10-enoate – 9-F_1t_-PhytoP-HT (**2**)

A solution of LiOH.H_2_O (129 mg, 3.1 mmol, 6 equiv.) in distilled water (10 mL) was added to a solution of **25** (290 mg, 0.51 mmol, 1 equiv.) in THF (10 mL). The mixture was stirred at 35 °C for 24 hours and then acidified by the addition of aqueous HCl (0.5 M, 20 mL). The crude was extracted by EtOAc (3 x 20 mL), the combined organic layers were washed with HCl 0.5 M (20 mL) and brine (20 mL), dried over MgSO_4_ and the solvents were removed under reduced pressure. The crude acid was obtained as a colourless oil and directly engaged in the next step.

DCC (111 mg, 0.54 mmol, 1.2 equiv.), DMAP (33 mg, 0.27 mmol, 0.6 equiv.) and **22** (209 mg, 0.54 mmol, 1 equiv., solution in 2 mL anhydrous CH_2_Cl_2_) were successively added to a solution of the previously prepared acid (248 mg, 0.45 mmol, 1 equiv.) in 8 mL anhydrous CH_2_Cl_2_. The mixture was stirred at room temperature for 16 hours and was then filtered on Celite®. The filtrate was concentrated under reduced pressure and the crude was purified by silica gel column chromatography using pentane/Et_2_O: 95/5, to afford the pure ester **S16** (305 mg, 0.33 mmol, 65% over 2 steps) as a colourless oil.

Rf = 0.75 (cyclohexane/EtOAc: 8/2).

IR (neat): ν = 2929, 2857 (CH), 1738 (C=O), 1677 (C=O), 1625 (C=C), 1511 (C=C Ar) cm^-1^.

^1^H NMR (400 MHz, CDCl_3_): δ 6.74 (d, ^3^*J*_H,H_ = 8.1 Hz, 1H, *H_Ar_*), 6.69 – 6.59 (m, 3H, *H_Ar_*, *H_11_*), 6.15 (d, ^3^*J*_H,H_ = 15.7 Hz, 1H, *H_10_*), 4.21 (t, ^3^*J*_H,H_ = 7.2 Hz, 2H, *H_2’_*), 3.98 – 3.92 (m, 1H, *H_13_*), 3.86 (q, ^3^*J*_H,H_ = 6.4 Hz, 1H, *H_15_*), 2.84 – 2.73 (m, 3H, *H_3’_*, *H_12_*), 2.50 (t, ^3^*J*_H,H_ = 7.4 Hz, 2H, *H_8_*), 2.36 (dt, ^3^*J*_H,H_ = 14.0 Hz, ^3^*J*_H,H_ = 7.1 Hz, 1H, *H_14a_*), 2.27 (t, ^3^*J*_H,H_ = 7.6 Hz, 2H, *H_2_*), 2.03 (quint, ^3^*J*_H,H_ = 7.5 Hz, 1H, *H_16_*), 1.65 – 1.55 (m, 5H, *H_3_*, *H_7_*, *H_14b_*), 1.38 – 1.17 (m, 8H, *H_4_*, *H_5_*, *H_6_*, *H_17_*), 0.98 (s, 9H, C*H_3_* *t*-Bu TBS), 0.98 (s, 9H, C*H_3_* *t*-Bu TBS), 0.90 – 0.86 (m, 12H, *H_18_*, C*H_3_* *t*-Bu TBS), 0.86 (s, 9H, C*H_3_* *t*-Bu TBS), 0.18 (s, 6H, C*H_3_* Me-TBS), 0.18 (s, 6H, C*H_3_* Me-TBS), 0.04 (s, 6H, C*H_3_* Me-TBS), 0.00 (s, 3H, C*H_3_* Me-TBS), -0.01 (s, 3H, C*H_3_* Me-TBS).

^13^C NMR (126 MHz, CDCl_3_): δ 200.4 (*C_9_*), 173.9 (*C_1_*), 146.8 (*C_Ar_*), 145.7 (*C_11_*), 145.6 (*C_Ar_*), 131.3 (*C_10_*), 131.0 (*C_Ar_*), 121.9 (*C*H_Ar_), 121.9 (*C*H_Ar_), 121.1 (*C*H_Ar_), 76.0 (*C_15_*), 75.5 (*C_13_*), 65.1 (*C_2’_*), 53.0 (*C_12_*), 52.1 (*C_16_*), 44.7 (*C_14_*), 41.0 (*C_8_*), 34.6 (*C_3’_*), 34.4 (*C_2_*), 29.3 (2C, *C_4_* or *C_5_* or *C_6_*), 29.1 (*C_4_* or *C_5_* or *C_6_*), 26.1 (6C, *C*H_3_ t-Bu TBS), 26.0 (3C, *C*H_3_ t-Bu TBS), 25.9 (3C, *C*H_3_ t-Bu TBS), 25.0 (*C_7_*), 24.3 (*C_3_*), 21.8 (*C_17_*), 18.6 (2C, *C_q_* TBS), 18.1(2C, *C_q_* TBS), 12.8 (*C_18_*), -4.0 (4C, *C*H_3_ Me-TBS), -4.2 (*C*H_3_ Me-TBS), -4.4 (*C*H_3_ Me-TBS), -4.5 (*C*H_3_ Me-TBS), -4.6 (*C*H_3_ Me-TBS).

HRMS (TOF ESI+) m/z: calcd for C_27_H_54_O_5_Si_2_ : 919.6149 [M+H]^+^, found: 919.6153 [M+H]^+^.

[α]_D_^20^ = − 1.6 (8 mg/mL EtOH).

BH_3_.Me_2_S (2M in THF, 130 µL, 0.26 mmol, 2 equiv.) was added at 0 °C to a solution of (*R*)-2-methyl-CBS-oxazaborolidine (1M in toluene, 290 µL, 0.29 mmol, 2.2 equiv.) in 3 mL anhydrous THF. The mixture was stirred at 0°C for 15 minutes and a 0 °C cooled solution of **S16** (120 mg, 0.13 mmol, 1 equiv., solution in 3 mL anhydrous THF) was added. The mixture was stirred at 0 °C for 30 minutes, then quenched by the addition of MeOH (5 mL) and the solvents were removed under reduced pressure. The crude was then purified by silica gel column chromatography using pentane/Et_2_O gradient from 95/5 to 92/8, to afford compound **S17** (116 mg, small traces of CBS copula) as a colourless oil.

Rf = 0.5 (cyclohexane/EtOAc: 9/1)

IR (neat): ν = 3430 (OH), 2929, 2857 (CH), 1738 (C=O), 1511 (C=C Ar) cm^-1^.

^1^H NMR (500 MHz, CDCl_3_): δ 6.74 (d, ^3^*J*_H,H_ = 8.1 Hz, 1H, *H_Ar_*), 6.68 (d, ^4^*J*_H,H_ = 2.1 Hz, 1H, *H_Ar_*), 6.64 (dd, ^3^*J*_H,H_ = 8.1 Hz, ^4^*J*_H,H_ = 2.2 Hz, 1H, *H_Ar_*), 5.51 (dd, ^3^*J*_H,H_ = 15.3 Hz, ^3^*J*_H,H_ = 6.6 Hz, 1H, *H_10_*), 5.41 (dd, ^3^*J*_H,H_ = 15.5, ^3^*J*_H,H_ = 9.6 Hz, 1H, *H_11_*), 4.21 (t, ^3^*J*_H,H_ = 7.2 Hz, 2H, *H_2’_*), 4.10 – 4.02 (m, 1H, *H_9_*), 3.86 (dt, ^3^*J*_H,H_ = 6.9, ^3^*J*_H,H_ = 4.6 Hz, 1H, *H_13_*), 3.78 (q, ^3^*J*_H,H_ = 7.0 Hz, 1H, *H_15_*), 2.80 (t, ^3^*J*_H,H_ = 7.2 Hz, 2H, *H_3’_*), 2.63 – 2.57 (m, 2H, *H_12_*), 2.35 – 2.29 (m, 1H, *H_14a_*), 2.27 (t, ^3^*J*_H,H_ = 7.6 Hz, 2H, *H_2_*), 1.95 (quint, ^3^*J*_H,H_ = 6.9 Hz, 1H, *H_16_*), 1.65 – 1.43 (m, 5H, *H_3_*, *H_8_*, *H_14b_*), 1.39 – 1.17 (m, 10H, *H_4_*, *H_5_*, *H_6_*, *H_7_*, *H_17_*), 0.98 (s, 9H, C*H_3_* *t*-Bu TBS), 0.98 (s, 9H, C*H_3_* *t*-Bu TBS), 0.92 – 0.85 (m, 21H, *H_18_*, C*H_3_* *t*-Bu TBS), 0.18 (s, 6H, C*H_3_* Me-TBS), 0.18 (s, 6H, C*H_3_* Me-TBS), 0.03 (s, 3H, C*H_3_* Me-TBS), 0.03 (s, 3H, C*H_3_* Me-TBS), 0.01 (s, 3H, C*H_3_* Me-TBS), 0.01 (s, 3H, C*H_3_* Me-TBS).

^13^C NMR (101 MHz, CDCl_3_): δ 173.9 (*C_1_*), 146.8 (*C_Ar_*), 145.6 (*C_Ar_*), 135.1 (*C_10_*), 131.0 (*C_Ar_*), 130.1 (*C_11_*), 121.9 (*C*H_Ar_), 121.9 (*C*H_Ar_), 121.1 (*C*H_Ar_), 76.2 (*C_13_*, *C_15_*), 73.1 (*C_9_*), 65.1 (*C_2’_*), 52.6 (*C_12_*), 51.3 (*C_16_*), 44.7 (*C_14_*), 37.5 (*C_8_*), 34.6 (*C_3’_*), 34.4 (*C_2_*), 29.5 (*C_4_* or *C_5_* or *C_6_*), 29.4 (*C_4_* or *C_5_* or *C_6_*), 29.3 (*C_4_* or *C_5_* or *C_6_*), 26.1 (6C, *C*H_3_ t-Bu TBS), 26.0 (6C, *C*H_3_ t-Bu TBS), 25.5 (*C_7_*), 25.0 (*C_3_*), 21.6 (*C_18_*), 18.6 (2C, *C_q_* TBS), 18.2 (*C_q_* TBS), 18.2 (*C_q_* TBS), 12.8 (*C_18_*), -4.0 (4C, *C*H_3_ Me-TBS), -4.2 (*C*H_3_ Me-TBS), -4.4 (2C, *C*H_3_ Me-TBS), -4.6 (*C*H_3_ Me-TBS).

HRMS (TOF ESI+) m/z: calcd for C_50_H_100_NO_7_Si_4_ : 938.6571 [M+NH_4_]^+^, found: 938.6557 [M+NH_4_]^+^.

In a Teflon® flask, Et_3_N.3HF (101 µL, 0.62 mmol, 6 equiv.) was added to a solution of **S17** (95 mg, 0.103 mmol, 1 equiv.) in 2 mL anhydrous THF and the mixture was stirred at room temperature for 12 hours. At this point, 6 more equivalents of Et_3_N.3HF (101 µL) were added and the mixture was stirred at room temperature for another 12 hours. The mixture was then treated by a dropwise addition of a saturated aqueous solution of NaHCO_3_ (until gas formation is over) and 5 mL brine were added to facilitate the extraction. The crude was extracted by EtOAc (3 x 10 mL), the combined organic layers were washed with brine, dried over MgSO_4_ and concentrated under reduced pressure. The crude was then purified by silica gel column chromatography using a gradient EtOAc/MeOH from100/0 to 98/2, to afford 9-F_1t_-PhytoP-HT **2** (26 mg, 0.06 mmol, 53% over 2 steps) as a colourless oil.

Rf = 0.2 (EtOAc/MeOH: 98/2)

IR (neat): ν = 3441 (OH), 2930, 2858 (CH), 1712 (C=O), 1520 (C=C Ar) cm^-1^.

^1^H NMR (500 MHz, MeOD): δ 6.68 (d, ^3^*J*_H,H_ = 8.0 Hz, 1H, *H_Ar_*), 6.65 (d, ^3^*J*_H,H_ = 2.1 Hz, 1H, *H_Ar_*), 6.53 (dd, ^3^*J*_H,H_ = 8.0 Hz, ^4^*J*_H,H_ = 2.1 Hz, 1H, *H_Ar_*), 5.53 (dd, ^3^*J*_H,H_ = 15.4 Hz, ^3^*J*_H,H_ = 6.9 Hz, 1H, *H_10_*), 5.45 (dd, ^3^*J*_H,H_ = 15.4 Hz, ^3^*J*_H,H_ = 9.4 Hz, 1H, *H_11_*), 4.20 (t, *^3^J_H,H_* = 7.0 Hz, 2H, *H_2’_*), 4.00 (q, ^3^*J*_H,H_ = 6.3 Hz, 1H, *H_9_*), 3.90 (dt, ^3^*J*_H,H_ = 6.8 Hz, ^3^*J*_H,H_ = 4.4 Hz, 1H, *H_13_*), 3.86 – 3.81 (m, 1H, *H_15_*), 2.76 (t, ^3^*J*_H,H_ = 7.0 Hz, 2H, *H_3’_*), 2.66 (td, ^3^*J*_H,H_ = 8.2 Hz, ^3^*J*_H,H_ = 4.0 Hz, 1H, *H_12_*), 2.49 – 2.42 (m, 1H, *H_14a_*), 2.28 (t, ^3^*J*_H,H_ = 7.4 Hz, 2H, *H_2_*), 2.05 – 1.98 (apparent quint, ^3^*J*_H,H_ = 7.3 Hz, 1H, *H_16_*), 1.60 – 1.43 (m, 5H, *H_3_*, *H_8_*, *H_14b_*), 1.42 – 1.22 (m, 10H, *H_4_*, *H_5_*, *H_6_*, *H_7_*, *H_17_*), 0.93 (t, ^3^*J*_H,H_ = 7.4 Hz, 3H, *H_18_*).

^13^C NMR (126 MHz, MeOD): δ 175.6 (*C_1_*), 146.3 (*C_Ar_*), 144.9 (*C_Ar_*), 136.5 (*C_10_*), 130.7 (*C_Ar_*), 129.8 (*C_11_*), 121.2 (*C*H_Ar_), 117.0 (*C*H_Ar_), 116.3 (*C*H_Ar_), 76.6 (*C_13_* or *C_15_*), 76.5 (*C_13_* or *C_15_*), 73.4 (*C_9_*), 66.4 (*C_2’_*), 53.7 (*C_12_*), 52.4 (*C_16_*), 43.8 (*C_14_*), 38.5 (*C_8_*), 35.5 (*C_3’_*), 35.1 (*C_2_*), 30.5 (*C_4_* or *C_5_* or *C_6_* or *C_7_*), 30.3 (*C_4_* or *C_5_* or *C_6_* or *C_7_*), 30.1 (*C_4_* or *C_5_* or *C_6_* or *C_7_*), 26.5 (*C_4_* or *C_5_* or *C_6_* or *C_7_*), 26.0 (*C_3_*), 22.6 (*C_17_*), 13.0 (*C_18_*).

HRMS (TOF ESI+) m/z: calcd for C_26_H_39_O_7_ : 463.2701 [M-H]^-^, found: 463.2699 [M-H]^-^.

[α]_D_^20^ = − 6.7 (1 mg/mL EtOH).

The diastereomeric ratio (determinated by HPLC) is 98:2 in favor of the (*S*)-epimer at the C9 center.

### Preparation of 3,4-dihydroxyphenethyl (*R*,*E*)-11-((1*S*,2*R*,3*R*,5*S*)-2-ethyl-3,5-dihydroxycyclopentyl)-9-hydroxyundec-10-enoate (**9*-epi-*2**)

The same procedure previously described to access **S17** from **S16** was applied from **9-*epi***-**S16** (120 mg, 0.13 mmol, 1 equiv.) using (*S*)-2-methyl-CBS-oxazaborolidine (1M in toluene, 290 µL, 0.29 mmol, 2.2 equiv.) and BH_3_.Me_2_S (2M in THF, 130 µL, 0.26 mmol, 2 equiv.) to afford **9*-epi-*S17** as a colourless oil (105 mg, small traces of CBS copula).

Rf = 0.5 (cyclohexane/EtOAc: 9/1).

IR (neat): ν = 3430 (OH), 2929, 2857 (CH), 1738 (C=O), 1511 (C=C Ar) cm^-1^.

^1^H NMR (500 MHz, CDCl_3_): δ 6.74 (d, ^3^*J*_H,H_ = 8.1 Hz, 1H, *H_Ar_*), 6.68 (d, ^4^*J*_H,H_ = 2.1 Hz, 1H, *H_Ar_*), 6.64 (dd, ^3^*J*_H,H_ = 8.1 Hz, ^4^*J*_H,H_ = 2.2 Hz, 1H, *H_Ar_*), 5.50 (dd, ^3^*J*_H,H_ = 15.3 Hz, ^3^*J*_H,H_ = 6.8 Hz, 1H, *H_10_*), 5.41 (dd, ^3^*J*_H,H_ = 15.5, ^3^*J*_H,H_ = 9.8 Hz, 1H, *H_11_*), 4.21 (t, ^3^*J*_H,H_ = 7.2 Hz, 2H, *H_2’_*), 4.08 – 4.02 (m, 1H, *H_9_*), 3.87 (dt, ^3^*J*_H,H_ = 6.9, ^3^*J*_H,H_ = 4.4 Hz, 1H, *H_13_*), 3.78 (q, ^3^*J*_H,H_ = 7.2 Hz, 1H, *H_15_*), 2.80 (t, ^3^*J*_H,H_ = 7.1 Hz, 2H, *H_3’_*), 2.63 – 2.56 (m, 2H, *H_12_*), 2.34 – 2.29 (m, 1H, *H_14a_*), 2.27 (t, ^3^*J*_H,H_ = 7.5 Hz, 2H, *H_2_*), 2.00 – 1.92 (m, 1H, *H_16_*), 1.64 – 1.42 (m, 5H, *H_3_*, *H_8_*, *H_14b_*), 1.40 – 1.27 (m, 9H, *H_4_*, *H_5_*, *H_6_*, *H_7_*, *H_17a_*), 1.20 – 1.13 (m, 1H, *H_17b_*), 0.98 (s, 9H, C*H_3_* *t*-Bu TBS), 0.98 (s, 9H, C*H_3_* *t*-Bu TBS), 0.89 – 0.85 (m, 21H, *H_18_*, C*H_3_* *t*-Bu TBS), 0.18 (s, 6H, C*H_3_* Me-TBS), 0.18 (s, 6H, C*H_3_* Me-TBS), 0.03 (s, 3H, C*H_3_* Me-TBS), 0.03 (s, 3H, C*H_3_* Me-TBS), 0.02 (s, 6H, C*H_3_* Me-TBS).

^13^C NMR (126 MHz, CDCl_3_): δ 173.9 (*C_1_*), 146.8 (*C_Ar_*), 145.6 (*C_Ar_*), 135.2 (*C_10_*), 131.0 (*C_Ar_*), 130.2 (*C_11_*), 121.9 (*C*H_Ar_), 121.9 (*C*H_Ar_), 121.1 (*C*H_Ar_), 76.3 (*C_13_* or *C_15_*), 76.3 (*C_13_* or *C_15_*), 73.2 (*C_9_*), 65.1 (*C_2’_*), 52.8 (*C_12_*), 51.2 (*C_16_*), 44.6 (*C_14_*), 37.5 (*C_8_*), 34.6 (*C_3’_*), 34.5 (*C_2_*), 29.5 (*C_4_* or *C_5_* or *C_6_*), 29.4 (*C_4_* or *C_5_* or *C_6_*), 29.2 (*C_4_* or *C_5_* or *C_6_*), 26.1 (6C, *C*H_3_ t-Bu TBS), 26.0 (6C, *C*H_3_ t-Bu TBS), 25.6 (*C_7_*), 25.0 (*C_3_*), 21.7 (*C_18_*), 18.6 (2C, *C_q_* TBS), 18.2 (*C_q_* TBS), 18.2 (*C_q_* TBS), 12.7 (*C_18_*), -4.0 (4C, *C*H_3_ Me-TBS), -4.2 (*C*H_3_ Me-TBS), -4.4 (*C*H_3_ Me-TBS), -4.4 (*C*H_3_ Me-TBS), -4.6 (*C*H_3_ Me-TBS).

HRMS (TOF ESI+) m/z: calcd for C_50_H_100_NO_7_Si_4_ : 938.6571 [M+NH_4_]^+^, found: 938.6579 [M+NH_4_]^+^.

The same procedure previously described to access **2** from **S18** was applied from **9*-epi-*S18** (85 mg, 0.092 mmol, 1 equiv.) using Et_3_N.3HF (90 µL, 0.55 mmol, 6 equiv.) to afford pure 9-*epi*-9-F_1t_-PhytoP-HT **9*-epi-*2** as a colourless oil (16 mg, 0.03 mmol, 33% over 2 steps). Note that a mixture of **2** and **9*-epi-*2** was also isolated (10 mg, 0.02 mmol, 20% over 2 steps).

Rf = 0.2 (EtOAc/MeOH: 98/2).

IR (neat): ν = 3441 (OH), 2926, 2854 (CH), 1712 (C=O), 1520 (C=C Ar) cm^-1^.

^1^H NMR (500 MHz, MeOD): δ 6.68 (d, ^3^*J*_H,H_ = 8.0 Hz, 1H, *H_Ar_*), 6.65 (d, ^3^*J*_H,H_ = 2.1 Hz, 1H, *H_Ar_*), 6.53 (dd, ^3^*J*_H,H_ = 8.0 Hz, ^4^*J*_H,H_ = 2.1 Hz, 1H, *H_Ar_*), 5.49 (dd, ^3^*J*_H,H_ = 15.4 Hz, ^3^*J*_H,H_ = 6.9 Hz, 1H, *H_10_*), 5.41 (dd, ^3^*J*_H,H_ = 15.4 Hz, ^3^*J*_H,H_ = 9.5 Hz, 1H, *H_11_*), 4.20 (t, *^3^J_H,H_* = 7.0 Hz, 2H, *H_2’_*), 3.98 (q, ^3^*J*_H,H_ = 6.7 Hz, 1H, *H_9_*), 3.91 (dt, ^3^*J*_H,H_ = 6.7 Hz, ^3^*J*_H,H_ = 4.3 Hz, 1H, *H_13_*), 3.86 – 3.81 (m, 1H, *H_15_*), 2.76 (t, ^3^*J*_H,H_ = 7.0 Hz, 2H, *H_3’_*), 2.66 (td, ^3^*J*_H,H_ = 8.8 Hz, ^3^*J*_H,H_ = 3.8 Hz, 1H, *H_12_*), 2.46 (ddd, ^2^*J*_H,H_ = 14.4 Hz, ^3^*J*_H,H_ = 7.7 Hz, ^3^*J*_H,H_ = 6.9 Hz, 1H, *H_14a_*), 2.28 (t, ^3^*J*_H,H_ = 7.4 Hz, 2H, *H_2_*), 2.05 – 1.98 (apparent quint, ^3^*J*_H,H_ = 7.2 Hz, 1H, *H_16_*), 1.60 – 1.50 (m, 4H, *H_3_*, *H_8a_*, *H_14b_*), 1.48 – 1.39 (m, 2H, *H_8b_*, *H_17a_*), 1.36 – 1.24 (m, 9H, *H_4_*, *H_5_*, *H_6_*, *H_7_*, *H_17b_*), 0.93 (t, ^3^*J*_H,H_ = 7.4 Hz, 3H, *H_18_*).

^13^C NMR (126 MHz, MeOD): δ 175.6 (*C_1_*), 146.3 (*C_Ar_*), 144.9 (*C_Ar_*), 136.6 (*C_10_*), 130.7 (*C_Ar_*), 130.6 (*C_11_*), 121.2 (*C*H_Ar_), 117.0 (*C*H_Ar_), 116.3 (*C*H_Ar_), 76.6 (*C_13_* or *C_15_*), 76.5 (*C_13_* or *C_15_*), 73.8 (*C_9_*), 66.4 (*C_2’_*), 54.0 (*C_12_*), 52.4 (*C_16_*), 43.8 (*C_14_*), 38.4 (*C_8_*), 35.5 (*C_3’_*), 35.1 (*C_2_*), 30.4 (*C_4_* or *C_5_* or *C_6_* or *C_7_*), 30.4 (*C_4_* or *C_5_* or *C_6_* or *C_7_*), 30.0 (*C_4_* or *C_5_* or *C_6_* or *C_7_*), 26.5 (*C_4_* or *C_5_* or *C_6_* or *C_7_*), 26.0 (*C_3_*), 22.7 (*C_17_*), 13.0 (*C_18_*).

HRMS (TOF ESI+) m/z: calcd for C_26_H_39_O_7_ : 463.2701 [M-H]^-^, found: 463.2699 [M-H]^-^.

[α]_D_^20^ = − 12.0 (1 mg/mL EtOH).

The diastereomeric ratio (determinated by HPLC) is 95:5 in favor of the (*R*)-epimer at the C9 center.

## Towards the synthesis of the 9-F_1t_-PhytoP and its C9-epimer

### Preparation of (*S*,*E*)-11-((1*S*,2*R*,3*R*,5*S*)-2-ethyl-3,5-dihydroxycyclopentyl)-9-hydroxyundec-10-enoic acid – 9-F_1t_-PhytoP (**S19**)

BH_3_.Me_2_S (2M in THF, 322 µL, 0.64 mmol, 1.25 equiv.) was added at 0 °C to a solution of (*R*)-2-methyl-CBS-oxazaborolidine (1M in toluene, 760 µL, 0.76 mmol, 1.5 equiv.) in anhydrous THF (7 mL). The mixture was stirred at 0 °C for 5 minutes and a cold solution of **25** (300 mg, 0.51 mmol, 1 equiv., solution in 7 mL anhydrous THF) was added. The mixture was stirred at 0 °C for 30 minutes, then quenched by the addition of MeOH (1 mL) and the solvents were removed under reduced pressure. The crude was then purified by silica gel column chromatography using pentane/Et_2_O 90/10 as eluent.

To a solution of the previously prepared compound (130 mg, 0.22 mmol, 1 equiv.) in THF (20 mL) and MeOH (10 mL) at 0 °C was added HCl 0.5N/MeOH (4.5 mL, 2.22 mmol, 10 equiv.) and the reaction was stirred at room temperature for 2.5 hours. The reaction was quenched by the addition of NaHCO_3_ saturated solution. The aqueous phase was extracted with EtOAc (3 x 20 mL), washed with brine, dried over MgSO_4_ and concentrated under vacuum. The crude product was purified by silica gel column chromatography using 100% EtOAc as eluent.

To a solution of the previously prepared compound (90 mg, 0.25 mmol, 1 equiv.) in THF (2.5 mL) and water (2.5 mL) was added LiOH.H_2_O (64 mg, 1.51 mmol, 6 equiv.) and the reaction was stirred at room temperature for 3 hours. The reaction was quenched by slow addition of an aqueous solution of NaHSO_4_ (1M), until acidic pH, and the aqueous phase was extracted with EtOAc (3 x 2 mL), washed with brine, dried over MgSO_4_ and concentrated under vacuum. The crude product was purified by silica gel column chromatography using EtOAc/MeOH: 80/20 to afford 9-F_1t_-PhytoP (47 mg, 0.14 mmol, 12% over 3 steps) as a colorless oil.

Rf = 0.23 (EtOAc/HCOOH: 99/1).

^1^H NMR (300 MHz, MeOD): 5.53 (dd, ^3^*J*_H,H_ = 15.4 Hz, ^3^*J*_H,H_ = 5.8 Hz, 1H, *H_10_*), 5.44 (dd, ^3^*J*_H,H_ = 15.4 Hz, ^3^*J*_H,H_ = 8.6 Hz, 1H, *H_11_*), 4.00 (q, ^3^*J*_H,H_ = 6.0 Hz, 1H, *H_9_*), 3.90 (dt, ^3^*J*_H,H_ = 7.2 Hz, ^3^*J*_H,H_ = 4.5 Hz, 1H, *H_13_*), 3.88 – 3.80 (m, 1H, *H_15_*), 2.66 (td, ^3^*J*_H,H_ = 8.1 Hz, ^3^*J*_H,H_ = 4.0 Hz, 1H, *H_12_*), 2.54 – 2.39 (m, 1H, *H_14a_*), 2.18 (t, ^3^*J*_H,H_ = 7.4 Hz, 2H, *H_2_*), 1.94 (apparent quint, ^3^*J*_H,H_ = 7.2 Hz, 1H, *H_16_*), 1.69 – 1.44 (m, 5H, *H_3_*, *H_8_*, *H_14b_*), 1.43 – 1.26 (m, 10H, *H_4_*, *H_5_*, *H_6_*, *H_7_*, *H_17_*), 0.94 (t, ^3^*J*_H,H_ = 7.4 Hz, 3H, *H_18_*).

^13^C NMR (75 MHz, MeOD): 136.5 (*C_10_*), 129.8 (*C_11_*), 76.6 (*C_13_* or *C_15_*), 76.5 (*C_13_* or *C_15_*), 73.4 (*C_9_*), 53.7 (*C_12_*), 52.4 (*C_16_*), 43.8 (*C_14_*), 38.7 (*C_8_*), 38.5 (*C_2_*), 30.8 (*C_4_* or *C_5_* or *C_6_* or *C_7_*), 30.7 (2C, *C_4_* or *C_5_* or *C_6_* or *C_7_*), 27.4 (*C_4_* or *C_5_* or *C_6_* or *C_7_*), 26.6 (*C_3_*), 22.6 (*C_17_*), 13.1 (*C_18_*).

[α]_D_^20^ = + 23.3 (c = 0,12, MeOH)

### Preparation of (*R*,*E*)-11-((1*S*,2*R*,3*R*,5*S*)-2-ethyl-3,5-dihydroxycyclopentyl)-9-hydroxyundec-10-enoic acid – 9-*epi*-9-PhytoP (**9-*epi*-S19**)

BH_3_**^.^**Me_2_S (2M in THF, 322 µL, 0.64 mmol, 1.25 equiv.) was added at 0 °C to a solution of (*R*)-2-methyl-CBS-oxazaborolidine (1M in toluene, 760 µL, 0.76 mmol, 1.5 equiv.) in anhydrous THF (7 mL). The mixture was stirred at
0 °C for 5 minutes and a cold solution of **25** (300 mg, 0.51 mmol, 1 equiv., solution in 7 mL anhydrous THF) was added. The mixture was stirred at 0 °C for 30 minutes, then quenched by the addition of MeOH (1 mL) and the solvents were removed under reduced pressure. The crude was then purified by silica gel column chromatography using pentane/Et_2_O 90/10 as eluent.

To a solution of the previously prepared compound (240 mg, 0.41 mmol, 1 equiv.) in THF (40 mL) and MeOH (20 mL) at 0 °C was added HCl 0.5N/MeOH (8.2 mL, 4.1 mmol, 10 equiv.) and the reaction was stirred at room temperature for 2.5 hours. The reaction was quenched by the addition of NaHCO_3_ saturated solution. The aqueous phase was extracted with EtOAc (3 x 20 mL), washed with brine, dried over MgSO_4_ and concentrated under vacuum. The crude product was purified by silica gel column chromatography using 100% EtOAc as eluent.

To a solution of the previously prepared compound (90 mg, 0.25 mmol, 1 equiv.) in THF (2.5 mL) and water (2.5 mL) was added LiOH.H_2_O (64 mg, 1.51 mmol, 6 equiv.) and the reaction was stirred at room temperature for 3 hours. The reaction was quenched by slow addition of an aqueous solution of NaHSO_4_ (1M), until acidic pH, and the aqueous phase was extracted with EtOAc (3 x 2 mL), washed with brine, dried over MgSO_4_ and concentrated under vacuum. The crude product was purified by silica gel column chromatography using EtOAc/MeOH: 80/20 to afford 9-epi-9-F_1t_-PhytoP **9-*epi*-S19** (48 mg, 0.14 mmol, 30% over 3 steps) as a colorless oil.

Rf = 0.23 (EtOAc/HCOOH: 99/1).

^1^H NMR (300 MHz, MeOD): 5.49 (dd, ^3^*J*_H,H_ = 15.3 Hz, ^3^*J*_H,H_ = 6.5 Hz, 1H, *H_10_*), 5.40 (dd, ^3^*J*_H,H_ = 15.3 Hz, ^3^*J*_H,H_ = 9.0 Hz, 1H, *H_11_*), 3.98 (q, ^3^*J*_H,H_ = 6.3 Hz, 1H, *H_9_*), 3.91 (dt, ^3^*J*_H,H_ = 6.7 Hz, ^3^*J*_H,H_ = 4.3 Hz, 1H, *H_13_*), 3.83 (q, ^3^*J*_H,H_ = 7.5 Hz, 1H, *H_15_*), 2.65 (td, ^3^*J*_H,H_ = 8.3 Hz, ^3^*J*_H,H_ = 3.5 Hz, 1H, *H_12_*), 2.53 – 2.39 (m, 1H, *H_14a_*), 2.32 – 2.15 (m, 2H, *H_2_*), 1.93 (apparent quint, ^3^*J*_H,H_ = 7.4 Hz, 1H, *H_16_*), 1.60 – 1.43 (m, 5H, *H_3_*, *H_8_*, *H_14b_*), 1.47 – 1.23 (m, 10H, *H_4_*, *H_5_*, *H_6_*, *H_7_*, *H_17_*), 0.92 (t, ^3^*J*_H,H_ = 7.4 Hz, 3H, *H_18_*).

^13^C NMR (75 MHz, MeOD): 180.6 (*C_1_*), 136.6 (*C_10_*), 130.6 (*C_11_*), 76.6 (*C_13_* or *C_15_*), 76.4 (*C_13_* or *C_15_*), 73.8 (*C_9_*), 54.0 (*C_12_*), 52.3 (*C_16_*), 43.7 (*C_14_*), 38.7 (2C, *C_8_* and *C_2_*), 30.5 (2C, *C_4_* or *C_5_* or *C_6_* or *C_7_*), 30.4 (*C_4_* or *C_5_* or *C_6_* or *C_7_*), 26.7 (*C_4_* or *C_5_* or *C_6_* or *C_7_*), 26.6 (*C_3_*), 22.7 (*C_17_*), 13.0 (*C_18_*).

[α]_D_^20^ = + 7.6 (c = 0,21, MeOH)

## Determination of the absolute configuration of the stereocenters

### Derivatization as mandelates and NMR study

#### Preparation of ethyl (*S*,*E*)-11-((1*S*,2*R*,3*R*,5*S*)-3,5-bis((tert-butyldimethylsilyl)oxy)-2-ethylcyclopentyl)-9-hydroxyundec-10-enoate (**S20**)

BH_3_**^.^**Me_2_S (2M in THF, 105 µL, 0.21 mmol, 2 equiv.) was added at 0 °C to a solution of (*R*)-2-methyl-CBS-oxazaborolidine (1M in toluene, 226 µL, 0.23 mmol, 2.2 equiv.) in 2.5 mL anhydrous THF. The mixture was stirred at 0 °C for 15 minutes and a 0 °C cooled solution of **25** (60 mg, 0.10 mmol, 1 equiv., solution in 2.5 mL anhydrous THF) was added. The mixture was stirred at 0 °C for 30 minutes, then quenched by the addition of MeOH (3 mL) and the solvents were removed under reduced pressure. The crude was then purified by silica gel column chromatography using pentane/Et_2_O gradient from 95/5 to 92/8, to afford compound **S20** (45 mg, 75% yield) as a pale-yellow oil.

^1^H NMR (500 MHz, CDCl_3_): δ 5.51 (dd, ^3^*J*_H,H_ = 15.3 Hz, ^3^*J*_H,H_ = 6.6 Hz, 1H, *H_10_*), 6.15 (dd, ^3^*J*_H,H_ = 15.6 Hz, ^3^*J*_H,H_ = 9.9 Hz, 1H, *H_11_*), 4.12 (q, ^3^*J*_H,H_ = 7.1 Hz, 2H, *H_2’_*), 4.08 – 4.03 (m, 1H, *H_9_*), 3.85 (dt, ^3^*J*_H,H_ = 7.0 Hz, ^3^*J*_H,H_ = 4.6 Hz, 1H, *H_13_*), 3.78 (q, ^3^*J*_H,H_ = 7.0 Hz, 1H, *H_15_*), 2.64 – 2.55 (m, 1H, *H_12_*), 2.36 – 2.27 (m, 1H, *H_14a_*), 2.28 (t, ^3^*J*_H,H_ = 7.4 Hz, 2H, *H_2_*), 1.95 (quint, ^3^*J*_H,H_ = 7.7 Hz, 1H, *H_16_*), 1.57 – 1.44 (m, 3H, *H_8_*, *H_14b_*), 1.40 – 1.28 (m, 11H, *H_3,_ H_4_*, *H_5_*, *H_6_*, *H_7_*, *H_17a_*), 1.25 (t, ^3^*J*_H,H_ = 7.1 Hz, 3H, *H_3’_*), 1.23 – 1.18 (m, 1H, *H_17b_*), 0.85 – 0.91 (m, 12H, *H_18_*, C*H_3_* *t*-Bu TBS), , 0.86 (s, 9H, C*H_3_* *t*-Bu TBS), 0.03 (s, 3H, C*H_3_* Me-TBS), 0.03 (s, 3H, C*H_3_* Me-TBS), 0.01 (s, 3H, C*H_3_* Me-TBS), 0.00 (s, 3H, C*H_3_* Me-TBS).

^13^C NMR (126 MHz, CDCl_3_): δ 174.1 (*C_1_*), 135.1 (*C_10_*), 130.1 (*C_11_*), 76.2 (2C, *C_9_*, *C_15_*), 76.2 (*C_13_*), 60.3 (*C_2’_*), 52.6 (*C_12_*), 51.2 (*C_16_*), 44.6 (*C_14_*), 37.5 (*C_8_*), 34.5 (*C_2_*), 29.5 (*C_4_* or *C_5_* or *C_6_*), 29.4 (*C_4_* or *C_5_* or *C_6_*), 29.2 (*C_4_* or *C_5_* or *C_6_*), 26.0 (6C, *C*H_3_ t-Bu TBS), 25.5 (*C_3_* or *C_7_*), 25.1 (*C_3_* or *C_7_*), 21.5 (*C_17_*), 18.2 (*C_q_* TBS), 18.2 (*C_q_* TBS), 14.4 (*C_3’_*), 12.7 (*C_18_*), -4.2 (*C*H_3_ Me-TBS), -4.4 (2C, *C*H_3_ Me-TBS), -4.6 (*C*H_3_ Me-TBS).

#### Preparation of ethyl (*S*,*E*)-9-((*S*)-2-acetoxy-2-phenylacetoxy)-11-((1*S*,2*R*,3*R*,5*S*)-3,5-bis((tert-butyldimethylsilyl)oxy)-2-ethylcyclopentyl)undec-10-enoate (**S21**)

DMAP (7.3 mg, 0.06 mmol, 3 equiv), EDCI.HCl (11 mg, 0.06 mmol, 3 equiv.) and *S*-acetylmandelic acid (12 mg, 0.06 mmol, 3 equiv.) were added at room temperature to a solution of **S20** (10 mg, 0.02 mmol, 1 equiv.) in dry DCM (500 µL). The mixture was stirred at room temperature for 48 hours, then quenched by the addition of few drops of HCl 1M. The mixture was diluted with 10 mL Et_2_O and washed with NaHCO_3_ (10 mL) and brine (10 mL). The organic phase was dried over MgSO_4_ and concentrated under reduced pressure. The crude was then purified by silica gel column chromatography (spherical silica 30 µm) using pentane/Et_2_O gradient from 90/10 to 80/20, to afford compound **S21** (1 mg, 8% yield) as a colourless oil.

^1^H NMR (500 MHz, CDCl_3_): δ 7.47 – 7.42 (m, 2H, *H_Ar_*), 7.39 – 7.34 (m, 3H, *H_Ar_*), 5.88 (s, 1H, Ph-C*H*-OAc), 5.29 – 5.09 (m, 3H, *H_11_*, *H_10_*, *H_9_*), 4.12 (q, ^3^*J*_H,H_ = 7.1 Hz, 2H, *H_2’_*), 3.70 – 3.59 (m, 2H, *H_13_*, *H_15_*), 2.45 – 2.38 (m, 1H, *H_12_*), 2.28 (t, ^3^*J*_H,H_ = 7.6 Hz, 2H, *H_2_*), 2.19 (s, 3H, C*H_3_* OAc), 2.23 – 2.15 (m, 1H, *H_14a_*), 1.81 (quint, ^3^*J*_H,H_ = 7.7 Hz, 1H, *H_16_*), 1.65 – 1.52* (m, 4H, *H_3_*, *H_8_*, *determined by correlation with H_2_ and H_9_ respectively on COSY spectrum), 1.47 – 1.45 (m, 1H, *H_14b_*), 1.33 – 1.27 (m, 8H, *H_4_*, *H_5_*, *H_6_*, *H_7_*), 1.25 (t, ^3^*J*_H,H_ = 7.1 Hz, 3H, *H_3’_*), 1.22 – 1.17 (m, 4H, including t, ^3^*J*_H,H_ = 7.1 Hz, 3H, *H_3’_* at 1.25 and *H_17a_*), 0.97 – 0.91 (m, 1H, *H_17b_*), 0.87 (s, 9H, C*H_3_* *t*-Bu TBS), 0.83 (s, 9H, C*H_3_* *t*-Bu TBS), 0.71 (t, ^3^*J*_H,H_ = 7.3 Hz, 1H, *H_18_*), 0.03 (s, 3H, C*H_3_* Me-TBS), 0.02 (s, 3H, C*H_3_* Me-TBS), -0.04 (s, 3H, C*H_3_* Me-TBS), -0.05 (s, 3H, C*H_3_* Me-TBS).

^13^C NMR (126 MHz, CDCl_3_): δ 174.1 (*C_1_*), 170.5 (*C*=O), 168.2 (*C*=O), 134.0 (*C_Ar_*), 133.0 (*C_10_*), 129.3 (*C_11_*), 129.3 (*C_Ar_*), 128.8 (2 *C_Ar_*), 127.8 (2 *C_Ar_*), 76.2 (*C_9_*), 76.1 (*C_13_* or *C_15_*), 76.0 (*C_13_* or *C_15_*), 74.7 (Ph-*C*H-OAc), 60.3 (*C_2’_*), 52.7 (*C_12_*), 51.3 (*C_16_*), 44.6 (*C_14_*), 34.7 (*C_2_* or *C_8_*), 34.5 (*C_2_* or *C_8_*), , 29.3 (2C, *C_4_* or *C_5_* or *C_6_*), 29.2 (*C_4_* or *C_5_* or *C_6_*), 26.0 (6C, *C*H_3_ t-Bu TBS), 25.1 (2C, *C_3_*, *C_7_*), 21.4 (*C_17_*), 20.9 (*C*H*_3_* OAc), 18.1 (2 *C_q_* TBS), 14.4 (*C_3’_*), 12.6 (*C_18_*), -4.2 (*C*H_3_ Me-TBS), -4.5 (*C*H_3_ Me-TBS), -4.5 (*C*H_3_ Me-TBS), -4.6 (*C*H_3_ Me-TBS).

#### Preparation of ethyl (*S*,*E*)-9-((R)-2-acetoxy-2-phenylacetoxy)-11-((1*S*,2*R*,3*R*,5*S*)-3,5-bis((tert-butyldimethylsilyl)oxy)-2-ethylcyclopentyl)undec-10-enoate (**S22**)

DMAP (7.3 mg, 0.06 mmol, 3 equiv), EDCI.HCl (11 mg, 0.06 mmol, 3 equiv.) and *R*-acetylmandelic acid (12 mg, 0.06 mmol, 3 equiv.) were added at room temperature to a solution of **S20** (10 mg, 0.02 mmol, 1 equiv.) in dry DCM (500 µL). The mixture was stirred at room temperature for 48 hours, then quenched by the addition of few drops of HCl 1M. The mixture was diluted with 10 mL Et_2_O and washed with NaHCO_3_ (10 mL) and brine (10 mL). The organic phase was dried over MgSO_4_ and concentrated under reduced pressure. The crude was then purified by silica gel column chromatography (spherical silica 30 µm) using pentane/Et_2_O gradient from 90/10 to 80/20, to afford compound **S22** (2.8 mg, 22% yield) as a colourless oil.

^1^H NMR (500 MHz, CDCl_3_): δ 7.49 – 7.43 (m, 2H, *H_Ar_*), 7.40 – 7.34 (m, 3H, *H_Ar_*), 5.89 (s, 1H, Ph-C*H*-OAc), 5.47 (^3^*J*_H,H_ = 15.6 Hz, ^3^*J*_H,H_ = 9.6 Hz, *H_11_*), 5.39 (^3^*J*_H,H_ = 15.3 Hz, ^3^*J*_H,H_ = 6.7 Hz, *H_10_*), 5.17 (q, *^3^J_H,H_* = 7.0 Hz, 1H, *H_9_*), 4.12 (q, ^3^*J*_H,H_ = 7.2 Hz, 2H, *H_2’_*), 3.83 – 3.79 (m, 1H, *H_13_*), 3.77 (q, ^3^*J*_H,H_ = 6.5 Hz, 1H, *H_15_*), 2.59 – 2.52 (m, 1H, *H_12_*), 2.32 – 2.28 (m, 1H, *H_14a_*), 2.26 (t, ^3^*J*_H,H_ = 7.6 Hz, 2H, *H_2_*), 2.18 (s, 3H, C*H_3_* OAc), 1.91 (quint, ^3^*J*_H,H_ = 7.7 Hz, 1H, *H_16_*), 1.58 – 1.40 (m, 5H, *H_3_*, *H_8_*, *H_14b_*), 1.33 – 1.27 (m, 8H, *H_4_*, *H_5_*, *H_6_*, *H_7_*), 1.25 (t, ^3^*J*_H,H_ = 7.1 Hz, 3H, *H_3’_*), 1.32 – 1.23 (m, 4H, including t, ^3^*J*_H,H_ = 7.1 Hz, 3H, *H_3’_* at 1.25 and *H_17a_*), 1.22 – 1.08 (m, 7H, *H_4_*, *H_5_*, *H_6_*, *H_17b_*), 1.04 – 0.97 (m, 1H, *H_7_*), 0.88 (s, 9H, C*H_3_* *t*-Bu TBS), 0.85 (s, 9H, C*H_3_* *t*-Bu TBS), 0.81 (t, ^3^*J*_H,H_ = 7.3 Hz, 1H, *H_18_*), 0.03 (s, 3H, C*H_3_* Me-TBS), 0.03 (s, 3H, C*H_3_* Me-TBS), -0.01 (s, 3H, C*H_3_* Me-TBS), -0.02 (s, 3H, C*H_3_* Me-TBS).

^13^C NMR (126 MHz, CDCl_3_): δ 174.0 (*C_1_*), 170.2 (*C*=O), 168.3 (*C*=O), 134.3 (*C_Ar_*), 133.6 (*C_10_*), 129.4 (*C_11_*), 129.3 (*C_Ar_*), 128.8 (2 *C_Ar_*), 127.7 (2 *C_Ar_*), 76.3 (*C_9_*), 76.1 (*C_13_* or *C_15_*), 76.1 (*C_13_* or *C_15_*), 74.8 (Ph-*C*H-OAc), 60.3 (*C_2’_*), 52.7 (*C_12_*), 51.4 (*C_16_*), 44.6 (*C_14_*), 34.5 (*C_2_* or *C_8_*), 34.4 (*C_2_* or *C_8_*), 29.2 (*C_4_* or *C_5_* or *C_6_*), 29.1 (2C, *C_4_* or *C_5_* or *C_6_*), 26.0 (6C, *C*H_3_ t-Bu TBS), 25.0 (*C_3_* or *C_7_*), 24.8 (*C_3_* or *C_7_*), 21.5 (*C_17_*), 20.9 (*C*H*_3_* OAc), 18.2 (*C_q_* TBS), 18.2 (*C_q_* TBS), 14.4 (*C_3’_*), 12.6 (*C_18_*), -4.2 (*C*H_3_ Me-TBS), -4.5 (2C, *C*H_3_ Me-TBS), -4.6 (*C*H_3_ Me-TBS).

#### Determination of absolute configuration of C_9_-stereocenter

|  | (S) | (R) | **δ (S) – δ (R)** |
| --- | --- | --- | --- |
| δ H_10_ | 5.19 | 5.39 | **- 0.20** |
| δ H_8_ | 1.60 | 1.46 | **+ 0.14** |

Based on empirical rules established in the literature^[37]^, we could then confirm the (*S*) configuration of C_9_.

### HPLC analysis and comparison

#### General procedure for saponification of PhytoP-HT and HPLC analysis

A solution of LiOH.H_2_O (1 mg, 23.8 µmol, 11 equiv.) in distilled water (200 µL) was added to a solution of PhytoP-HT (1 mg, 2.15 µmol, 1 equiv.) in THF (200 µL). The mixture was stirred at room temperature for 1 hour and then acidified by the addition of aqueous HCl (0.5 M, 200 µL). The mixture was diluted with MeCN (2 mL), dried over MgSO_4_ and filtered (0.45 µm Chromafil Xtra) prior to HPLC analysis.


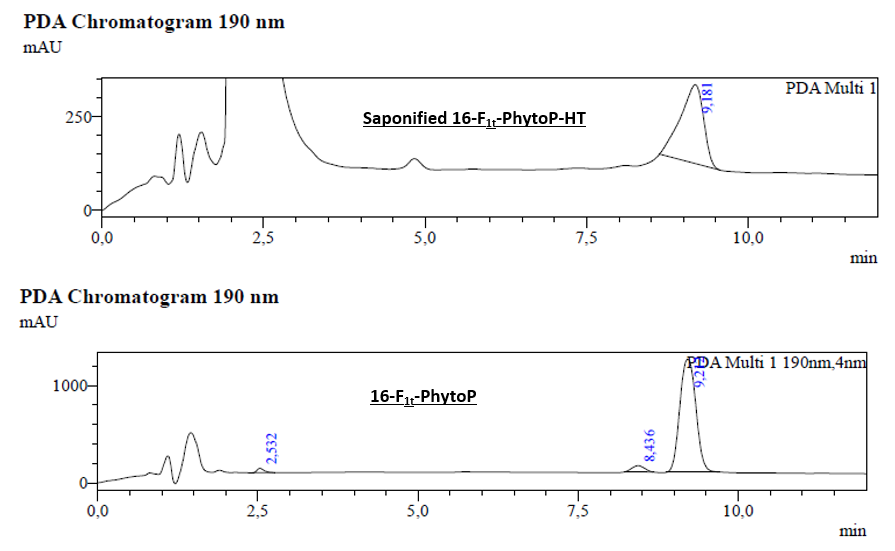


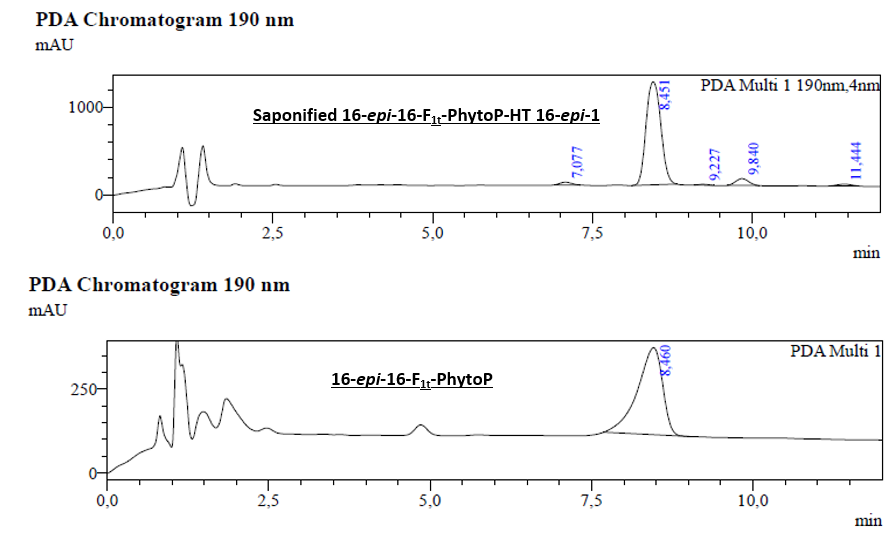


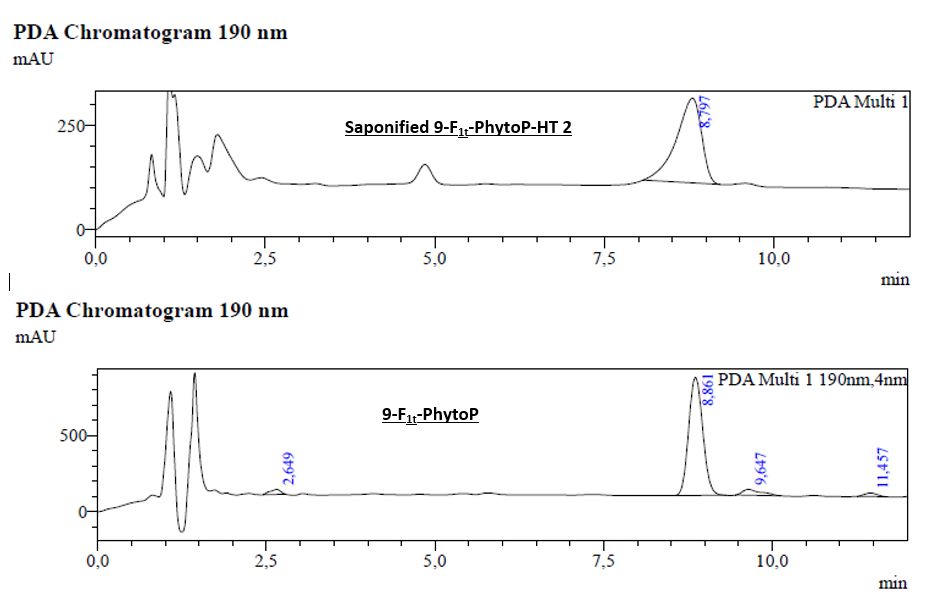


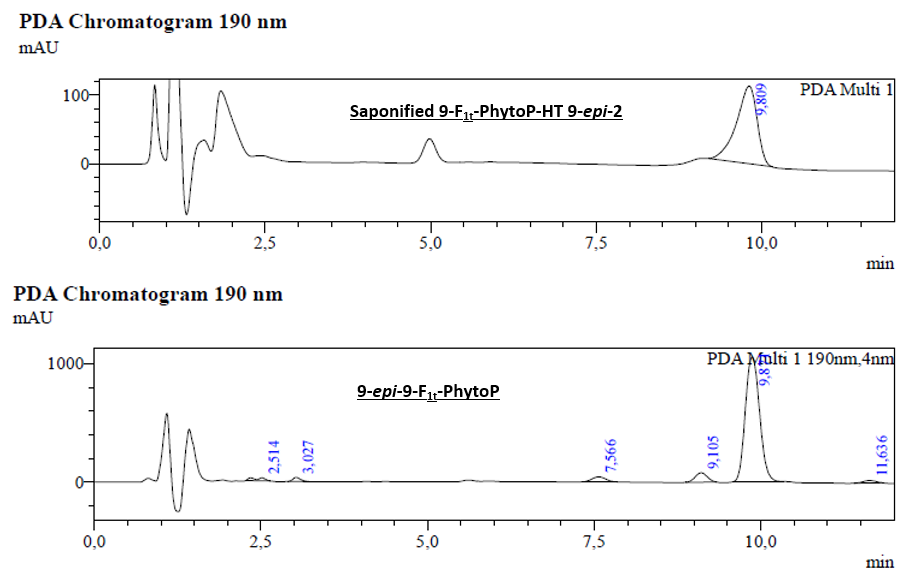


**9-*epi*-9-F_1t_-PhytoP**

# Analytical study

## Preparation of 3,4-dihydroxyphenethyl (9*Z*,12*Z*,15*Z*)-octadeca-9,12,15-trienoate (HT-ALA) (**S23**)

DCC (592 mg, 2.87 mmol, 1.1 equiv.), DMAP (192 mg, 1.57 mmol, 0.6 equiv.) and ALA (800 mg, 2.87 mmol, 1.1 equiv., solution in 2 mL anhydrous CH_2_Cl_2_) were successively added to a solution of **22** (1.0 g, 2.61 mmol, 1 equiv.) in 50 mL anhydrous CH_2_Cl_2_. The mixture was stirred at room temperature overnight and was then filtered on Celite®. The filtrate was concentrated under reduced pressure and the crude was purified by silica gel column chromatography using pentane/Et_2_O 95/5 to 90/10, to afford the pure TBS protected ester (1.5 g) as a colourless oil. In a Teflon® flask, Et_3_N.3HF (1.52 mL, 9.33 mmol, 4 equiv.) was added to a solution of the TBS protected compound (1.5 g, 2.33 mmol, 1 equiv.) in 25 mL anhydrous THF and the mixture was stirred at room temperature for 3 hours. The mixture was then treated by a dropwise addition of a saturated aqueous solution of NaHCO_3_ (until gas formation is over). The crude was extracted by EtOAc (3 x 50 mL), the combined organic layers were washed with brine, dried over MgSO_4_ and concentrated under reduced pressure. The crude was then purified by silica gel column chromatography using a gradient pentane/EtOAc from 80/20 to 70/30, to afford the pure HT-ALA **S23** (905 mg, 2.18 mmol, 84% over 2 steps) as a colourless oil.

Rf = 0.3 (cyclohexane/EtOAc: 7/3).

^1^H NMR (400 MHz, CDCl_3_) δ 6.78 (d, ^3^*J*_H,H_ = 8.0 Hz, 1H), 6.74 (d, ^4^*J*_H,H_ = 2.1 Hz, 1H), 6.66 – 6.62 (m, 1H), 5.56 – 5.20 (m, 8H), 4.23 (t, ^3^*J*_H,H_ = 7.1 Hz, 2H), 2.87 – 2.76 (m, 6H), 2.28 (t, ^3^*J*_H,H_ = 7.5 Hz, 2H), 2.13 – 2.01 (m, 4H), 1.69 – 1.53 (m, 2H), 1.40 – 1.22 (m, 8H), 0.97 (t, ^3^*J*_H,H_ = 7.5 Hz, 3H). In analogy to Lee *et al*. *Chem. Res. Toxicol.* **2016**, 29, 1689–1698.

## General procedure for oxidations with V70

Radical initiator V70 (7.4 mg, 0.024 mmol, 0.2 equiv.) was added to a solution of HT-ALA/HT+ALA (0.12 mmol, 1 equiv.) in 2.4 mL dry MeCN. The mixture was then stirred at 40 °C with an air flux in a sand bath for 24 hours. The crude was concentrated under reduced pressure and was then purified by silica gel column chromatography first with pentane/EtOAc 50/50 and then a gradient EtOAc/MeOH 100/0 to 95/5 to afford two fractions: the apolar fraction containing the unreacted starting material and the polar fractions containing the oxidized products. The polar fractions were dried under reduced pressure and stored at −20°C prior to sample preparation for analysis. Fractions were then dissolved with 1 mL MeCN and further diluted to 500 mg/mL with UHPLC mobile phase (80/20 H_2_O/MeCN).

## UHPLC-HRMS/MS analysis

The UHPLC-HRMS/MS analysis was conducted on a Vanquish LC system coupled to an Orbitrap ID-X Tribrid mass spectrometer, from Thermo Fisher Scientific (Waltham, MA, USA). Instrument configuration and data acquisition was conducted using Xcalibur 4.7 software (Thermo Fisher Scientific). LC separation was achieved on a Kinetex 1.7 µm XB-C18 Core Shell column (50 mm × 2.1 mm i.d.), from Phenomenex (Torrance, CA, USA), maintained at 40 °C. The mobile was composed of H_2_O containing 0.1% *v/v* of formic acid (solvent A), and MeCN containing 0.1% *v/v* of formic acid (solvent B), delivered at a flow rate of 500 µL min^-1^ with a 5 µL injection volume. The following gradient elution program was applied: 0.0 min, 10% B; 0.5 min, 25% B; 4.5 min, 30% B; 5.5 min, 30% B; 7.0 min, 45% B; 8.8 min, 99% B; 11.0 min, 99% B; 11.1 min, 10% B; 12 min, 10% B. The analysis was completed in a total runtime of 12 min, with 1 min of post-run time.

The heated electrospray ionization (H-ESI) source operated in negative ion polarity mode with the following settings: spray voltage, 2500 V; sheath gas, 50 arb; auxiliary gas, 10 arb; sweep gas, 1 arb; ion transfer tube temperature,
325 °C; and vaporizer temperature, 350 °C. For HRMS/MS data acquisition, simultaneous MS^1^ Full Scan (FS) and data-dependent MS/MS (DDA-MS^2^) were performed. MS^1^ FS acquisition was conducted with the subsequent scan parameters: Orbitrap resolution, 60000 FWHM; scan range, 50-1000 m/z; RF lens, 45%; and maximum injection time, 50 ms. For DDA-MS^2^ acquisition, the following scan parameters were used: isolation window, 1 m/z; activation type, HCD; stepped collision energy, 20, 40, 60%; Orbitrap resolution, 30000 FWHM; and maximum injection time, 54 ms. To serve as MS/MS scan triggers, a set of data-dependent conditions were applied, including an ion intensity threshold of 2.0e4, a dynamic exclusion of 2 s, and an inclusion list of 57 target compounds with an exact mass tolerance (ΔMass) of ±5 ppm (Supplementary Table 1). Furthermore, the method was set to perform dependent scans on the most intense ions when no target ions were found. Data processing, chromatogram extraction, and integration were carried out using mzmine version 4.7.

The molecular formulas of the inclusion list were derived from HT-ALA (C_18_H_30_O_2_) through oxidative processes, involving the addition of oxygen atoms, loss of hydrogen atoms, and potential rearrangement of the molecular structure.

Supplementary Table 1. Inclusion list used for DDA-MS^2^ acquisition

| **Compound** | **[M-H]^-^ Exact mass (m/z)** |
| --- | --- |
| C8H8O3 | 151.0401 |
| C8H10O3 | 153.0557 |
| C9H10O3 | 165.0557 |
| C18H30O2 | 277.2173 |
| C18H26O3 | 289.1809 |
| C18H28O3 | 291.1966 |
| C18H30O3 | 293.2122 |
| C18H26O4 | 305.1758 |
| C18H28O4 | 307.1915 |
| C18H30O4 | 309.2071 |
| C18H32O4 | 311.2228 |
| C18H28O5 | 323.1864 |
| C18H30O5 | 325.2020 |
| C18H32O5 | 327.2177 |
| C18H34O5 | 329.2333 |
| C18H26O6 | 337.1657 |
| C18H28O6 | 339.1813 |
| C18H30O6 | 341.1970 |
| C18H32O6 | 343.2126 |
| oxHT-C18H30O2 | 411.2552 |
| HT-C18H30O2 | 413.2697 |
| oxHT-C18H26O3 | 423.2188 |
| HT-C18H26O3 | 425.2333 |
| oxHT-C18H28O3 | 425.2344 |
| HT-C18H28O3 | 427.2490 |
| oxHT-C18H30O3 | 427.2501 |
| HT-C18H30O3 | 429.2646 |
| oxHT-C18H26O4 | 439.2137 |
| HT-C18H26O4 | 441.2282 |
| oxHT-C18H28O4 | 441.2293 |
| HT-C18H28O4 | 443.2439 |
| oxHT-C18H30O4 | 443.2450 |
| HT-C18H30O4 | 445.2595 |
| oxHT-C18H32O4 | 445.2606 |
| HT-C18H32O4 | 447.2752 |
| oxHT2-C18H26O4 | 457.2231 |
| oxHT-C18H28O5 | 457.2242 |
| HT-C18H28O5 | 459.2388 |
| oxHT-C18H30O5 | 459.2399 |
| HT-C18H30O5 | 461.2544 |
| oxHT-C18H32O5 | 461.2555 |
| HT-C18H32O5 | 463.2701 |
| oxHT-C18H34O5 | 463.2712 |
| HT-C18H34O5 | 465.2857 |
| oxHT-C18H26O6 | 471.2035 |
| HT-C18H26O6 | 473.2181 |
| oxHT-C18H28O6 | 473.2192 |
| HT-C18H28O6 | 475.2337 |
| oxHT-C18H30O6 | 475.2348 |
| HT-C18H30O6 | 477.2494 |
| oxHT-C18H32O6 | 477.2505 |
| HT-C18H32O6 | 479.2650 |
| oxHT2-C18H34O5 | 481.2807 |
| oxHT2-C18H26O6 | 489.2130 |
| oxHT2-C18H28O6 | 491.2286 |
| oxHT2-C18H30O6 | 493.2443 |
| oxHT2-C18H32O6 | 495.2599 |

## Distribution of PhytoPs-HT and PhytoPs in oxidized sample

Extracted ion chromatograms (EICs) were generated for each target compound by selecting the exact monoisotopic mass corresponding to the deprotonated molecular ion [M–H]–, using a mass tolerance of ±5 ppm. Specifically, m/z 463.2701 [C_26_H_40_O_7_–H]– for PhytoPs-HT and m/z 327.2177 [C18H32O5–H]– for unesterified PhytoPs. Retention times and MS/MS fragmentation patterns of each detected feature were verified by comparison with individually injected authentic standards analyzed under identical chromatographic and mass spectrometric conditions to ensure accurate identification. In Supplementary Table 2 the retention times and the exact mass at MS1 feature apex are shown.

For each compound, chromatographic peak areas were integrated at the corresponding retention time. The areas of all eight annotated compounds were summed, and the area of each individual peak was expressed as a percentage of this total. This approach provides a comparative semiquantitative distribution of the detected compounds across the different oxidation conditions tested. No internal standards were included, and no external calibration was performed; therefore, absolute quantification was not undertaken.

Data processing, chromatogram extraction, and integration were carried out using mzmine version 4.7.

Supplementary Table 2. Exact mass and retention time of the standards analyzed.

| **Compound** | **Retention time [min]** | **Exact mass at feature apex [m/z]** |
| --- | --- | --- |
| 16-*epi*-16-F_1t_-PhytoP | 2.28 | 327.2174 |
| 9-F_1t_-PhytoP | 2.31 | 327.2174 |
| 16-F_1t_-PhytoP | 2.36 | 327.2174 |
| 9-*epi*-9-F1t-PhytoP | 2.39 | 327.2174 |
| 16-*epi*-16-F_1t_-PhytoP-HT | 4.87 | 463.2697 |
| 9-F_1t_-PhytoP-HT | 4.97 | 463.2698 |
| 16-F_1t_-PhytoP-HT | 5.06 | 463.2699 |
| 9-*epi*-9-F_1t_-PhytoP-HT | 5.15 | 463.2698 |

Supplementary Table 3. Retention time and cosine score of the PhytoPs-HT.

| **Compound** | **Retention time [min]** | **Cosine score** |
| --- | --- | --- |
| 16-*epi*-16-F_1t_-PhytoP-HT | 4.87 | 0.9833 |
| 9-F_1t_-PhytoP-HT | 4.97 | 0.9981 |
| 16-F_1t_-PhytoP-HT | 5.06 | 0.9936 |
| 9-*epi*-9-F_1t_-PhytoP-HT | 5.15 | 0.9911 |

All the chromatographic and mass spectrometric data generated in this work are available in Zenodo repository (<https://doi.org/10.5281/zenodo.15792096>).

## MS/MS spectra at m/z 463.2701 and cosine score

Comparison of MS/MS spectra extracted at 4.86, 4.98, 5.05, and 5.16 min from EIC, with MS/MS spectra of PhytoPs-HT standards (in green): Cosine scores for each unknown signal were calculated by comparing their MS/MS spectra with those of synthetic PhytoPs-HT standards. All analysis were performed using UHPLC-HRMS/MS (Orbitrap ID-X Tribrid, Thermo Fisher Scientific) in data-dependent acquisition (DDA) mode.


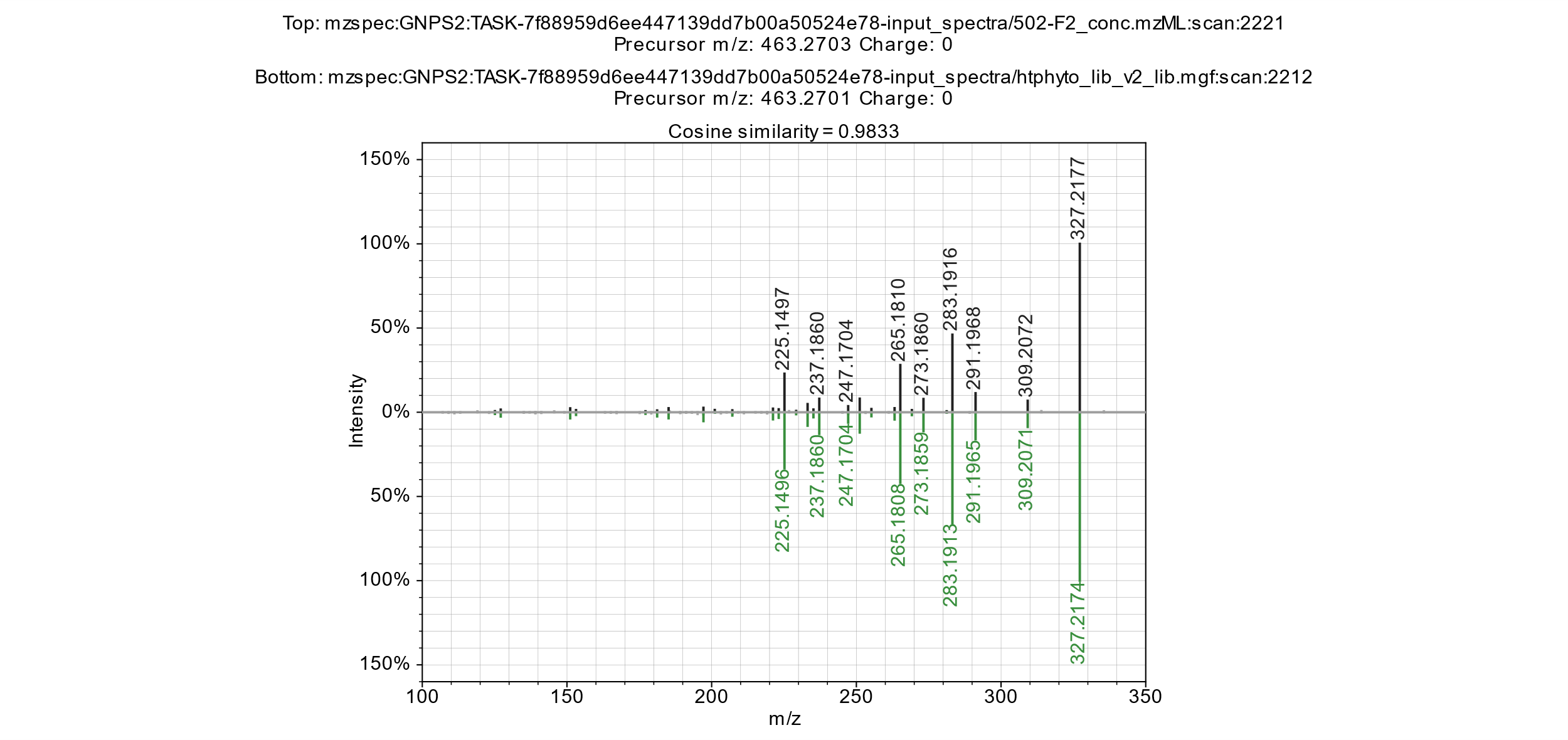


**Cosine score: 0.9833**

**16-*epi*-16-F_1t_-PhytoP-HT**

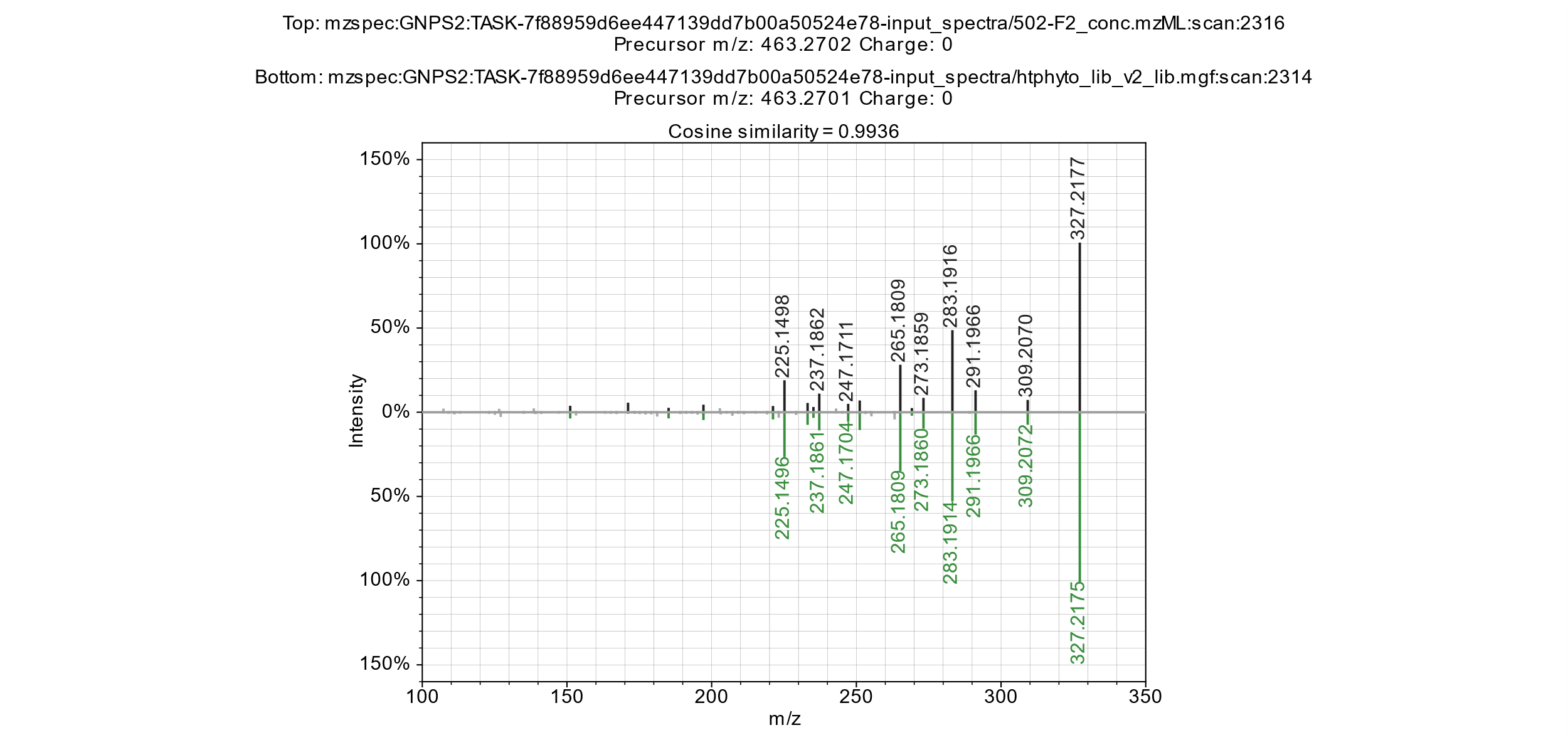


**Cosine score: 0.9936**

**16-F_1t_-PhytoP-HT**

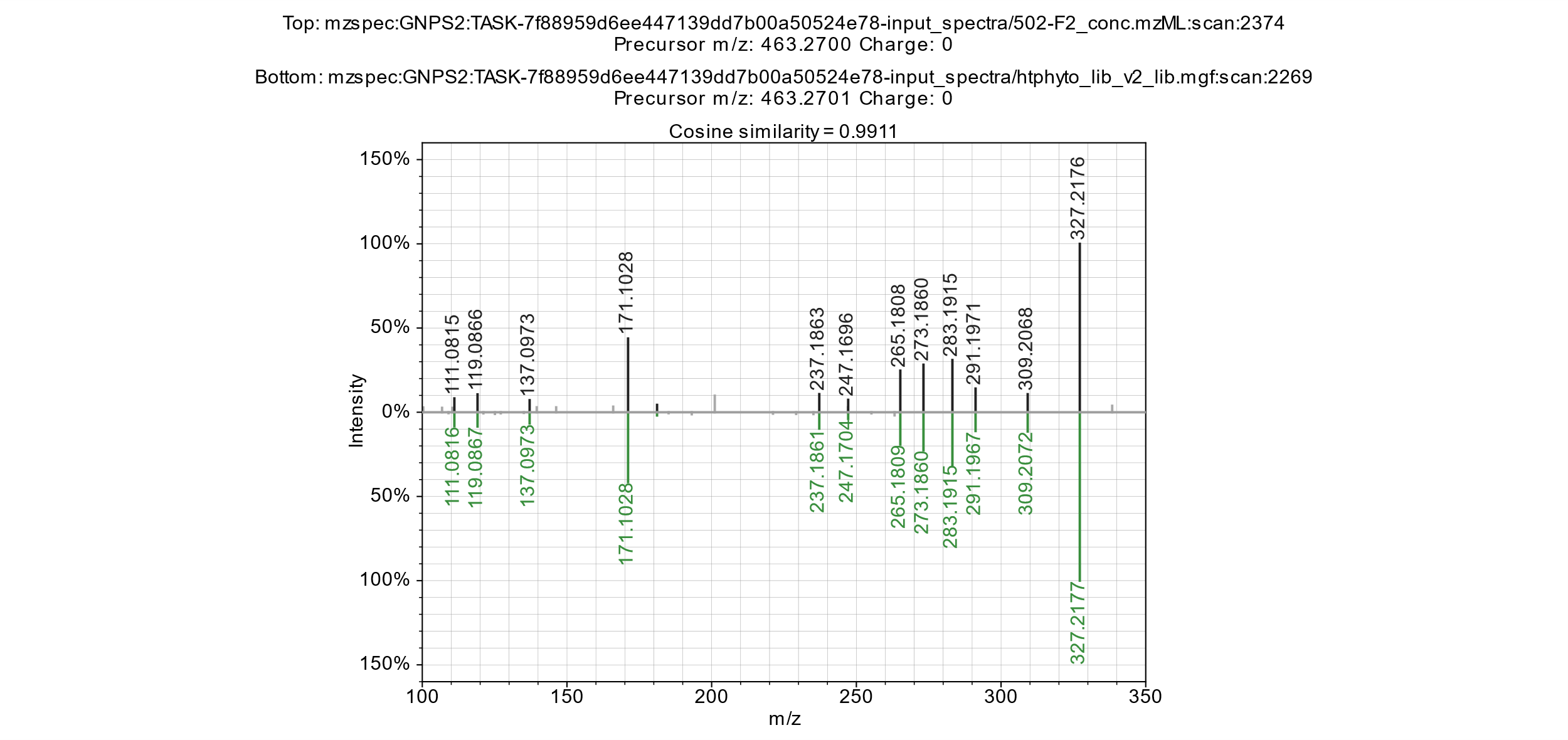


**Cosine score: 0.9911**

**9-*epi*-9-F_1t_-PhytoP-HT**

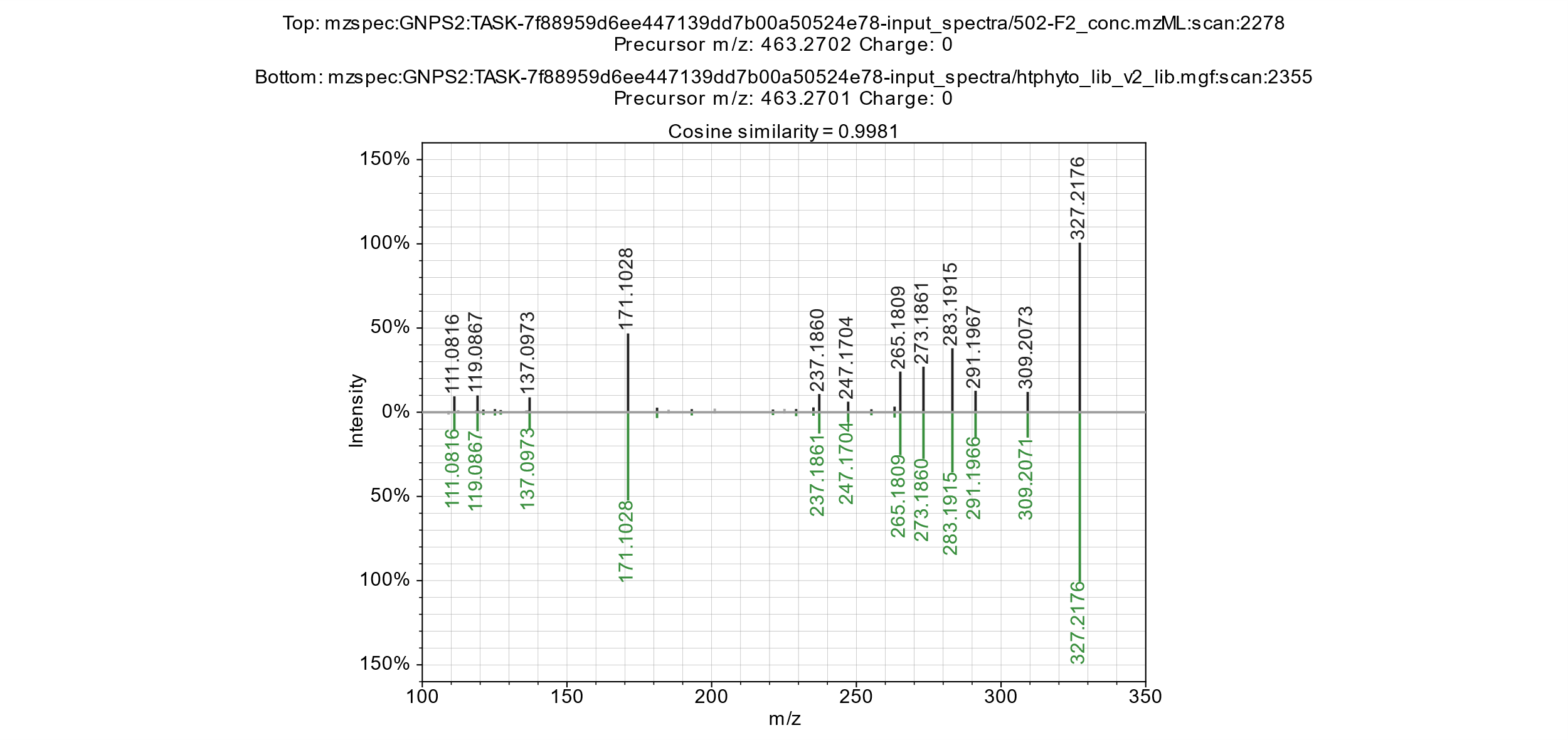


**Cosine score: 0.9981**

**9-F_1t_-PhytoP-HT**

# NMR Spectra and HPLC chromatograms

**1-hydroxy-1-oxo-1l5-benzo[d][1,2]iodaoxol-3(1H)-one (S1)**

**methyl 2-(3,4-dihydroxyphenyl)acetate (S3)**

**methyl 2-(3,4-bis((tert-butyldimethylsilyl)oxy)phenyl)acetate (S4)**


**2-(3,4-bis((tert-butyldimethylsilyl)oxy)phenyl)ethan-1-ol (22)**

**ethyl 10-(dimethoxyphosphoryl)-9-oxodecanoate (24)**

**(Z)-9-oxabicyclo[6.1.0]non-2-ene (S5)**

**(1S,3aS,6aS)-1,2,3,3a,4,6a-hexahydropentalen-1-ol (rac-8)**

**(3aR,6aR)-4,6a-dihydropentalen-1(3aH)-one (9)**

**(1aR,1bS,4aR,5aR)-1a,2,4a,5a-tetrahydropentaleno[1,2-b]oxiren-5(1bH)-one (10)**

**(1R,3S,3aS,6aR)-1,2,3,3a,4,6a-hexahydropentalene-1,3-diol (11)**

**(((1R,3S,3aS,6aR)-1,2,3,3a,4,6a-hexahydropentalene-1,3-diyl)bis(oxy))bis(tert-butyldimethylsilane) (S7)**

**2-((1S,2S,3R,5S)-3,5-bis((tert-butyldimethylsilyl)oxy)-2-(hydroxymethyl)cyclopentyl)ethan-1-ol (12)**

**(4aS,5S,7R,7aS)-5,7-bis((tert-butyldimethylsilyl)oxy)hexahydrocyclopenta[c]pyran-3(1H)-one (13)**

**(4aS,5S,7R,7aS)-5,7-bis((tert-butyldimethylsilyl)oxy)octahydrocyclopenta[c]pyran-3-ol (5)**


**2-((1R,2R,3S,5R)-3,5-bis((tert-butyldimethylsilyl)oxy)-2-(hydroxymethyl)cyclopentyl)ethyl acetate (S8)**
**2-((1R,2R,3S,5R)-3,5-bis((tert-butyldimethylsilyl)oxy)-2-(hydroxymethyl)cyclopentyl)ethyl acetate (14)**


**2-((1R,2R,3S,5R)-3,5-bis((tert-butyldimethylsilyl)oxy)-2-(((triethylsilyl)oxy)methyl)cyclopentyl)ethyl acetate (6)**

**methyl (Z)-8-((1S,2S,3R,5S)-3,5-bis((tert-butyldimethylsilyl)oxy)-2-(hydroxymethyl)cyclopentyl)oct-6-enoate (19)**

**methyl 8-((1S,2S,3R,5S)-3,5-bis((tert-butyldimethylsilyl)oxy)-2-(hydroxymethyl)cyclopentyl)octanoate (S9)**

**methyl 8-((1S,2R,3R,5S)-3,5-bis((tert-butyldimethylsilyl)oxy)-2-formylcyclopentyl)octanoate (S10)**

**methyl 8-((1S,2R,3R,5S)-3,5-bis((tert-butyldimethylsilyl)oxy)-2-((E)-3-oxopent-1-en-1-yl)cyclopentyl)octanoate (21)**

**8-((1S,2R,3R,5S)-3,5-bis((tert-butyldimethylsilyl)oxy)-2-((E)-3-oxopent-1-en-1-yl)cyclopentyl)octanoic acid (S11)**

**3,4-bis((tert-butyldimethylsilyl)oxy)phenethyl 8-((1S,2R,3R,5S)-3,5-bis((tert-butyldimethylsilyl)oxy)-2-((E)-3-oxopent-1-en-1-yl)cyclopentyl)octanoate (23)**

**3,4-bis((tert-butyldimethylsilyl)oxy)phenethyl 8-((1S,2R,3R,5S)-3,5-bis((tert-butyldimethylsilyl)oxy)-2-((S,E)-3-hydroxypent-1-en-1-yl)cyclopentyl)octanoate (S12)**

**3,4-bis((tert-butyldimethylsilyl)oxy)phenethyl 8-((1S,2R,3R,5S)-3,5-bis((tert-butyldimethylsilyl)oxy)-2-((R,E)-3-hydroxypent-1-en-1-yl)cyclopentyl)octanoate (*16-epi*-S12)**

**3,4-dihydroxyphenethyl 8-((1S,2R,3R,5S)-3,5-dihydroxy-2-((S,E)-3-hydroxypent-1-en-1-yl)cyclopentyl)octanoate (1)**


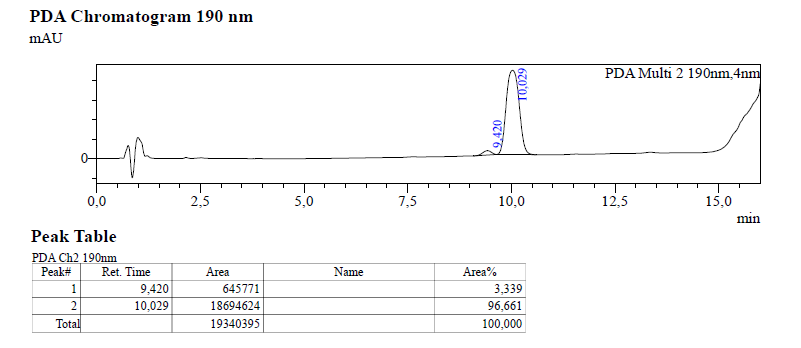


The diastereomeric ratio is 97:3 in favor of the (*S*)-epimer at the C16 center.

**3,4-dihydroxyphenethyl 8-((1S,2R,3R,5S)-3,5-dihydroxy-2-((R,E)-3-hydroxypent-1-en-1-yl)cyclopentyl)octanoate (*16-epi*-1)**


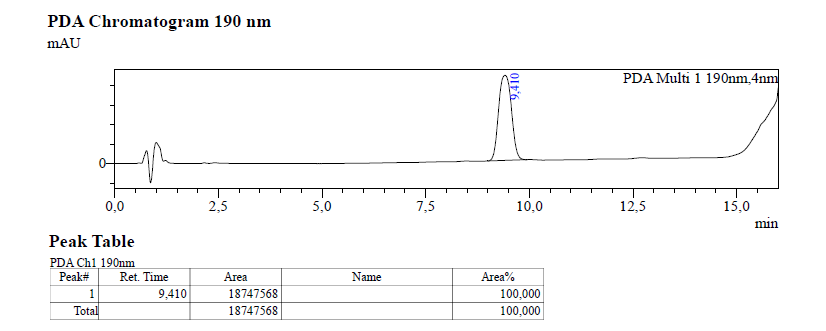


The diastereomeric ratio is superior at 99:1 in favor of the (*R*)-epimer at the C16 center.

**8-((1S,2R,3R,5S)-3,5-dihydroxy-2-((S,E)-3-hydroxypent-1-en-1-yl)cyclopentyl)octanoic acid -16-F_1t_-PhytoP (S13)**

**8-((1S,2R,3R,5S)-3,5-dihydroxy-2-((R,E)-3-hydroxypent-1-en-1-yl)cyclopentyl)octanoic acid – 16-*epi*-16-F_1t_-PhytoP (16-*epi*-S13)**

**2-((1R,2R,3S,5R)-3,5-bis((tert-butyldimethylsilyl)oxy)-2-(((triethylsilyl)oxy)methyl)cyclopentyl)ethyl 4-methylbenzenesulfonate (S14)**

**((1S,3R,4R,5R)-4-ethyl-5-(((triethylsilyl)oxy)methyl)cyclopentane-1,3-diyl)bis(oxy))bis(tert-butyldimethylsilane) (4)**

**(1S,2R,3R,5S)-3,5-bis((tert-butyldimethylsilyl)oxy)-2-ethylcyclopentane-1-carbaldehyde (S15)**

**ethyl (E)-11-((1S,2R,3R,5S)-3,5-bis((tert-butyldimethylsilyl)oxy)-2-ethylcyclopentyl)-9-oxoundec-10-enoate (25)**

**3,4-bis((tert-butyldimethylsilyl)oxy)phenethyl (E)-11-((1S,2R,3R,5S)-3,5-bis((tert-butyldimethylsilyl)oxy)-2-ethylcyclopentyl)-9-oxoundec-10-enoate (S16)**

**3,4-bis((tert-butyldimethylsilyl)oxy)phenethyl (S,E)-11-((1S,2R,3R,5S)-3,5-bis((tert-butyldimethylsilyl)oxy)-2-ethylcyclopentyl)-9-hydroxyundec-10-enoate (S17)**

**3,4-bis((tert-butyldimethylsilyl)oxy)phenethyl (R,E)-11-((1S,2R,3R,5S)-3,5-bis((tert-butyldimethylsilyl)oxy)-2-ethylcyclopentyl)-9-hydroxyundec-10-enoate (9-*epi*-S17)**

**3,4-dihydroxyphenethyl (S,E)-11-((1S,2R,3R,5S)-2-ethyl-3,5-dihydroxycyclopentyl)-9-hydroxyundec-10-enoate (2)**


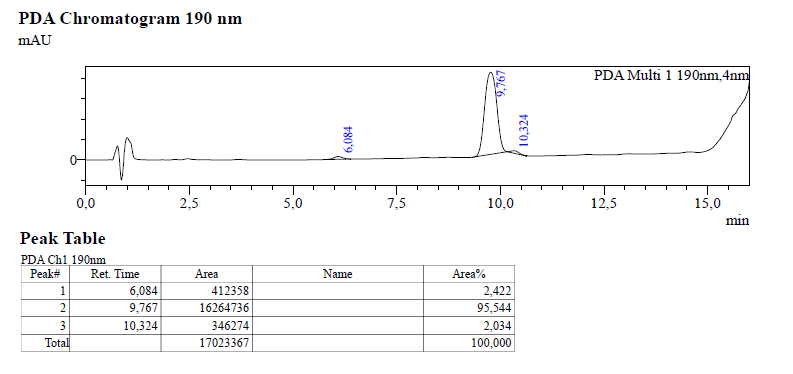


The diastereomeric ratio is 98:2 in favor of the (*S*)-epimer at the C9 center.

**3,4-dihydroxyphenethyl (R,E)-11-((1S,2R,3R,5S)-2-ethyl-3,5-dihydroxycyclopentyl)-9-hydroxyundec-10-enoate (9-*epi*-2)**


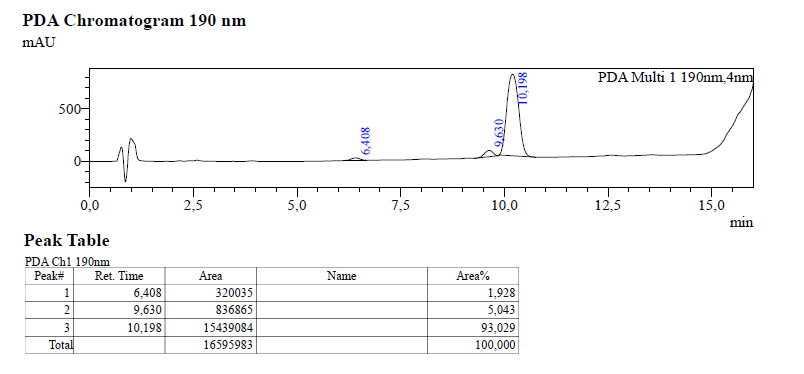


The diastereomeric ratio is 95:5 in favor of the (*R*)-epimer at the C9 center.

**(S,E)-11-((1S,2R,3R,5S)-2-ethyl-3,5-dihydroxycyclopentyl)-9-hydroxyundec-10-enoic acid-** **9-F_1t_-PhytoP (S19)**

**(R,E)-11-((1S,2R,3R,5S)-2-ethyl-3,5-dihydroxycyclopentyl)-9-hydroxyundec-10-enoic acid 9-*epi*-F_1t_-PhytoP (9-*epi*-S19)**

**3,4-dihydroxyphenethyl (9Z,12Z,15Z)-octadeca-9,12,15-trienoate (HT-ALA) (S23)**

**ethyl (S,E)-11-((1S,2R,3R,5S)-3,5-bis((tert-butyldimethylsilyl)oxy)-2-ethylcyclopentyl)-9-hydroxyundec-10-enoate (S20)**

**ethyl (S,E)-9-((S)-2-acetoxy-2-phenylacetoxy)-11-((1S,2R,3R,5S)-3,5-bis((tert-butyldimethylsilyl)oxy)-2-ethylcyclopentyl)undec-10-enoate (S21)**

**ethyl (S,E)-9-((R)-2-acetoxy-2-phenylacetoxy)-11-((1S,2R,3R,5S)-3,5-bis((tert-butyldimethylsilyl)oxy)-2-ethylcyclopentyl)undec-10-enoate (S22)**
